# Development and validation of a VHL-associated immune prognostic signature for clear cell renal cell carcinoma

**DOI:** 10.1186/s12935-020-01670-5

**Published:** 2020-12-07

**Authors:** Jin Zhang, Aiting Yan, Wei Cao, Honglei Shi, Kai Cao, Xiaowu Liu

**Affiliations:** 1 Department of Urology Surgery, Changzhou Wujin People’s Hospital, Wujin Hospital Affiliated Jiangsu University, The Wujin Clinical College of Xuzhou Medical University, Yongning north road 2, Tianning, Changzhou, 213000 People’s Republic of China; 2 Department of General Practice, The Affiliated Geriatric Hospital of Nanjing Medical University, Nanjing, 210009 People’s Republic of China; 3 Department of Oncology, Affiliated Haian Hospital of Nantong University, Nantong, Jiangsu 226600 People’s Republic of China

**Keywords:** Clear cell renal cell carcinoma (ccRCC), VHL mutation, Tumour-infiltrating

## Abstract

**Background:**

VHL mutation is the most common mutation in clear cell renal cell carcinoma (ccRCC). Here, we developed and validated an immune-related signature to predict the prognosis of ccRCC with VHL mutations.

**Methods:**

VHL mutation status and RNA expression were analysed in the TCGA datasets and our cohort. LASSO Cox analysis was performed to develop an immune-related signature. Candidate genes for the immune-related signature were differentially expressed between VHLwt and VHLmut ccRCC patients.

**Results:**

VHL mutations resulted in the downregulation of the immune response in ccRCC. To develop an immune-related signature, LASSO Cox analysis was constructed by immune-related genes that were differentially expressed between VHLwt (WHL wild type) and VHLmut (VHL mutation) ccRCC patients. The signature was developed and validated in the TCGA and our own cohort to classify patients into groups based on having a low or high risk of poor survival. Functional enrichment analysis showed that the immune-related pathway represented the major function and pathway. In addition, patients in the high-risk group had a positive correlation with low fractions of CD4 + T cells and dendritic cells and presented a lower expression of CTLA-4 and PD-1 than the low-risk group.

**Conclusion:**

In this study, we proposed a novel immune-related signature, which is a feasible biomarker for predicting the overall survival in VHLmut patients with ccRCC.

## Background

Renal cell carcinoma (RCC) is the second most common malignancy in the urinary system and accounts for more than 90% of kidney malignancies [1]. In the United States, a total of 73,750 new cancer cases and 14,830 deaths from kidney and renal pelvis cancers were estimated in 2020 [2]. Clear cell renal cell carcinoma (ccRCC) is the most common histologic subtype of RCC based on pathologic classification, which accounts for approximately 70% of all RCC cases [3]. The carcinogenesis of ccRCC is a complex process mediated by various drivers and environmental risk factors, such as obesity and smoking [3]. Recently, immune checkpoint inhibitors (ICIs), such as anti-PD-1/PD-L1 inhibitors, have emerged as a significant therapeutic approach for advanced and metastatic ccRCC [4]. However, only a fraction of patients benefit from treatment with ICIs. Although detection of tumour and/or immune cell PD-L1 by immunohistochemistry or tumour mutation burden (TMB) has been investigated as a potential biomarker for response to ICIs, the prediction value has not been determined, and conflicting results have been reported [5]. Several studies have indicated that the tumour microenvironment (TME) stimulates a significant immune response, which might be associated with the immune status and determines the outcomes of ICIs [6, 7]. Thus, a gene expression signature via tumour microenvironment parameters to reliably predict immune response and cancer prognosis that identify a subset of patients with worse survival and effective immune response for additional clinical therapy needs to be constructed.

The von Hippel-Lindau (VHL) gene is located on chromosome 3p, and copy loss occurs in the majority of sporadic ccRCCs [8]. Mutations in the von Hippel-Lindau (VHL) tumour suppressor have been reported in approximately 80% of ccRCC and identified as one of the genetic determinants driving ccRCC initiation and progression [9]. VHL is a component of the E3 ubiquitin ligase complex and functions as a negative regulator of hypoxia inducible factor (HIF) signalling by targeting HIF-1/2α [10]. Loss of VHL function causes a release of HIF-1/2α from VHL-mediated ubiquitination and degradation, which allows for the accumulation of HIF-1/2α, thereby driving transcriptional activation of its downstream target genes related to metabolism, cell cycle and angiogenesis, in turn contributing to ccRCC development [11]. Thus, VHL copy loss might be a promising drug target for ccRCC, which has yielded an efficacious therapy targeting mTOR for the treatment of metastatic ccRCC.

In this study, we performed a comprehensive analysis on the mutation status of VHL and RNA expression to explore the relationship between VHL mutations and immune responses in ccRCC. Our immune prognostic model based on VHL mutations can be used as an important prognostic model and might assist in patient management as potential therapeutic biomarkers for ccRCC.

## Methods and materials

### Acquiring information from public databases

The somatic nonsynonymous mutation information for 403 KIRC patients (workflow type: Mutect2 pipeline), RNA expression profile (workflow type: HTSeq-Counts) and the patients’ clinical information from the Cancer Genome Atlas (TCGA) website (https://gdc.cancer.gov/) were downloaded using the “TCGAbiolinks” R package (Version 2.14.1). Among these KIRC patients, 343 KIRC patients with RNA-sequencing data and TP53 mutation information were subjected to subsequent analyses. Using the “maftools” R package (pipelines = mutect2) (Version 2.2.10), we identified the mutation status of these KIRC patients, which included 173 VHL^MUT^ patients and 170 VHL^WT^ patients. Genes with low RNA expression were removed, thus, we removed transcripts whose counts were 0 in all samples.

### Selecting differentially expressed genes

The “DESeq2” R package (Version 1.26.0) with standard comparison mode between the two experimental conditions was used for differential expression analysis used. The log2|fold change|> 1 and adj. *P* < 0.05 was set as the cut-off value to screen for differentially expressed genes (DEGs) between VHL^mut^ and VHL^wt^ patients. Immune-related DEGs overlapped with DEGs and the ImmPort gene list (http://www.immport.org), a gene list containing over 4000 genes that participate in immune biological processes. Entrez IDs were transformed to gene symbols using the “org.Hs.eg.db” R package (Version 3.10.0).

### Constructing an immune-related risk score

After identifying immune-related DEGs, a Least Absolute Shrinkage and Selector Operation (LASSO) algorithm, using the R package “glmnet” (Version 3.0), with penalty parameter tuning conducted by tenfold cross-validation, was built to select candidate genes. All patients were divided into high- and low-index groups based on the cut-off value that was identified by the “surv_cutpoint” function of the “survminer” R package (Version 0.4.6). Kaplan–Meier survival curves were created with the R package “survival” package (Version 3.5). The following time-dependent receiver operating characteristic (ROC) curve analysis was conducted by the “timeROC” R package (Version 0.4).

RNA extraction and quantitative reverse-transcription polymerase chain reaction was performed on samples from a cohort of patients from the Jiangsu University Affiliated Wujin Hospital and the Affiliated Geriatric Hospital of Nanjing Medical University. A total of 119 patients who underwent surgery without neoadjuvant chemotherapy that were diagnosed with kidney renal clear cell carcinoma at Jiangsu University Affiliated Wujin Hospital and the Affiliated Geriatric Hospital of Nanjing Medical University. The study was approved by the Regional Ethics Committee at Jiangsu University Affiliated Wujin Hospital and the Affiliated Geriatric Hospital of Nanjing Medical University. The experiments were performed with the understanding and written consent of each patient. The study methodologies conformed to the standards set by the Declaration of Helsinki. These patient samples consisted of formalin-fixed paraffin-embedded (FFPE) specimens collected from radical surgery between 2011 and 2013. Each patient underwent a standard radical surgical procedure, and all specimens were evaluated by expert pathologists. Total RNA was extracted from FFPE specimens by manual microdissection using the RNeasy FFPE Kit (Qiagen, Hilden, Germany). Complementary DNA (cDNA) synthesis was performed using PrimeScript RT Master Mix (RR036A) (TaKaRa, Dalian, China). Quantitative reverse-transcriptase polymerase chain reaction (qRT-PCR) assays were performed using the ViiA 7 Dx RT-PCR System (Applied Biosystems, Foster City, Canada) and PowerUp SYBR Green Master Mix (Applied Biosystems, Vilnius, Lithuania). The cycling conditions were as follows: 40 cycles of 95 °C for 15 s and 60 °C for 60 s. The relative expression of target genes was normalized against GAPDH using the 2-ΔCT method. Primer sequences are provided in Additional file 1: Table S1.

### Function enrichment and principal component analysis (PCA)

Metascape (https://metascape.org/) was used to perform functional and pathway enrichment analyses to determine the potential molecular mechanisms of the selected genes. Metascape was used to perform Gene Ontology (GO) and Kyoto Encyclopedia of Genes and Genomes (KEGG) pathway analyses for immune-related DEGs. Principal component analysis was carried out using the “pca3d” R package (Version: 0.10.1) to investigate the gene expression patterns of high- and low-risk patients.

### TIMER website analysis

The TIMER website tool (https://cistrome.shinyapps.io/timer/) is a comprehensive resource to analyse and visualize immune infiltrates among different cancer types. TIMER analyses the gene expression profile from the TCGA to estimate 6 main immune cell types in the tumour microenvironment.

### Statistical analysis

Statistical analyses were performed using R (Version 3.6.2) and GraphPad Prism 8. Student’s *t*-test was used to determine differences in comparisons between 2 groups. All statistical tests were two-tailed with a statistical significance level set at 0.05 in this study.

## Results

### Mutation landscape of KIRC patients in the TCGA-KIRC cohort

**Fig.1:**
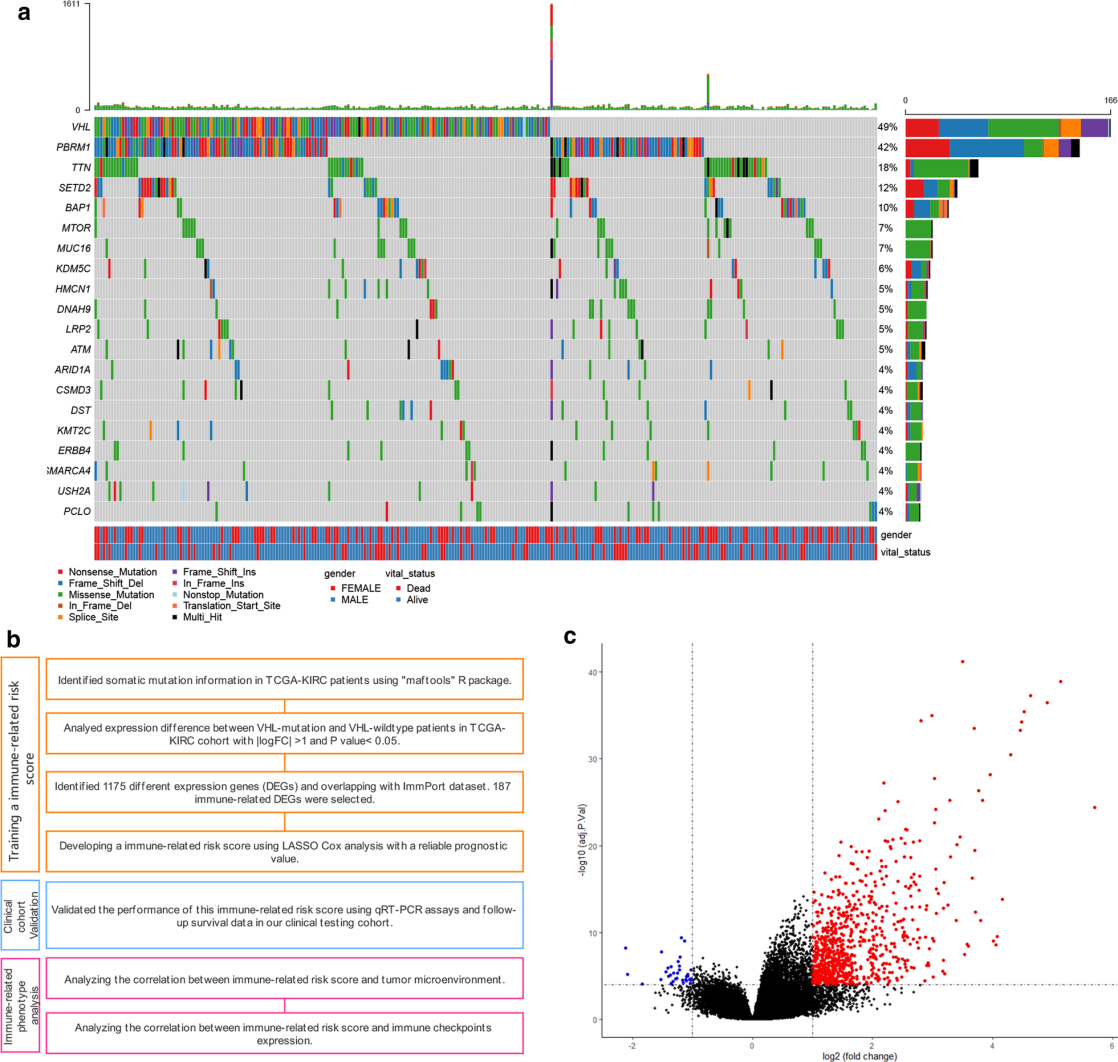
**a** The somatic mutation landscape of kidney renal clear cell carcinoma in the TCGA dataset. **b** The study designs. **c** A volcano plot showing the differentially expressed genes between VHL^MUT^ and VHL^WT^ patients. Red indicates statistical significantly higher expressed, blue indicates statistical significantly lower expressed (|Log FC|> 1, *P* value < 0.05)

In kidney renal clear cell carcinoma, the VHL mutation was shown to be the most common somatic mutation (Fig. 1a). In studies of other tumours, the somatic nonsynonymous mutation status may change the immune response of patients after immunotherapy [12], thus affecting the overall survival of patients with advanced tumours. First, we analysed the overall survival of ccRCC patients with different VHL statuses and the distribution of VHL status in different pathological stages or Fuhrman grades in the TCGA-KIRC data set (Additional file 2: Fig. S1). The results showed that there was no significant difference in overall survival between VHL wild-type and VHL mutant patients. At the same time, there was no significant difference in the distribution of VHL status in different pathological stages and Fuhrman grades. This suggests that somatic mutations enhance the antigenicity of tumours, which may increase the recognition and presentation by peripheral immune infiltrating cells while increasing the malignant phenotype.

Therefore, to comprehensively assess the relationship between immune status and VHL mutation status in patients with KIRC, we conducted the following analysis in the TCGA-KIRC cohort and validation cohort from the Jiangsu University Affiliated Wujin Hospital and the Affiliated Geriatric Hospital of Nanjing Medical University (Fig. 1b). In the TCGA-KIRC cohort, 343 KIRC patients were divided into the VHL^MUT^ (173 patients) and VHL^WT^ (170patients) groups according to VHL mutation status. After that, we screened for significantly differentially expressed genes (DEGs) in the RNA expression profiles of these two groups of patients, which showed that 1175 genes, including mRNAs, lncRNAs, miRNAs and pseudogenes, were significantly differentially expressed (Fig. 1c). These DEGs contained 94 downregulated genes and 1081 upregulated genes (Additional file 1: Table S2).

In addition, to explore the correlation of DEGs between the VHL^MUT^ and VHL^WT^ groups to immune-related phenotypes, we further filtered the RNA expression profile using the ImmPort gene list. We obtained 187 immune-related DEGs by overlapping the DEGs and ImmPort genes list (Additional file 1: Table S3). The Metascape online tool was used to annotate the potential functional characteristics, which identified immune-related DEGs. We can significantly observe that several immune-related pathways are enriched (Additional file 3: Fig. S2), suggesting that we can further analyse potential immune subtypes.

### Construction of an immune-related risk signature

**Fig.2:**
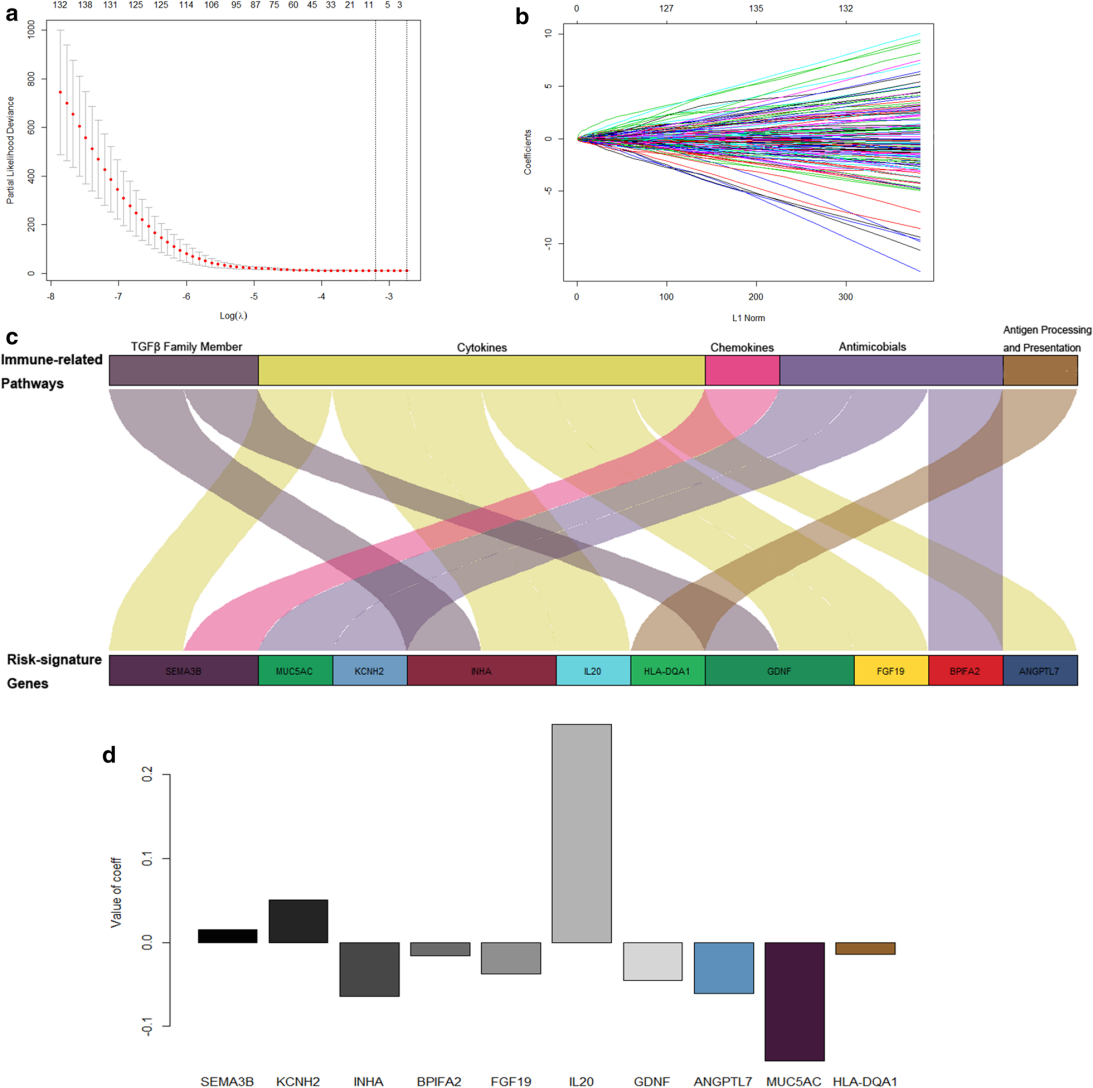
**a** Tuning parameter (lambda) screening in the LASSO regression model. **b** The LASSO coefficient profiles of the common genes. **c** The immune-related pathways enriched by immune-related risk signature genes. **d** The value of coefficient for each of the ten selected genes

To explore the predictive power of immune phenotypes for overall survival, we further analysed the correlation between 187 immune-related DEGs and overall survival. Ten genes, namely, SEMA3B, KCNH2, INHA, BPIFA2, FGF19, IL20, GDNF, ANGPTL7, MUC5AC and HLA-DQA1, were filtered using nonzero regression coefficients that have a maximum prognostic value according to LASSO Cox regression analysis (Fig. 2a , b). These 10 candidate genes are clearly involved in immune-related biological processes or directly participate in immune responses and included TGF-family members, cytokines, chemokines, antimicrobials, antigen processing and presentation (Fig. 2c, Additional file 1: Table S4). This suggests that the difference in the expression of these genes may predict the difference in tumour immune status and tumour microenvironment in patients with KIRC. Finally, a ten-gene immune-related risk score was constructed, and the risk score of each patient was calculated using the following formula: = 0.01488135* (normalized expression of SEMA3B) + (0.05056229* normalized expression of KCNH2) + (− 0.0645472* normalized expression of INHA) + (− 0.01586218* normalized expression of BPIFA2) + (− 0.03727866* normalized expression of FGF19) + (0.25913417* normalized expression of IL20) + (− 0.04517044* normalized expression of GDNF) + (− 0.06116952* normalized expression of ANGPTL7) + (− 0.14067171* normalized expression of MUC5AC) + ( − 0.01418558* normalized expression of HLA-DQA1). The brief formula is as follows: $\mathbf{I}\mathbf{m}\mathbf{m}\mathbf{u}\mathbf{n}\mathbf{e}\mathbf{r}\mathbf{e}\mathbf{l}\mathbf{a}\mathbf{t}\mathbf{e}\mathbf{d}\mathbf{r}\mathbf{i}\mathbf{s}\mathbf{k}\mathbf{s}\mathbf{c}\mathbf{o}\mathbf{r}\mathbf{e}=\sum_{i=1}^{10}{{\varvec{C}}{\varvec{o}}{\varvec{e}}{\varvec{f}}{\varvec{f}}}_{i}*({{\varvec{N}}{\varvec{o}}{\varvec{r}}{\varvec{m}}{\varvec{a}}{\varvec{l}}{\varvec{i}}{\varvec{z}}{\varvec{e}}\boldsymbol{ }{\varvec{E}}{\varvec{x}}{\varvec{p}}{\varvec{r}}{\varvec{e}}{\varvec{s}}{\varvec{s}}{\varvec{i}}{\varvec{o}}{\varvec{n}})}_{i}$

The coefficients of each gene are shown in Fig. 2d. The optimal cut-off value was determined by the “surv_cutpoint” function of the “survminer” R package, and the optimal cut-off value was − 0.5379904. The cut-off value in the TCGA-KIRC cohort was used to assign patients into the high-risk or low-risk group across all KIRC patients in the following analysis.

### Assess the prognostic ability of the immune-related risk score

**Fig.3:**
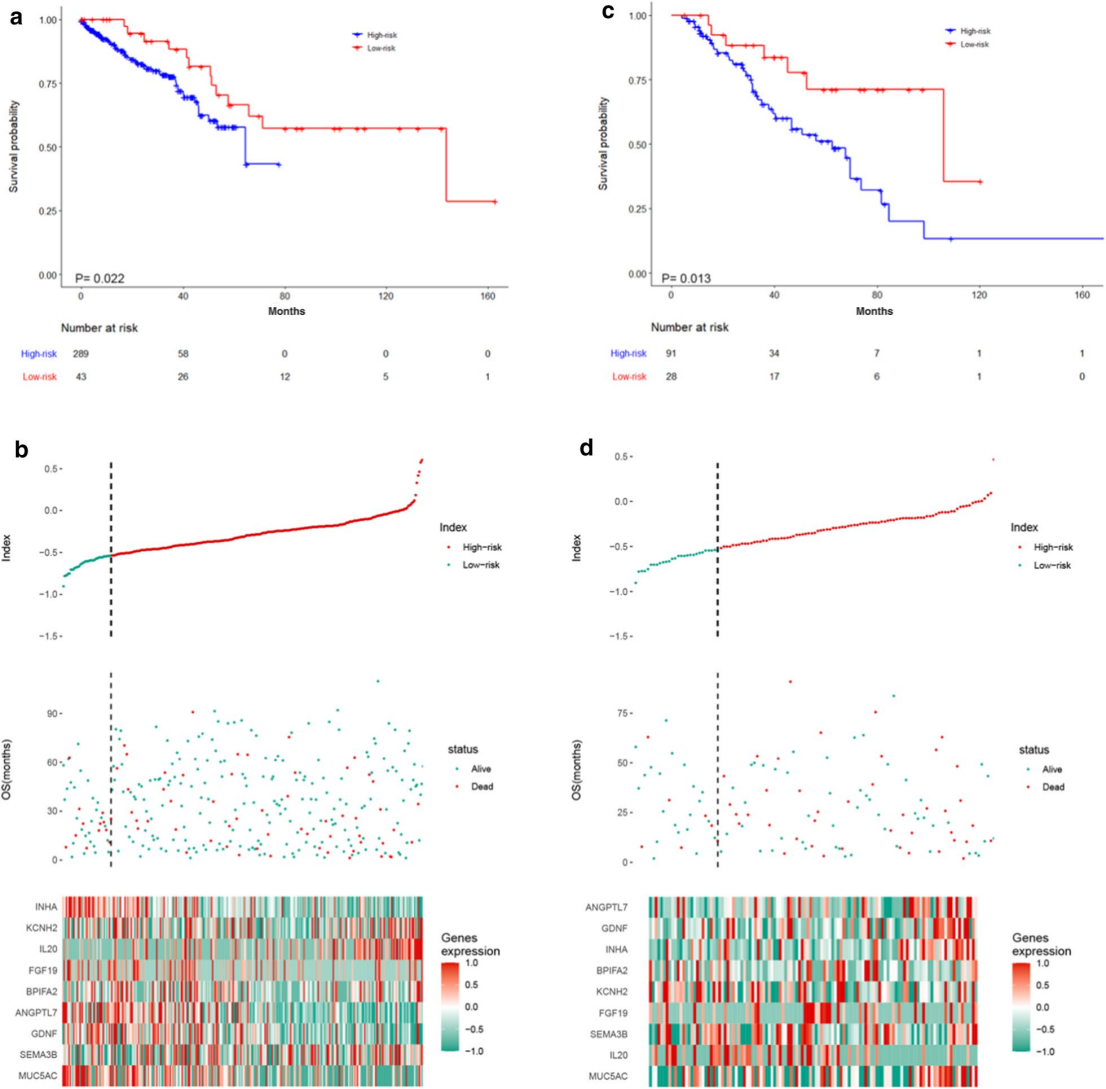
**a** The Kaplan–Meier survival analysis in the TCGA-KIRC cohort. **b** The risk score(upper), the OS (middle) and the expression of ten selected genes(bottom) in the TCGA-KIRC cohort. **c** The Kaplan–Meier survival analysis in the Jiangsu University Affiliated Wujin Hospital and the Affiliated Geriatric Hospital of Nanjing Medical University cohort. **d** The risk score(upper), the OS (middle) and the expression of ten selected genes(bottom) in the Jiangsu University Affiliated Wujin Hospital and the Affiliated Geriatric Hospital of Nanjing Medical University cohort

**Fig.4:**
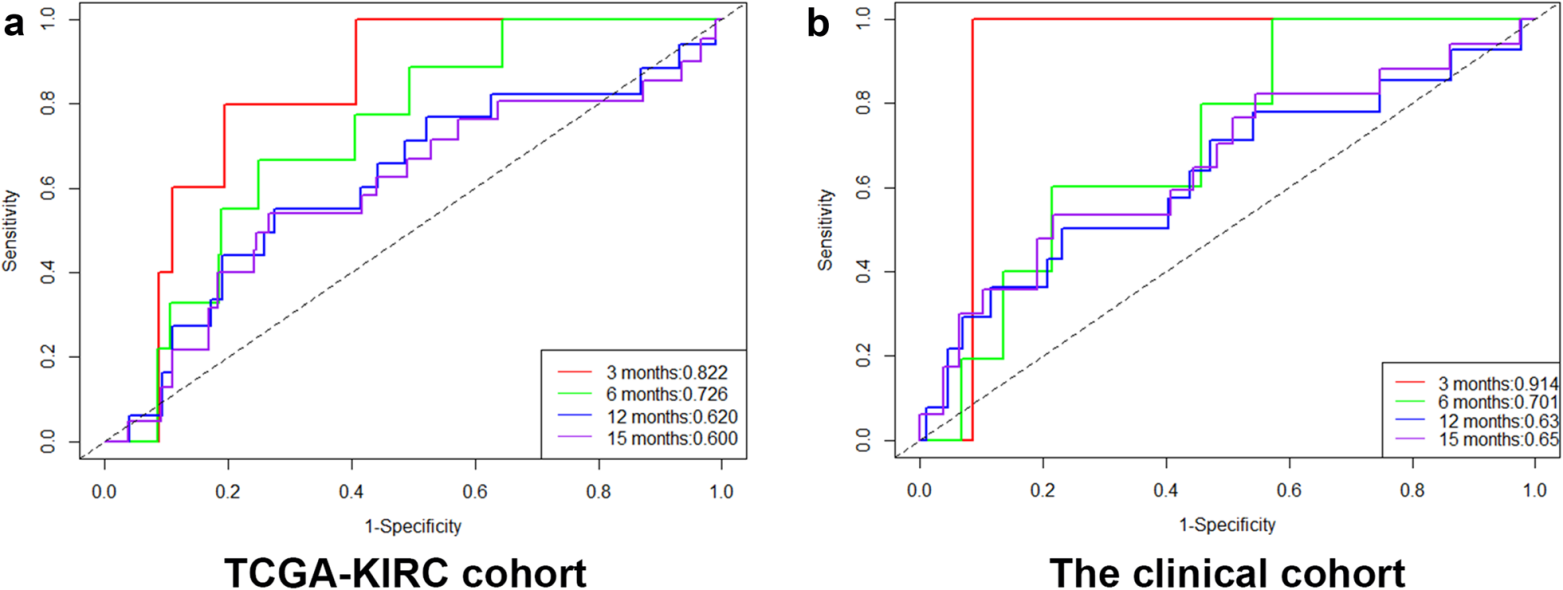
**a** The time-dependent ROC curves of TCGA-KIRC cohort. **b** The time-dependent ROC curves of the Jiangsu University Affiliated Wujin Hospital and the Affiliated Geriatric Hospital of Nanjing Medical University

Kaplan–Meier survival analysis of overall survival (OS) was conducted between the high-risk and low-risk groups in the TCGA-KIRC cohort, which demonstrated that patients with a high-risk score were correlated with worse outcomes (Fig. 3a). Figure 3b shows the distribution of risk score, survival information and ten-gene expression profiles of these two groups. To further evaluate the predictive ability of the immune-related risk score for prognosis, we conducted ROC curve analysis for OS (3 month AUC = 0.822, 6 month AUC = 0.726, 12 month AUC = 0.620 and 16 month AUC = 0.600, Fig. 4a). These analyses proved that the immune-related risk score revealed a promising prognostic ability for OS.

To determine the robustness of the immune-related risk score, the performance of the risk score was assessed in the clinical cohort, which consisted of 119 kidney renal clear cell carcinoma patients. Using the formula provided above and the same cut-off value obtained from the TCGA-KIRC cohort, the patients in our cohort were divided into high-risk and low-risk groups. The results showed that patients with a low risk had remarkably better OS than those with a high risk, which was consistent with the results from the TCGA-KIRC cohort (Fig. 3c). In addition, the distribution of the risk score, survival information and ten-gene expression profile in the Jiangsu University Affiliated Wujin Hospital and the Affiliated Geriatric Hospital of Nanjing Medical University cohort are shown in Fig. 3b. Furthermore, we conducted ROC curve analysis in the Jiangsu University Affiliated Wujin Hospital and the Affiliated Geriatric Hospital of Nanjing Medical University cohort, which demonstrated robust ability to predict patient prognosis (3 month AUC = 0.914, 6 month AUC = 0.701, 12 month AUC = 0.630 and 16 month AUC = 0.650, Fig. 4b). In summary, we showed that the immune-related risk score has predictive ability.

### Correlation between different immunophenotypes and immune-related risk score

In the previous analysis, we determined that 187 immune-related DEGs and ten genes are closely related to immune-related biological processes. Thus, we will further analyse the relationship between the immune-related risk score and immunophenotypes, including the immune cell infiltration status in the tumour microenvironment and immune checkpoint expression.

**Fig.5:**
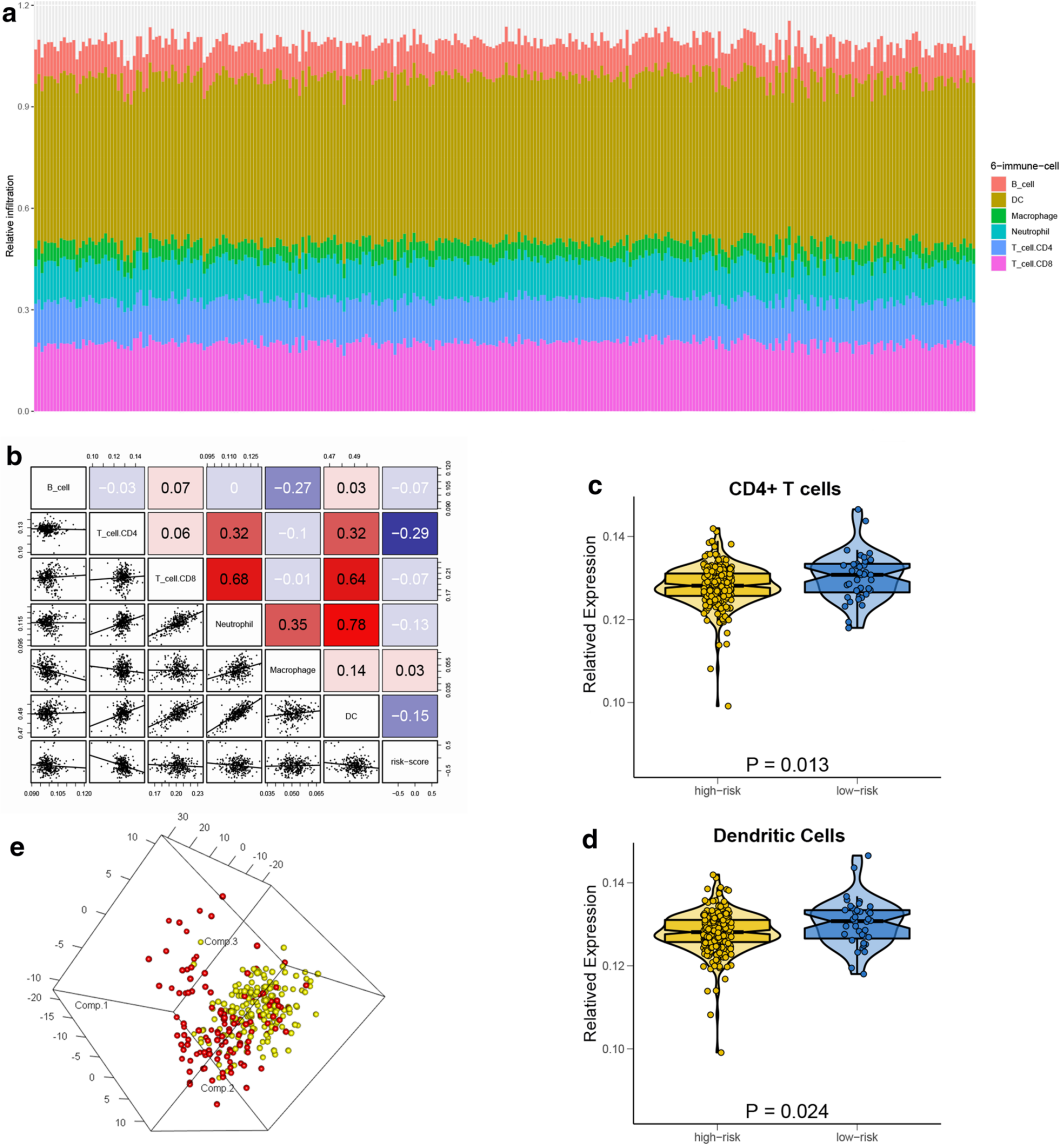
**a** The landscape of the immune infiltration in the TCGA-KIRC cohort using Timer online tools. **b** The correlation of the risk score and six mainly immune infiltration cell subpopulations. Box-Violin plots visualizing significantly different immune cells: **c** CD4 + T cells, **d** Dendritic cells

Using the TIMER website tool, we identified relationships between the immune-related risk score and 6 mainly infiltrating immune cells obtained from TCGA-KIRC cohort patients (Fig. 5a). In addition, the relative infiltration of different subpopulations of 6 immune infiltration cells and the risk score (Fig. 5b) were weakly to moderately correlated, especially in the relationship between risk score and CD4 + T cells or dendritic cells. The dot-box plot showed that the low-risk KIRC patients had a higher related infiltration of CD4 + T cells or dendritic cells than high-risk KIRC patients (Fig. 5c, d). Moreover, we separated high- and low-risk group patients into two discrete groups based on the related infiltration of six main immune cell subpopulations (Fig. 5e). In summary, we constructed a link between the immune-related risk score and the tumour microenvironment.

**Fig.6:**
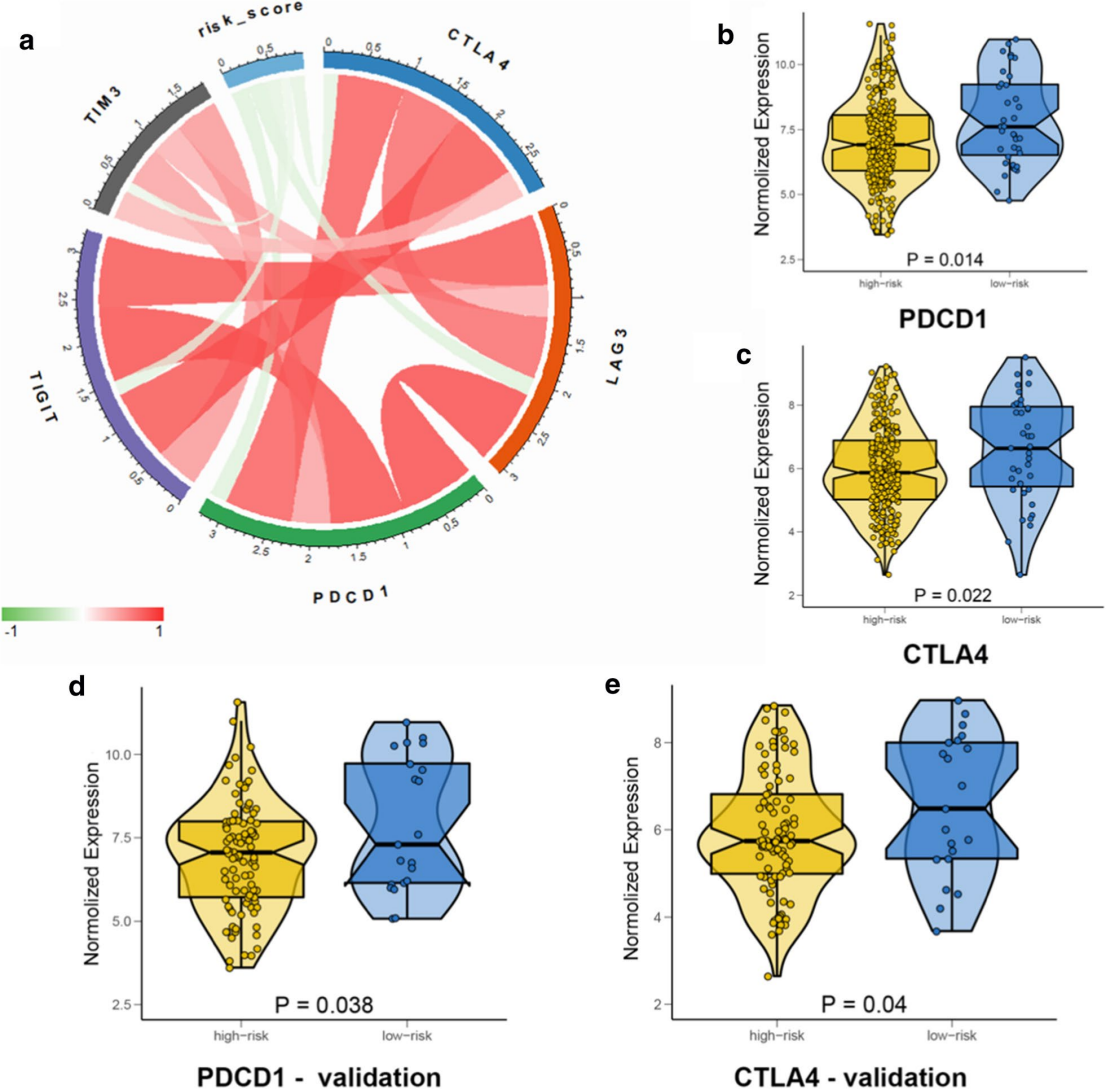
**a** The correlation of the risk score and several key immune checkpoints expression. Box-Violin plots visualizing significantly different immune checkpoint expression: **b** PDCD1 (encoding PD-1), **c** CTLA4

Immune checkpoint inhibitors have been shown to have antitumour roles by reversing tumour-evading immune surveillance. In clinical practice, high expression of immune checkpoints can be used as an indicator to predict the immune response after immunotherapy. Thus, it is meaningful to construct a link between the immune-related risk score and immune checkpoint expression of genes, namely, PDCD1 (encoding PD-1), LAG3, TIGIT, TIM-3 and CTLA4. We found that the risk score was negatively related to the expression of those immune checkpoint genes (Fig. 6a). In addition, the expression of PDCD1 and CTLA4 in the high-risk group was significantly higher than that in the low-risk group (Fig. 6b, c). To validate the expression differences in PDCD1 and CTLA4 between high-risk patients and low-risk patients, we detected PDCD1 and CTLA4 expression in Jiangsu University Affiliated Wujin Hospital and the Affiliated Geriatric Hospital of Nanjing Medical University cohort using qRT-PCR. Among this cohort of patients, high-risk patients expressed higher levels of PDCD1 and CTLA4 than low-risk patients (Fig. 6d, e). Therefore, we believe that there is a close connection between the immune-related risk score and expression of immune checkpoint genes.

## Discussion

In this study, we developed a new prognostic signature based on 10 immune-related genes for VHL-mutated ccRCC and validated it in an independent cohort from our centre. The prognostic immune signature performed well in classifying patients into subgroups with different survival outcomes. The genes integrated in the immune signature were mostly involved in GPCR ligand binding, chemotaxis, second messenger-mediated signalling, cytokine-cytokine receptor interaction, and vascular processes in the circulatory system. The correlations between hub genes and infiltrating immune cells, such as B cells, CD4 + T cells, CD8 + T cells, macrophages, neutrophils and dendritic cells, were analysed by using the deconvolution algorithm based on the TIMER database [13]. We found that the high-risk patients had lower infiltrating levels of CD4 + T cells and dendritic cells than low-risk patients, which validated and expanded the results that the heterogeneity of immune infiltration was vital for the progression of ccRCC. Based on the above findings, our signature might play a role in the prognosis differences observed between risk subgroups.

The immune‐related signature has been proposed in several cancers, including glioblastoma, pancreatic cancer, and renal carcinoma, which performed well in clinical validation [14–16]. However, these studies have several limitations, such as relatively small sample sizes and lack of independent validation. Even more importantly, these signatures ignored the correlation between key mutations and the response to immune therapy, which led to blurred and poor predictions. Based on previous studies and our analysis from the TCGA, VHL mutations are widely detected in ccRCC, which affects the VHL/HIF/VEGF pathway and plays an important role in angiogenesis [17]. However, the correlation between VHL status and PD-L1 expression has not been investigated much. Several studies have indicated that VHL mutations might activate effector T cells and increase cytokine levels in ccRCC [18]. As previous studies reported that VHL mutant tumor cells have stronger heterogeneity or tumor neoantigen levels, we performed further investigation on the hierarchical analysis (Additional file 4: Fig. S3 and Additional file 5: Fig. S4). However, a recent study showed opposite results: PD-L1 expression positively correlated with wild-type VHL ccRCC. The downstream pathways have already been reported to induce PD-L1 expression in constitutive immune responses [8]. The aim of the current study was to identify a VHL mutation-related signature that would stratify patients into different risk subgroups for further precise therapies.

Recently, ICI therapies have attracted increasing attention and made impressive prognoses in cancer patients, providing a prospective future for cancer treatment [19]. However, large-scale studies from clinical practice or ongoing trials with immunotherapies show limited effectiveness in some diagnostic patients. The tumour immune microenvironment has been considered a crucial issue in the effectiveness of immunotherapy. Thus, a better understanding of the tumour immune microenvironment might aid in improving the efficacy of current immunotherapies [20]. The correlations between hub genes and infiltrating immune cells, such as B cells, CD4 + T cells, CD8 + T cells, macrophages, neutrophils and dendritic cells, were analysed by using the deconvolution algorithm based on the TIMER database. We found that the high-risk patients had lower infiltrating levels of CD4 + T cells and dendritic cells, which validated and expanded the results that the heterogeneity of immune infiltration was vital for the progression of ccRCC. These results were consistent with those from published papers [14]. In previous reports on ccRCC, Pan et al. used the CIBERSORT database to conduct an overall analysis of the patient’s immune infiltration profile. They also found that CD4 + T cells and DCs have a high level of infiltration in well-differentiated ccRCC patients [21]. The immune microenvironment is constructed by tumour cells and tumour-infiltrating immune cells, which regulate the expression of immune checkpoints. The abovementioned results suggest that the poor prognosis of the high-risk group may be due to the immunosuppressive microenvironment in this subgroup, which promoted the progression of ccRCC [22]. In addition, our signature might help in deciding whether high-risk patients would benefit from immune checkpoint inhibitors.

The expression of PD-1 and CTLA4 is usually applied for predicting the immune response [23]. Therefore, we explored the association between key immune checkpoints (CTLA-4, PD-1, TIGIT, LAG3, and TIM-3) and the signature. The high-risk ccRCC patients had significantly lower expression of CTLA-4 and PD-1 than the low-risk patients, which was confirmed in the validation cohort. However, the opposite results were obtained in previous studies conducted in lower-grade glioma (LGG), which indicated a complex immune microenvironment in VHL-mutated ccRCC.

In GO analysis, we found that VHLmut had a significantly stronger local immunophenotype than VHLwt ccRCC. GPCR ligand binding, chemotaxis, cytokine-cytokine receptor interaction, and cellular response to growth factor stimulus were highly enriched in the low-risk groups. INHA, or inhibin subunit alpha, encodes a member of the TGF-beta (transforming growth factor-beta) superfamily of proteins and participates in the processing of the alpha subunit of the inhibin A and B protein complexes. INHA regulates numerous cellular processes, such as cell proliferation, apoptosis, immune response and hormone secretion [24, 25]. IL-20 is a cytokine structurally related to interleukin 10 (IL-10), which transduces its signal through signal transducer and activator of transcription 3 (STAT3) and mediates inflammation and the immune response [26]. HLA-DQA1 is an HLA class II alpha chain paralogue. It participates in the immune system by presenting peptides derived from exogenous proteins. Published studies have indicated that HLA-DQA1 is a feasible biomarker that plays an important role in ESCC progression and diagnosis, as well as a potential target for the treatment of patients with ESCC [27].

Our research provides new insights into the VHL-mutated ccRCC immune microenvironment and immune-related therapies. However, our research was a retrospective study, and the results need to be further confirmed by prospective studies. Moreover, functional and mechanistic studies should be conducted to support the clinical application.

## Conclusion

In summary, we developed and validated an immune-related signature constructed by 10 genes and determined the overall intensity of the immune response in the ccRCC microenvironment. This is the first study to identify an immune-related signature associated with VHL mutations. Notably, this study provided an immunological perspective to predict the clinical outcome of RCC.

## Supplementary Information

**Additional file 1: Table S1.** Primers sequence. **Table.S2.** The different expressed genes. **Table S3.** Immune-related DEGs. **Table S4.** The validation cohort patients' clinical information.

**Additional file 2: Figure S1.** **a** The Kaplan-Meier survival analysis in the TCGA-KIRC dataset between VHL mutation and wildtype patients. **b** The percentage of VHL status in KIRC patients with different pathological staging and **c** Fuhrman grading.

**Additional file 3: Figure S2.** The pathway enrichment of immune-related DGEs. The red rectangles indicate immune-related pathways.

**Additional file 4: Figure S3.** Tthe hierarchical analysis of VHL mutation.

**Additional file 5: Figure S4.** Survival analysis of immune related signature in VHL wt and WHL mutation subgroups.

## Data Availability

Yes.

## Abbreviations

ccRCC: Clear cell renal cell carcinoma
VHL: Von Hippel-Lindau
TME: Tumour microenvironment
