# Supplementary material for: Development and validation of a VHL-associated immune prognostic signature for clear cell renal cell carcinoma
Source: Cancer Cell Int. 2020 Dec 7;20:584. doi: 10.1186/s12935-020-01670-5 (PMC7720505; doi:10.1186/s12935-020-01670-5)
Supplement: Supplementary file 1 — Additional file 1: Table S1. Primers sequence. Table.S2. The different expressed genes. Table S3. Immune-related DEGs. Table S4. The validation cohort patients' clinical information. [file 12935_2020_1670_MOESM1_ESM.docx]

Table.S1 Primers Sequence

| SEMA3B - Sequence (5'->3') |  |
| --- | --- |
| Forward primer | AGACGTAAGGAATGGCGACC |
| Reverse primer | TACAAGAAAGTTGGGCACCAG |
| KCNH2 - Sequence (5'->3') |  |
| Forward primer | CATCGCGACGATCTCCTTGA |
| Reverse primer | GAATCATGTTCGTGTCGCGG |
| INHA - Sequence (5'->3') |  |
| Forward primer | TTCCACTACTGTCATGGTGGT |
| Reverse primer | AGTGCTGCGTGAGAAGGTTG |
| BPIFA2 - Sequence (5'->3') |  |
| Forward primer | CAATGACCTAAGCAATGTCGTGG |
| Reverse primer | TGCCAAGCACTGGATTTCTGA |
| FGF19 - Sequence (5'->3') |  |
| Forward primer | CGGAGGAAGACTGTGCTTTCG |
| Reverse primer | CTCGGATCGGTACACATTGTAG |
| IL20 - Sequence (5'->3') |  |
| Forward primer | ATGAAAGCCTCTAGTCTTGCCT |
| Reverse primer | GCCCCGTATCTCAGAAAATCC |
| GDNF - Sequence (5'->3') |  |
| Forward primer | GGCAGTGCTTCCTAGAAGAGA |
| Reverse primer | GGCAGTGCTTCCTAGAAGAGA |
| ANGPTL7 - Sequence (5'->3') |  |
| Forward primer | GCCCAAGTTGCCAACCTTAG |
| Reverse primer | AGGGAAGAGCAGTCGTAGATG |
| MUC5AC- Sequence (5'->3') |  |
| Forward primer | TGCCCCTACAACAAGAACAAC |
| Reverse primer | GGAACAGCACTGGGAGTAGTT |
| HLA-DQA1- Sequence (5'->3') |  |
| Forward primer | TCGCTCTGACCACCGTGAT |
| Reverse primer | AGGGACCGTAAAACTGGTACAA |
| PDCD1- Sequence (5'->3') |  |
| Forward primer | CCAGGATGGTTCTTAGACTCCC |
| Reverse primer | TTTAGCACGAAGCTCTCCGAT |
| CTLA4- Sequence (5'->3') |  |
| Forward primer | GCCCTGCACTCTCCTGTTTTT |
| Reverse primer | GGTTGCCGCACAGACTTCA |

Table.S2 The different expressed genes

| Gene_id | LogFC | P.Value | Adj.P.Val | Gene Name |
| --- | --- | --- | --- | --- |
| ENSG00000001626 | 1.342224455 | 8.43E-11 | 1.17E-08 | CFTR |
| ENSG00000004846 | 2.392805473 | 6.39E-19 | 5.05E-16 | ABCB5 |
| ENSG00000004939 | 2.197212066 | 3.91E-10 | 4.55E-08 | SLC4A1 |
| ENSG00000005001 | 1.648596487 | 1.10E-06 | 4.11E-05 | PRSS22 |
| ENSG00000005513 | 1.115171725 | 5.74E-12 | 1.06E-09 | SOX8 |
| ENSG00000006128 | 1.918228581 | 1.29E-07 | 6.90E-06 | TAC1 |
| ENSG00000006747 | 1.157660747 | 1.83E-09 | 1.73E-07 | SCIN |
| ENSG00000007216 | 1.057356417 | 0.000436501 | 0.005367637 | SLC13A2 |
| ENSG00000007306 | 1.294507557 | 0.002543233 | 0.020041177 | CEACAM7 |
| ENSG00000008196 | 1.066868814 | 0.006343796 | 0.038797065 | TFAP2B |
| ENSG00000009709 | 1.083341573 | 0.006798503 | 0.040683721 | PAX7 |
| ENSG00000010282 | 4.304208687 | 3.21E-31 | 1.13E-27 | HHATL |
| ENSG00000010438 | 1.247600637 | 1.83E-06 | 6.28E-05 | PRSS3 |
| ENSG00000011347 | 1.578130411 | 1.94E-11 | 3.18E-09 | SYT7 |
| ENSG00000013588 | 1.685941359 | 1.26E-13 | 3.60E-11 | GPRC5A |
| ENSG00000014257 | 1.145315249 | 1.86E-07 | 9.32E-06 | ACPP |
| ENSG00000016082 | 1.409779556 | 0.000156526 | 0.002400544 | ISL1 |
| ENSG00000016402 | 2.025797183 | 3.24E-14 | 1.08E-11 | IL20RA |
| ENSG00000035720 | 1.557769897 | 2.22E-11 | 3.60E-09 | STAP1 |
| ENSG00000041515 | 1.508144352 | 3.86E-15 | 1.57E-12 | MYO16 |
| ENSG00000042304 | 1.359109786 | 0.000867976 | 0.008961511 | C2orf83 |
| ENSG00000043591 | 1.08263735 | 2.68E-08 | 1.79E-06 | ADRB1 |
| ENSG00000046774 | 1.858909927 | 0.005177626 | 0.033509014 | MAGEC2 |
| ENSG00000047936 | 1.975451543 | 8.65E-09 | 6.80E-07 | ROS1 |
| ENSG00000049283 | 1.605318174 | 1.02E-07 | 5.62E-06 | EPN3 |
| ENSG00000055732 | 1.240280277 | 3.38E-08 | 2.16E-06 | MCOLN3 |
| ENSG00000056291 | 1.28648139 | 5.58E-06 | 0.000159356 | NPFFR2 |
| ENSG00000057149 | 1.124621187 | 0.008532245 | 0.04777662 | SERPINB3 |
| ENSG00000057593 | 1.017902981 | 2.66E-06 | 8.58E-05 | F7 |
| ENSG00000058085 | 1.153727267 | 1.30E-08 | 9.60E-07 | LAMC2 |
| ENSG00000062038 | 1.889559905 | 3.47E-16 | 1.68E-13 | CDH3 |
| ENSG00000064270 | 1.016733487 | 1.75E-07 | 8.87E-06 | ATP2C2 |
| ENSG00000065320 | 1.444573992 | 1.27E-19 | 1.14E-16 | NTN1 |
| ENSG00000065618 | 1.205772512 | 5.31E-09 | 4.39E-07 | COL17A1 |
| ENSG00000066032 | 2.416559839 | 1.36E-16 | 7.40E-14 | CTNNA2 |
| ENSG00000069011 | 1.357922689 | 1.69E-05 | 0.000397001 | PITX1 |
| ENSG00000069206 | 1.228166768 | 6.30E-05 | 0.001167028 | ADAM7 |
| ENSG00000070729 | 2.139820367 | 5.84E-19 | 4.71E-16 | CNGB1 |
| ENSG00000070915 | 2.638096626 | 3.97E-16 | 1.90E-13 | SLC12A3 |
| ENSG00000072041 | 2.319545091 | 1.91E-09 | 1.80E-07 | SLC6A15 |
| ENSG00000072954 | 1.469777886 | 4.19E-21 | 4.92E-18 | TMEM38A |
| ENSG00000073792 | 1.384501143 | 8.91E-12 | 1.59E-09 | IGF2BP2 |
| ENSG00000074803 | 1.416889724 | 4.52E-05 | 0.000891518 | SLC12A1 |
| ENSG00000075035 | 1.150127682 | 2.29E-07 | 1.12E-05 | WSCD2 |
| ENSG00000075673 | 1.321579816 | 0.000365125 | 0.004677565 | ATP12A |
| ENSG00000077009 | 4.477339955 | 6.29E-35 | 3.04E-31 | NMRK2 |
| ENSG00000078898 | 1.619327205 | 0.001441044 | 0.013142548 | BPIFB2 |
| ENSG00000079112 | 1.212937228 | 1.17E-09 | 1.17E-07 | CDH17 |
| ENSG00000079841 | 1.082159483 | 7.81E-07 | 3.11E-05 | RIMS1 |
| ENSG00000080031 | 1.226156756 | 3.55E-07 | 1.61E-05 | PTPRH |
| ENSG00000080511 | 2.506106845 | 3.62E-10 | 4.28E-08 | RDH8 |
| ENSG00000083307 | 1.320385378 | 0.000209013 | 0.0030255 | GRHL2 |
| ENSG00000083720 | 1.11561365 | 6.25E-14 | 1.95E-11 | OXCT1 |
| ENSG00000085741 | 1.055231926 | 3.57E-08 | 2.25E-06 | WNT11 |
| ENSG00000086159 | 3.460532894 | 1.04E-21 | 1.54E-18 | AQP6 |
| ENSG00000086506 | 1.122172212 | 3.85E-05 | 0.00077632 | HBQ1 |
| ENSG00000086548 | 1.277654718 | 5.36E-06 | 0.000154165 | CEACAM6 |
| ENSG00000087128 | 1.65185142 | 2.90E-07 | 1.37E-05 | TMPRSS11E |
| ENSG00000088726 | 1.096927423 | 1.12E-08 | 8.35E-07 | TMEM40 |
| ENSG00000088836 | 1.547042201 | 1.95E-16 | 9.95E-14 | SLC4A11 |
| ENSG00000089116 | 1.025638923 | 0.003403265 | 0.024783407 | LHX5 |
| ENSG00000089199 | 1.334208275 | 1.64E-07 | 8.43E-06 | CHGB |
| ENSG00000089356 | 1.704743319 | 6.17E-14 | 1.94E-11 | FXYD3 |
| ENSG00000091482 | 1.515410356 | 0.003344161 | 0.024463548 | SMPX |
| ENSG00000092068 | 1.15421122 | 1.51E-09 | 1.46E-07 | SLC7A8 |
| ENSG00000094755 | 1.387377676 | 1.54E-09 | 1.48E-07 | GABRP |
| ENSG00000095203 | 1.496980997 | 7.28E-09 | 5.83E-07 | EPB41L4B |
| ENSG00000095752 | 1.168484677 | 4.95E-07 | 2.15E-05 | IL11 |
| ENSG00000096088 | 1.755327872 | 1.21E-11 | 2.11E-09 | PGC |
| ENSG00000099399 | 3.595755247 | 3.59E-09 | 3.08E-07 | MAGEB2 |
| ENSG00000099869 | 1.941868491 | 1.58E-08 | 1.14E-06 | IGF2-AS |
| ENSG00000099937 | 1.819150006 | 1.26E-16 | 6.98E-14 | SERPIND1 |
| ENSG00000100290 | 1.389901493 | 4.01E-17 | 2.42E-14 | BIK |
| ENSG00000100341 | 1.177264787 | 0.003613168 | 0.025935947 | PNPLA5 |
| ENSG00000100362 | 3.047245953 | 4.84E-18 | 3.46E-15 | PVALB |
| ENSG00000100473 | 1.218882236 | 1.54E-08 | 1.11E-06 | COCH |
| ENSG00000100604 | 1.362446756 | 3.28E-08 | 2.11E-06 | CHGA |
| ENSG00000100625 | 1.580745826 | 2.15E-12 | 4.47E-10 | SIX4 |
| ENSG00000100665 | 1.347464023 | 3.26E-06 | 0.000101909 | SERPINA4 |
| ENSG00000100867 | 1.975924773 | 1.74E-17 | 1.16E-14 | DHRS2 |
| ENSG00000100884 | 1.254953357 | 1.67E-06 | 5.79E-05 | CPNE6 |
| ENSG00000101098 | 2.108897407 | 1.59E-13 | 4.42E-11 | RIMS4 |
| ENSG00000101144 | 2.073679029 | 1.66E-10 | 2.18E-08 | BMP7 |
| ENSG00000101210 | 1.325134429 | 6.79E-07 | 2.76E-05 | EEF1A2 |
| ENSG00000101323 | 1.235127092 | 0.002484077 | 0.019719656 | HAO1 |
| ENSG00000101349 | 1.502290995 | 0.000134899 | 0.002129536 | PAK5 |
| ENSG00000101443 | 1.254807622 | 2.93E-10 | 3.61E-08 | WFDC2 |
| ENSG00000101638 | 1.170033278 | 1.83E-08 | 1.29E-06 | ST8SIA5 |
| ENSG00000101850 | 1.386987537 | 3.76E-11 | 5.65E-09 | GPR143 |
| ENSG00000102021 | 1.630485713 | 0.005082854 | 0.033051955 | LUZP4 |
| ENSG00000102243 | 2.941068272 | 7.76E-09 | 6.15E-07 | VGLL1 |
| ENSG00000102678 | 1.722949698 | 1.85E-06 | 6.35E-05 | FGF9 |
| ENSG00000103089 | 1.797105847 | 1.14E-16 | 6.37E-14 | FA2H |
| ENSG00000103253 | 1.030921514 | 7.94E-08 | 4.52E-06 | HAGHL |
| ENSG00000103546 | 2.018293923 | 2.84E-09 | 2.52E-07 | SLC6A2 |
| ENSG00000104112 | 1.825487883 | 4.84E-20 | 4.46E-17 | SCG3 |
| ENSG00000104332 | 1.340749439 | 9.26E-07 | 3.56E-05 | SFRP1 |
| ENSG00000104413 | 1.525575531 | 5.93E-06 | 0.000168197 | ESRP1 |
| ENSG00000104755 | 1.119712865 | 0.002479473 | 0.019695233 | ADAM2 |
| ENSG00000105141 | 5.694288712 | 3.65E-25 | 7.43E-22 | CASP14 |
| ENSG00000105143 | 2.683269911 | 2.07E-17 | 1.36E-14 | SLC1A6 |
| ENSG00000105219 | 1.570195181 | 4.73E-13 | 1.17E-10 | CNTD2 |
| ENSG00000105289 | 1.610624752 | 1.00E-13 | 2.98E-11 | TJP3 |
| ENSG00000105388 | 1.241771807 | 0.000241824 | 0.003402932 | CEACAM5 |
| ENSG00000105523 | 1.121700037 | 1.74E-05 | 0.000406738 | FAM83E |
| ENSG00000105737 | 1.157089576 | 1.99E-06 | 6.73E-05 | GRIK5 |
| ENSG00000105929 | 2.524101963 | 8.04E-11 | 1.12E-08 | ATP6V0A4 |
| ENSG00000106302 | 1.211729594 | 7.40E-06 | 0.000200303 | HYAL4 |
| ENSG00000106541 | 3.062349785 | 9.68E-16 | 4.40E-13 | AGR2 |
| ENSG00000106809 | 1.063358219 | 3.56E-05 | 0.000729768 | OGN |
| ENSG00000107165 | 1.542406875 | 1.67E-05 | 0.000393714 | TYRP1 |
| ENSG00000107295 | 1.181066566 | 0.000120405 | 0.001957303 | SH3GL2 |
| ENSG00000107447 | 2.521049158 | 2.72E-12 | 5.47E-10 | DNTT |
| ENSG00000108244 | 2.307249745 | 1.98E-20 | 2.01E-17 | KRT23 |
| ENSG00000108852 | 1.033145807 | 3.96E-10 | 4.60E-08 | MPP2 |
| ENSG00000109182 | 1.51408411 | 8.88E-07 | 3.46E-05 | CWH43 |
| ENSG00000109205 | 1.698456265 | 0.004183163 | 0.028731668 | ODAM |
| ENSG00000109684 | 1.346703985 | 5.05E-10 | 5.66E-08 | CLNK |
| ENSG00000109851 | 1.309324482 | 0.002863027 | 0.021848943 | DBX1 |
| ENSG00000110080 | 1.027919298 | 2.32E-15 | 9.87E-13 | ST3GAL4 |
| ENSG00000110203 | 1.539399981 | 3.40E-08 | 2.16E-06 | FOLR3 |
| ENSG00000110245 | 1.107746405 | 0.000779494 | 0.008286893 | APOC3 |
| ENSG00000110484 | 1.401084531 | 0.000516435 | 0.006095097 | SCGB2A2 |
| ENSG00000110680 | 5.127747745 | 1.20E-39 | 2.32E-35 | CALCA |
| ENSG00000110881 | 1.050425358 | 2.39E-09 | 2.18E-07 | ASIC1 |
| ENSG00000110975 | 1.597223522 | 7.20E-06 | 0.000196496 | SYT10 |
| ENSG00000111218 | 1.652099915 | 2.84E-13 | 7.37E-11 | PRMT8 |
| ENSG00000111319 | 1.518586397 | 1.93E-10 | 2.51E-08 | SCNN1A |
| ENSG00000112530 | 1.67190015 | 1.95E-16 | 9.95E-14 | PACRG |
| ENSG00000113073 | 1.999157796 | 5.65E-11 | 8.21E-09 | SLC4A9 |
| ENSG00000113327 | 1.17039711 | 0.008313769 | 0.046967266 | GABRG2 |
| ENSG00000113578 | 1.053966005 | 1.30E-07 | 6.95E-06 | FGF1 |
| ENSG00000113805 | 1.8424825 | 8.96E-11 | 1.24E-08 | CNTN3 |
| ENSG00000113889 | 2.901337516 | 7.92E-14 | 2.41E-11 | KNG1 |
| ENSG00000113905 | 3.293700197 | 1.87E-19 | 1.65E-16 | HRG |
| ENSG00000113946 | 1.512556857 | 1.77E-07 | 8.94E-06 | CLDN16 |
| ENSG00000114200 | 1.111449365 | 1.08E-06 | 4.03E-05 | BCHE |
| ENSG00000114854 | 1.51727631 | 1.20E-12 | 2.58E-10 | TNNC1 |
| ENSG00000115112 | 1.445088925 | 1.61E-07 | 8.30E-06 | TFCP2L1 |
| ENSG00000115263 | 1.658536396 | 0.0037101 | 0.026357794 | GCG |
| ENSG00000115457 | 1.071917495 | 1.25E-09 | 1.23E-07 | IGFBP2 |
| ENSG00000115616 | 1.614328634 | 1.00E-07 | 5.56E-06 | SLC9A2 |
| ENSG00000116039 | 3.031161044 | 2.26E-23 | 3.79E-20 | ATP6V1B1 |
| ENSG00000116194 | 1.322437349 | 8.48E-10 | 8.88E-08 | ANGPTL1 |
| ENSG00000116218 | 1.227013872 | 0.008247569 | 0.046710055 | NPHS2 |
| ENSG00000116254 | 1.264465077 | 8.84E-09 | 6.88E-07 | CHD5 |
| ENSG00000116661 | 1.313377583 | 3.13E-11 | 4.83E-09 | FBXO2 |
| ENSG00000116690 | 1.330784077 | 3.77E-13 | 9.52E-11 | PRG4 |
| ENSG00000117322 | 1.442833256 | 3.02E-07 | 1.42E-05 | CR2 |
| ENSG00000118194 | 2.340904461 | 2.01E-21 | 2.78E-18 | TNNT2 |
| ENSG00000118271 | 3.765920381 | 4.94E-27 | 1.27E-23 | TTR |
| ENSG00000118434 | 1.213431195 | 0.005877438 | 0.036752755 | SPACA1 |
| ENSG00000118596 | 1.07429037 | 4.09E-09 | 3.46E-07 | SLC16A7 |
| ENSG00000119698 | 1.004582034 | 6.35E-06 | 0.000177342 | PPP4R4 |
| ENSG00000119715 | 1.015328508 | 7.02E-06 | 0.000192534 | ESRRB |
| ENSG00000119913 | 1.077014754 | 0.002839087 | 0.021704786 | TECTB |
| ENSG00000120094 | 1.328240477 | 0.0003217 | 0.004253385 | HOXB1 |
| ENSG00000120251 | 1.495673437 | 3.07E-06 | 9.70E-05 | GRIA2 |
| ENSG00000122584 | 1.243786796 | 0.005835767 | 0.036539771 | NXPH1 |
| ENSG00000122711 | 1.608198053 | 1.35E-07 | 7.18E-06 | SPINK4 |
| ENSG00000123572 | 1.404511954 | 5.62E-07 | 2.39E-05 | NRK |
| ENSG00000123836 | 1.315315944 | 7.66E-17 | 4.35E-14 | PFKFB2 |
| ENSG00000123838 | 1.022799144 | 0.000337113 | 0.004406278 | C4BPA |
| ENSG00000123843 | 2.208248408 | 8.61E-25 | 1.59E-21 | C4BPB |
| ENSG00000123999 | 1.733742824 | 2.07E-15 | 8.89E-13 | INHA |
| ENSG00000124102 | 1.339844924 | 1.58E-06 | 5.52E-05 | PI3 |
| ENSG00000124107 | 2.346188432 | 2.78E-15 | 1.17E-12 | SLPI |
| ENSG00000124134 | 1.233890706 | 9.22E-05 | 0.001584284 | KCNS1 |
| ENSG00000124143 | 2.357917928 | 9.05E-13 | 2.07E-10 | ARHGAP40 |
| ENSG00000124249 | 1.978838289 | 1.82E-15 | 8.01E-13 | KCNK15 |
| ENSG00000124467 | 1.880761882 | 0.000106072 | 0.001769822 | PSG8 |
| ENSG00000124939 | 1.426578692 | 2.85E-08 | 1.89E-06 | SCGB2A1 |
| ENSG00000125355 | 1.439098051 | 6.43E-13 | 1.54E-10 | TMEM255A |
| ENSG00000125820 | 1.324850709 | 1.74E-05 | 0.000407657 | NKX2-2 |
| ENSG00000125850 | 1.40596627 | 4.90E-05 | 0.000950199 | OVOL2 |
| ENSG00000125965 | 1.295693555 | 1.67E-09 | 1.59E-07 | GDF5 |
| ENSG00000126460 | 1.428325227 | 1.42E-15 | 6.32E-13 | PRRG2 |
| ENSG00000126500 | 1.17454923 | 3.68E-08 | 2.31E-06 | FLRT1 |
| ENSG00000126856 | 1.005180778 | 6.60E-05 | 0.001212808 | PRDM7 |
| ENSG00000126861 | 1.453771965 | 2.91E-15 | 1.21E-12 | OMG |
| ENSG00000126950 | 1.682542703 | 5.72E-15 | 2.23E-12 | TMEM35A |
| ENSG00000127561 | 2.187130974 | 6.17E-28 | 1.70E-24 | SYNGR3 |
| ENSG00000128045 | 1.075758691 | 1.02E-05 | 0.000261786 | RASL11B |
| ENSG00000128422 | 2.806691664 | 4.13E-35 | 2.28E-31 | KRT17 |
| ENSG00000128918 | 1.385361008 | 1.18E-10 | 1.61E-08 | ALDH1A2 |
| ENSG00000129988 | -1.285130361 | 2.22E-05 | 0.000498422 | LBP |
| ENSG00000129991 | 1.412530496 | 3.48E-05 | 0.000716941 | TNNI3 |
| ENSG00000130054 | 1.194362806 | 7.08E-10 | 7.58E-08 | FAM155B |
| ENSG00000130222 | 1.08599889 | 4.38E-13 | 1.09E-10 | GADD45G |
| ENSG00000130226 | 1.079106113 | 1.97E-05 | 0.000451585 | DPP6 |
| ENSG00000130294 | 1.635137799 | 2.49E-10 | 3.11E-08 | KIF1A |
| ENSG00000130600 | 1.498675782 | 9.99E-13 | 2.23E-10 | H19 |
| ENSG00000130701 | 1.544782907 | 0.000938077 | 0.009494225 | RBBP8NL |
| ENSG00000130829 | 2.159532086 | 4.54E-12 | 8.70E-10 | DUSP9 |
| ENSG00000131096 | 1.308986608 | 9.91E-13 | 2.23E-10 | PYY |
| ENSG00000131183 | 1.22281034 | 2.59E-05 | 0.000562817 | SLC34A1 |
| ENSG00000131721 | 2.645943024 | 9.70E-07 | 3.69E-05 | RHOXF2 |
| ENSG00000131730 | 1.504930986 | 7.65E-10 | 8.08E-08 | CKMT2 |
| ENSG00000131737 | 1.156343647 | 0.000459172 | 0.005581058 | KRT34 |
| ENSG00000131746 | 1.0253746 | 6.69E-07 | 2.74E-05 | TNS4 |
| ENSG00000131771 | 1.873585605 | 4.69E-11 | 6.94E-09 | PPP1R1B |
| ENSG00000131910 | 2.786781966 | 3.99E-10 | 4.62E-08 | NR0B2 |
| ENSG00000132026 | 1.270232269 | 4.41E-06 | 0.000131676 | RTBDN |
| ENSG00000132429 | 1.532413033 | 1.07E-12 | 2.34E-10 | POPDC3 |
| ENSG00000132563 | 1.373557105 | 1.36E-13 | 3.87E-11 | REEP2 |
| ENSG00000132677 | 3.19095759 | 1.82E-16 | 9.49E-14 | RHBG |
| ENSG00000132693 | -1.363776347 | 8.43E-06 | 0.000223668 | CRP |
| ENSG00000132698 | 1.946728393 | 1.26E-08 | 9.35E-07 | RAB25 |
| ENSG00000132746 | 1.166872502 | 0.000267582 | 0.0036747 | ALDH3B2 |
| ENSG00000132874 | 2.988118438 | 9.95E-36 | 6.41E-32 | SLC14A2 |
| ENSG00000133878 | 1.166758002 | 8.06E-08 | 4.58E-06 | DUSP26 |
| ENSG00000134115 | 1.195325286 | 1.45E-07 | 7.65E-06 | CNTN6 |
| ENSG00000134121 | 1.112469879 | 1.95E-06 | 6.63E-05 | CHL1 |
| ENSG00000134193 | 1.404741232 | 1.33E-07 | 7.06E-06 | REG4 |
| ENSG00000134258 | 2.049115984 | 2.25E-11 | 3.62E-09 | VTCN1 |
| ENSG00000134363 | 1.215982431 | 3.66E-09 | 3.13E-07 | FST |
| ENSG00000134398 | 1.21767385 | 9.77E-07 | 3.71E-05 | ERN2 |
| ENSG00000134640 | 1.251120247 | 0.00810372 | 0.046070771 | MTNR1B |
| ENSG00000134827 | 1.316583503 | 4.81E-06 | 0.000141599 | TCN1 |
| ENSG00000135063 | 1.062878743 | 4.11E-13 | 1.03E-10 | FAM189A2 |
| ENSG00000135116 | 2.247003983 | 3.03E-21 | 3.79E-18 | HRK |
| ENSG00000135226 | 1.064997289 | 0.000177822 | 0.002663606 | UGT2B28 |
| ENSG00000135324 | 1.013595537 | 2.08E-05 | 0.00047184 | MRAP2 |
| ENSG00000135346 | 2.662645517 | 3.68E-12 | 7.26E-10 | CGA |
| ENSG00000135373 | 1.543535396 | 2.70E-07 | 1.29E-05 | EHF |
| ENSG00000135374 | 2.156984894 | 2.09E-10 | 2.68E-08 | ELF5 |
| ENSG00000135480 | 2.293491445 | 1.24E-20 | 1.33E-17 | KRT7 |
| ENSG00000135824 | 1.481763678 | 1.40E-10 | 1.86E-08 | RGS8 |
| ENSG00000135903 | 1.61271091 | 3.85E-06 | 0.000117675 | PAX3 |
| ENSG00000136110 | 3.034356134 | 1.96E-28 | 5.83E-25 | CNMD |
| ENSG00000136155 | 3.054851999 | 6.27E-25 | 1.21E-21 | SCEL |
| ENSG00000136235 | 1.314862231 | 8.54E-15 | 3.09E-12 | GPNMB |
| ENSG00000136352 | 1.258332796 | 0.005265652 | 0.033909331 | NKX2-1 |
| ENSG00000136574 | 1.525128074 | 1.06E-05 | 0.000269352 | GATA4 |
| ENSG00000136944 | 2.552168367 | 4.58E-16 | 2.13E-13 | LMX1B |
| ENSG00000137077 | 1.451375757 | 1.54E-07 | 7.99E-06 | CCL21 |
| ENSG00000137142 | 1.406491307 | 4.35E-07 | 1.93E-05 | IGFBPL1 |
| ENSG00000137198 | 1.072763555 | 7.64E-12 | 1.38E-09 | GMPR |
| ENSG00000137203 | 1.257386672 | 8.32E-08 | 4.70E-06 | TFAP2A |
| ENSG00000137440 | 3.179575523 | 1.65E-13 | 4.55E-11 | FGFBP1 |
| ENSG00000137561 | 1.049014216 | 4.84E-05 | 0.000939733 | TTPA |
| ENSG00000137648 | 2.043728822 | 3.17E-11 | 4.87E-09 | TMPRSS4 |
| ENSG00000137766 | 1.751872227 | 1.15E-12 | 2.48E-10 | UNC13C |
| ENSG00000137843 | 1.031242304 | 0.000257262 | 0.003564983 | PAK6 |
| ENSG00000137868 | 1.419816878 | 5.49E-10 | 6.10E-08 | STRA6 |
| ENSG00000137878 | 1.221676208 | 5.86E-08 | 3.49E-06 | GCOM1 |
| ENSG00000138039 | 2.013278712 | 7.35E-08 | 4.25E-06 | LHCGR |
| ENSG00000138271 | 2.78913229 | 1.20E-13 | 3.47E-11 | GPR87 |
| ENSG00000138472 | 1.09478078 | 0.007071338 | 0.041837399 | GUCA1C |
| ENSG00000138678 | 1.056044619 | 6.70E-09 | 5.40E-07 | GPAT3 |
| ENSG00000138696 | 1.359389222 | 3.22E-06 | 0.000100776 | BMPR1B |
| ENSG00000138798 | 1.264313746 | 5.08E-07 | 2.19E-05 | EGF |
| ENSG00000138813 | 1.149986761 | 0.001288551 | 0.012084385 | C4orf17 |
| ENSG00000139044 | 1.215165005 | 5.10E-11 | 7.47E-09 | B4GALNT3 |
| ENSG00000139144 | 3.961270345 | 6.16E-29 | 1.99E-25 | PIK3C2G |
| ENSG00000139304 | 1.505549666 | 1.57E-07 | 8.13E-06 | PTPRQ |
| ENSG00000139515 | 1.077143155 | 0.008453922 | 0.047504251 | PDX1 |
| ENSG00000139780 | 1.972470292 | 3.16E-07 | 1.47E-05 | METTL21C |
| ENSG00000139865 | 1.291563912 | 3.36E-06 | 0.000104363 | TTC6 |
| ENSG00000139973 | 1.026817745 | 4.86E-06 | 0.000142376 | SYT16 |
| ENSG00000140279 | 1.008153458 | 1.71E-07 | 8.74E-06 | DUOX2 |
| ENSG00000140459 | 1.553492234 | 5.50E-07 | 2.35E-05 | CYP11A1 |
| ENSG00000140465 | 2.011752232 | 3.19E-08 | 2.06E-06 | CYP1A1 |
| ENSG00000140481 | 1.643382185 | 4.67E-08 | 2.82E-06 | CCDC33 |
| ENSG00000140505 | 1.649565389 | 3.78E-07 | 1.71E-05 | CYP1A2 |
| ENSG00000140519 | 4.465382937 | 5.67E-34 | 2.19E-30 | RHCG |
| ENSG00000140600 | 1.094770269 | 2.03E-05 | 0.000463202 | SH3GL3 |
| ENSG00000141639 | 1.466200858 | 8.70E-06 | 0.000230197 | MAPK4 |
| ENSG00000141744 | 1.267484601 | 3.33E-07 | 1.54E-05 | PNMT |
| ENSG00000141750 | 1.527461735 | 9.04E-07 | 3.51E-05 | STAC2 |
| ENSG00000142449 | 1.481848135 | 5.72E-07 | 2.43E-05 | FBN3 |
| ENSG00000142515 | 2.49254901 | 2.80E-11 | 4.40E-09 | KLK3 |
| ENSG00000142549 | -1.047688845 | 0.000531155 | 0.00620841 | IGLON5 |
| ENSG00000142675 | 1.068269865 | 6.19E-07 | 2.58E-05 | CNKSR1 |
| ENSG00000143001 | 2.13591355 | 2.74E-09 | 2.45E-07 | TMEM61 |
| ENSG00000143320 | 1.34003 | 1.15E-11 | 2.01E-09 | CRABP2 |
| ENSG00000143355 | 4.523781205 | 3.91E-36 | 3.02E-32 | LHX9 |
| ENSG00000143387 | 1.256081728 | 7.46E-15 | 2.80E-12 | CTSK |
| ENSG00000143469 | 3.406574656 | 7.44E-21 | 8.22E-18 | SYT14 |
| ENSG00000143556 | 1.27668847 | 0.000208331 | 0.003018887 | S100A7 |
| ENSG00000143768 | 1.123766197 | 1.48E-06 | 5.21E-05 | LEFTY2 |
| ENSG00000143882 | 1.153733732 | 4.08E-10 | 4.71E-08 | ATP6V1C2 |
| ENSG00000144227 | 2.69330604 | 1.18E-06 | 4.34E-05 | NXPH2 |
| ENSG00000144481 | 1.014732554 | 1.97E-07 | 9.79E-06 | TRPM8 |
| ENSG00000144644 | 1.229445062 | 3.80E-06 | 0.000116251 | GADL1 |
| ENSG00000144834 | 1.185571566 | 1.39E-05 | 0.00033765 | TAGLN3 |
| ENSG00000145087 | 1.727414363 | 1.23E-11 | 2.13E-09 | STXBP5L |
| ENSG00000145107 | 1.009558116 | 1.72E-07 | 8.75E-06 | TM4SF19 |
| ENSG00000145192 | 2.505899766 | 3.39E-12 | 6.73E-10 | AHSG |
| ENSG00000145242 | 1.335577386 | 0.000164218 | 0.002495595 | EPHA5 |
| ENSG00000145808 | 1.623150641 | 4.96E-05 | 0.000959639 | ADAMTS19 |
| ENSG00000146047 | -1.015460601 | 0.000284231 | 0.003850596 | H2BC1 |
| ENSG00000146267 | 1.650849985 | 7.21E-11 | 1.01E-08 | FAXC |
| ENSG00000146411 | 1.134808974 | 4.65E-07 | 2.03E-05 | SLC2A12 |
| ENSG00000146755 | 1.566766178 | 3.09E-12 | 6.18E-10 | TRIM50 |
| ENSG00000147041 | 1.229504931 | 9.70E-09 | 7.42E-07 | SYTL5 |
| ENSG00000147246 | 1.237883684 | 0.000823924 | 0.00859395 | HTR2C |
| ENSG00000147256 | 1.308322164 | 0.000545253 | 0.006331426 | ARHGAP36 |
| ENSG00000147257 | 1.066410391 | 2.30E-06 | 7.61E-05 | GPC3 |
| ENSG00000147481 | 1.37492563 | 0.000156478 | 0.002400544 | SNTG1 |
| ENSG00000147571 | 2.385046352 | 4.26E-11 | 6.35E-09 | CRH |
| ENSG00000147606 | 2.789879711 | 2.86E-21 | 3.68E-18 | SLC26A7 |
| ENSG00000147613 | 1.328069934 | 0.008635255 | 0.048218781 | PSKH2 |
| ENSG00000147614 | 2.23152792 | 2.95E-13 | 7.58E-11 | ATP6V0D2 |
| ENSG00000147689 | 2.156546399 | 9.35E-14 | 2.80E-11 | FAM83A |
| ENSG00000147697 | 1.218223794 | 3.50E-07 | 1.60E-05 | GSDMC |
| ENSG00000148123 | 1.083830527 | 0.000946247 | 0.009547888 | PLPPR1 |
| ENSG00000148346 | 2.470156704 | 1.29E-21 | 1.85E-18 | LCN2 |
| ENSG00000148513 | 1.335851915 | 1.59E-06 | 5.54E-05 | ANKRD30A |
| ENSG00000148735 | 1.162996289 | 1.82E-06 | 6.25E-05 | PLEKHS1 |
| ENSG00000148795 | 2.212067597 | 2.74E-21 | 3.65E-18 | CYP17A1 |
| ENSG00000149043 | 1.52183214 | 5.16E-07 | 2.22E-05 | SYT8 |
| ENSG00000149305 | 1.324164295 | 0.004369992 | 0.029656752 | HTR3B |
| ENSG00000149575 | 1.380163544 | 8.71E-08 | 4.91E-06 | SCN2B |
| ENSG00000149599 | 1.555121432 | 2.24E-16 | 1.12E-13 | DUSP15 |
| ENSG00000149948 | 1.912049559 | 9.09E-13 | 2.07E-10 | HMGA2 |
| ENSG00000149970 | 1.062343112 | 8.49E-11 | 1.18E-08 | CNKSR2 |
| ENSG00000150201 | 3.061490367 | 6.41E-13 | 1.54E-10 | FXYD4 |
| ENSG00000150556 | 1.866523154 | 8.90E-10 | 9.28E-08 | LYPD6B |
| ENSG00000150750 | 2.744806757 | 1.25E-09 | 1.23E-07 | C11orf53 |
| ENSG00000151365 | 2.526708577 | 2.01E-19 | 1.72E-16 | THRSP |
| ENSG00000151418 | 4.055173252 | 2.77E-09 | 2.47E-07 | ATP6V1G3 |
| ENSG00000151704 | 1.502058752 | 1.04E-05 | 0.000265295 | KCNJ1 |
| ENSG00000151834 | 1.931581857 | 1.43E-05 | 0.00034453 | GABRA2 |
| ENSG00000152669 | 1.104409495 | 2.38E-06 | 7.85E-05 | CCNO |
| ENSG00000152822 | 1.312982419 | 1.84E-10 | 2.41E-08 | GRM1 |
| ENSG00000152931 | 2.775711555 | 1.25E-15 | 5.62E-13 | PART1 |
| ENSG00000153253 | 1.155089188 | 2.81E-06 | 8.98E-05 | SCN3A |
| ENSG00000153292 | 1.55055636 | 1.17E-05 | 0.000292346 | ADGRF1 |
| ENSG00000153446 | 1.803561337 | 6.08E-14 | 1.94E-11 | C16orf89 |
| ENSG00000153820 | 1.405735625 | 3.02E-05 | 0.000639953 | SPHKAP |
| ENSG00000154162 | 1.138302966 | 9.01E-06 | 0.000237428 | CDH12 |
| ENSG00000154438 | 1.368047638 | 6.57E-05 | 0.001209393 | ASZ1 |
| ENSG00000154764 | 1.433804457 | 1.30E-07 | 6.95E-06 | WNT7A |
| ENSG00000154975 | 1.510597265 | 0.000105185 | 0.001762626 | CA10 |
| ENSG00000155066 | 1.06624791 | 0.000111682 | 0.001842759 | PROM2 |
| ENSG00000155495 | 1.55656449 | 0.000171558 | 0.002584805 | MAGEC1 |
| ENSG00000155511 | 1.453661481 | 4.52E-10 | 5.16E-08 | GRIA1 |
| ENSG00000155816 | 1.331475535 | 4.34E-07 | 1.92E-05 | FMN2 |
| ENSG00000155886 | 1.645407021 | 3.58E-07 | 1.62E-05 | SLC24A2 |
| ENSG00000155918 | 1.476144795 | 3.22E-09 | 2.80E-07 | RAET1L |
| ENSG00000156284 | 4.159709833 | 1.39E-14 | 4.99E-12 | CLDN8 |
| ENSG00000157064 | -1.127836872 | 9.16E-10 | 9.39E-08 | NMNAT2 |
| ENSG00000157404 | 1.006562382 | 3.35E-10 | 4.03E-08 | KIT |
| ENSG00000157613 | 1.245198083 | 9.89E-11 | 1.36E-08 | CREB3L1 |
| ENSG00000157703 | 1.273577453 | 6.28E-06 | 0.000175976 | SVOPL |
| ENSG00000157851 | 1.429607826 | 4.06E-07 | 1.81E-05 | DPYSL5 |
| ENSG00000158022 | 3.503376875 | 6.17E-42 | 2.39E-37 | TRIM63 |
| ENSG00000158055 | 1.257146948 | 6.37E-11 | 9.19E-09 | GRHL3 |
| ENSG00000158816 | 1.443902266 | 6.25E-08 | 3.69E-06 | VWA5B1 |
| ENSG00000158874 | 1.519611286 | 1.40E-06 | 4.97E-05 | APOA2 |
| ENSG00000159184 | 1.171969341 | 0.000808696 | 0.008481869 | HOXB13 |
| ENSG00000159248 | 2.760360553 | 5.66E-09 | 4.65E-07 | GJD2 |
| ENSG00000159263 | 1.210601597 | 4.27E-10 | 4.90E-08 | SIM2 |
| ENSG00000159337 | -1.250175393 | 4.86E-06 | 0.000142432 | PLA2G4D |
| ENSG00000159495 | 1.656260324 | 7.77E-05 | 0.00138401 | TGM7 |
| ENSG00000159763 | 2.788039301 | 3.01E-17 | 1.91E-14 | PIP |
| ENSG00000160181 | -1.403913196 | 1.15E-05 | 0.000288803 | TFF2 |
| ENSG00000160182 | 1.631545787 | 2.50E-05 | 0.000548731 | TFF1 |
| ENSG00000160200 | 1.478239084 | 1.06E-12 | 2.34E-10 | CBS |
| ENSG00000160951 | 1.841730577 | 6.56E-10 | 7.18E-08 | PTGER1 |
| ENSG00000160963 | 2.010252103 | 4.69E-12 | 8.94E-10 | COL26A1 |
| ENSG00000161267 | 1.177005131 | 3.01E-11 | 4.66E-09 | BDH1 |
| ENSG00000161281 | 1.089339434 | 1.28E-12 | 2.75E-10 | COX7A1 |
| ENSG00000161798 | 1.693213042 | 3.48E-09 | 3.01E-07 | AQP5 |
| ENSG00000161992 | 3.711971538 | 4.10E-13 | 1.03E-10 | PRR35 |
| ENSG00000162009 | 1.283682433 | 2.14E-05 | 0.000481553 | SSTR5 |
| ENSG00000162040 | 1.130648595 | 0.00427571 | 0.02921974 | HS3ST6 |
| ENSG00000162344 | 1.663142624 | 5.29E-05 | 0.001012913 | FGF19 |
| ENSG00000162399 | 1.845114531 | 0.000487715 | 0.005842126 | BSND |
| ENSG00000162493 | 1.086946448 | 7.80E-09 | 6.17E-07 | PDPN |
| ENSG00000162552 | 1.024534739 | 1.70E-07 | 8.70E-06 | WNT4 |
| ENSG00000162624 | -1.357160824 | 7.63E-05 | 0.001366173 | LHX8 |
| ENSG00000162631 | 1.170184731 | 0.000274899 | 0.003752909 | NTNG1 |
| ENSG00000162763 | 2.551676854 | 1.09E-06 | 4.06E-05 | LRRC52 |
| ENSG00000162891 | 1.518359 | 5.16E-06 | 0.000149628 | IL20 |
| ENSG00000162951 | 2.219847813 | 3.96E-09 | 3.36E-07 | LRRTM1 |
| ENSG00000163331 | 2.680057927 | 3.68E-21 | 4.45E-18 | DAPL1 |
| ENSG00000163394 | 2.233813957 | 3.44E-07 | 1.58E-05 | CCKAR |
| ENSG00000163499 | 1.747718183 | 0.000136778 | 0.002155679 | CRYBA2 |
| ENSG00000163530 | 1.00147081 | 0.004517704 | 0.030376691 | DPPA2 |
| ENSG00000163586 | 1.025382512 | 0.000396452 | 0.004973463 | FABP1 |
| ENSG00000163623 | 1.786969479 | 3.09E-09 | 2.71E-07 | NKX6-1 |
| ENSG00000163631 | 1.834182246 | 9.39E-13 | 2.12E-10 | ALB |
| ENSG00000163833 | 1.047736984 | 2.23E-06 | 7.41E-05 | FBXO40 |
| ENSG00000163975 | 1.173011042 | 2.45E-08 | 1.67E-06 | MELTF |
| ENSG00000164007 | 1.350658244 | 5.24E-06 | 0.000151717 | CLDN19 |
| ENSG00000164076 | 1.072774048 | 0.000440186 | 0.005399499 | CAMKV |
| ENSG00000164188 | 2.046040219 | 4.46E-16 | 2.11E-13 | RANBP3L |
| ENSG00000164265 | -1.020876885 | 0.000375065 | 0.00477643 | SCGB3A2 |
| ENSG00000164363 | -1.309722958 | 4.44E-06 | 0.000132487 | SLC6A18 |
| ENSG00000164825 | 1.619988041 | 5.61E-14 | 1.81E-11 | DEFB1 |
| ENSG00000164972 | 1.33866876 | 1.87E-15 | 8.14E-13 | C9orf24 |
| ENSG00000165125 | 1.084385777 | 1.97E-05 | 0.000451585 | TRPV6 |
| ENSG00000165181 | 1.338469752 | 1.37E-11 | 2.35E-09 | SHOC1 |
| ENSG00000165186 | 1.034412623 | 7.41E-06 | 0.000200615 | PTCHD1 |
| ENSG00000165379 | 1.225526596 | 9.03E-09 | 7.02E-07 | LRFN5 |
| ENSG00000165553 | 1.330551237 | 7.18E-05 | 0.001302771 | NGB |
| ENSG00000165566 | 1.055602457 | 0.000277704 | 0.003780524 | AMER2 |
| ENSG00000165583 | 1.293963095 | 0.000509431 | 0.006027153 | SSX5 |
| ENSG00000165621 | 1.641635724 | 5.45E-09 | 4.51E-07 | OXGR1 |
| ENSG00000165643 | 1.305994373 | 0.000633255 | 0.007101827 | SOHLH1 |
| ENSG00000165685 | 2.546729058 | 1.30E-22 | 2.09E-19 | TMEM52B |
| ENSG00000165816 | 1.053683795 | 3.11E-06 | 9.82E-05 | VWA2 |
| ENSG00000165887 | 1.354124779 | 3.77E-08 | 2.35E-06 | ANKRD2 |
| ENSG00000165973 | 1.536976553 | 0.000224151 | 0.003203719 | NELL1 |
| ENSG00000166049 | 1.715142141 | 0.002327286 | 0.018846341 | PASD1 |
| ENSG00000166104 | 1.246031714 | 6.13E-05 | 0.001145291 | NA |
| ENSG00000166165 | 1.165402312 | 2.17E-13 | 5.74E-11 | CKB |
| ENSG00000166220 | 1.507564051 | 2.65E-08 | 1.78E-06 | TBATA |
| ENSG00000166558 | 2.417881414 | 5.30E-12 | 9.86E-10 | SLC38A8 |
| ENSG00000166736 | 1.298879839 | 2.62E-07 | 1.25E-05 | HTR3A |
| ENSG00000166828 | 1.270772819 | 0.000654853 | 0.007278197 | SCNN1G |
| ENSG00000166869 | 1.019695599 | 0.000200256 | 0.002919379 | CHP2 |
| ENSG00000166923 | 1.340240828 | 2.48E-07 | 1.20E-05 | GREM1 |
| ENSG00000166959 | 1.441869372 | 1.17E-05 | 0.000292054 | MS4A8 |
| ENSG00000167080 | 1.156817271 | 1.97E-05 | 0.00045215 | B4GALNT2 |
| ENSG00000167183 | 1.482101154 | 5.04E-09 | 4.19E-07 | PRR15L |
| ENSG00000167244 | 1.769948876 | 1.64E-14 | 5.76E-12 | IGF2 |
| ENSG00000167346 | 2.196134921 | 1.54E-06 | 5.40E-05 | MMP26 |
| ENSG00000167549 | 1.091976324 | 5.90E-09 | 4.82E-07 | CORO6 |
| ENSG00000167580 | 3.396003901 | 3.99E-12 | 7.84E-10 | AQP2 |
| ENSG00000167608 | 1.201582505 | 3.25E-10 | 3.92E-08 | TMC4 |
| ENSG00000167653 | 1.35490848 | 4.82E-10 | 5.44E-08 | PSCA |
| ENSG00000167656 | 1.12730948 | 0.001585447 | 0.01410924 | LY6D |
| ENSG00000167741 | 1.302905198 | 0.000106067 | 0.001769822 | GGT6 |
| ENSG00000167748 | 4.632125314 | 5.58E-38 | 7.20E-34 | KLK1 |
| ENSG00000167749 | 4.905682774 | 3.60E-37 | 3.48E-33 | KLK4 |
| ENSG00000167751 | 1.417150347 | 8.87E-05 | 0.001532146 | KLK2 |
| ENSG00000167754 | 1.567097339 | 0.000161194 | 0.0024593 | KLK5 |
| ENSG00000167755 | 1.735904675 | 9.41E-05 | 0.001610546 | KLK6 |
| ENSG00000167798 | 2.656043384 | 1.12E-13 | 3.27E-11 | C3P1 |
| ENSG00000167916 | 1.12817669 | 0.000769185 | 0.008215687 | KRT24 |
| ENSG00000168004 | 1.341871817 | 1.88E-11 | 3.10E-09 | PLAAT5 |
| ENSG00000168269 | 2.575586511 | 2.32E-06 | 7.68E-05 | FOXI1 |
| ENSG00000168333 | -2.118765264 | 5.69E-09 | 4.66E-07 | PPDPFL |
| ENSG00000168447 | 1.383370839 | 2.15E-06 | 7.22E-05 | SCNN1B |
| ENSG00000168481 | 1.45365898 | 3.21E-11 | 4.91E-09 | LGI3 |
| ENSG00000168509 | 1.387034853 | 1.35E-09 | 1.32E-07 | HJV |
| ENSG00000168621 | 1.189901841 | 7.18E-07 | 2.90E-05 | GDNF |
| ENSG00000168703 | 2.062611776 | 9.41E-09 | 7.25E-07 | WFDC12 |
| ENSG00000168785 | 1.150746604 | 4.13E-15 | 1.66E-12 | TSPAN5 |
| ENSG00000168830 | 1.737786282 | 0.000119059 | 0.001939654 | HTR1E |
| ENSG00000168878 | 3.68728355 | 3.21E-34 | 1.38E-30 | SFTPB |
| ENSG00000168907 | 2.626232312 | 2.15E-09 | 1.99E-07 | PLA2G4F |
| ENSG00000169006 | 1.19704107 | 0.001900841 | 0.016079817 | NTSR2 |
| ENSG00000169035 | 1.997997548 | 3.50E-07 | 1.60E-05 | KLK7 |
| ENSG00000169126 | 1.006045795 | 6.77E-06 | 0.000186352 | ARMC4 |
| ENSG00000169174 | 1.092684182 | 2.83E-05 | 0.000608585 | PCSK9 |
| ENSG00000169213 | 1.172400093 | 4.77E-06 | 0.000140711 | RAB3B |
| ENSG00000169218 | 1.069667157 | 0.000820548 | 0.008571739 | RSPO1 |
| ENSG00000169271 | 1.001274636 | 0.000564524 | 0.006506728 | HSPB3 |
| ENSG00000169297 | 1.897997534 | 5.19E-05 | 0.000995945 | NR0B1 |
| ENSG00000169344 | 2.433862668 | 1.17E-07 | 6.36E-06 | UMOD |
| ENSG00000169347 | 2.100906068 | 1.85E-06 | 6.34E-05 | GP2 |
| ENSG00000169393 | 1.464902551 | 0.000768376 | 0.008211584 | ELSPBP1 |
| ENSG00000169548 | 1.802953124 | 1.47E-06 | 5.19E-05 | ZNF280A |
| ENSG00000169550 | 1.339353354 | 0.004215759 | 0.028914473 | MUC15 |
| ENSG00000169594 | 2.573553182 | 1.41E-22 | 2.18E-19 | BNC1 |
| ENSG00000170382 | 1.070930484 | 8.56E-07 | 3.35E-05 | LRRN2 |
| ENSG00000170419 | 1.97636673 | 7.34E-09 | 5.87E-07 | VSTM2A |
| ENSG00000170477 | 1.665299934 | 2.49E-06 | 8.12E-05 | KRT4 |
| ENSG00000170523 | 1.010066523 | 0.000327005 | 0.004309115 | KRT83 |
| ENSG00000170549 | 1.413983538 | 3.54E-09 | 3.05E-07 | IRX1 |
| ENSG00000170561 | 1.14150335 | 3.40E-06 | 0.00010545 | IRX2 |
| ENSG00000170579 | 1.218383158 | 5.38E-10 | 5.99E-08 | DLGAP1 |
| ENSG00000170608 | 1.660330817 | 8.53E-10 | 8.91E-08 | FOXA3 |
| ENSG00000170689 | 1.394174654 | 9.30E-10 | 9.47E-08 | HOXB9 |
| ENSG00000170790 | -1.176029414 | 0.000288708 | 0.003894687 | OR10A2 |
| ENSG00000170835 | 1.510204049 | 5.03E-13 | 1.23E-10 | CEL |
| ENSG00000171004 | 2.000915721 | 5.42E-13 | 1.31E-10 | HS6ST2 |
| ENSG00000171126 | 1.945389175 | 8.34E-10 | 8.79E-08 | KCNG3 |
| ENSG00000171136 | 1.104456223 | 0.00025203 | 0.00350883 | RLN3 |
| ENSG00000171346 | 1.071398114 | 6.57E-05 | 0.001209393 | KRT15 |
| ENSG00000171401 | 1.853497577 | 9.18E-10 | 9.39E-08 | KRT13 |
| ENSG00000171403 | 1.238141326 | 0.001533753 | 0.013769599 | KRT9 |
| ENSG00000171431 | 2.639419577 | 6.32E-15 | 2.42E-12 | KRT20 |
| ENSG00000171505 | 1.08699034 | 0.001480566 | 0.013416679 | OR1N1 |
| ENSG00000171557 | -1.105739032 | 0.002799434 | 0.021473804 | FGG |
| ENSG00000171724 | 1.270384861 | 1.92E-08 | 1.34E-06 | VAT1L |
| ENSG00000171819 | 1.410394738 | 6.30E-07 | 2.62E-05 | ANGPTL7 |
| ENSG00000172005 | 1.464593469 | 1.15E-09 | 1.15E-07 | MAL |
| ENSG00000172023 | -1.224729097 | 0.000246459 | 0.003451136 | REG1B |
| ENSG00000172497 | 1.432686006 | 5.26E-06 | 0.000151908 | ACOT12 |
| ENSG00000172548 | 1.455859404 | 9.31E-10 | 9.47E-08 | NIPAL4 |
| ENSG00000172551 | 1.077266656 | 0.001771318 | 0.015267998 | MUCL1 |
| ENSG00000172799 | 1.247065776 | 3.31E-06 | 0.000103236 | NA |
| ENSG00000173253 | 2.532958488 | 7.50E-10 | 7.99E-08 | DMRT2 |
| ENSG00000173467 | 1.984339057 | 9.08E-06 | 0.000238996 | AGR3 |
| ENSG00000173612 | 3.18142007 | 1.53E-07 | 7.97E-06 | GPRC6A |
| ENSG00000173641 | 1.274480752 | 8.81E-09 | 6.87E-07 | HSPB7 |
| ENSG00000173714 | 1.153663487 | 2.32E-06 | 7.69E-05 | WFIKKN2 |
| ENSG00000173805 | 1.200119588 | 2.37E-09 | 2.17E-07 | HAP1 |
| ENSG00000173838 | 2.034988926 | 1.54E-16 | 8.26E-14 | MARCHF10 |
| ENSG00000173898 | 1.489473411 | 5.99E-08 | 3.56E-06 | SPTBN2 |
| ENSG00000174326 | 1.211607793 | 3.38E-11 | 5.13E-09 | SLC16A11 |
| ENSG00000174469 | 1.052298361 | 3.19E-09 | 2.79E-07 | CNTNAP2 |
| ENSG00000174473 | 1.172481359 | 2.42E-09 | 2.20E-07 | GALNTL6 |
| ENSG00000174514 | 1.155285748 | 5.22E-13 | 1.27E-10 | MFSD4A |
| ENSG00000174562 | 3.794604409 | 4.29E-12 | 8.34E-10 | KLK15 |
| ENSG00000174669 | 1.008025241 | 1.68E-11 | 2.84E-09 | SLC29A2 |
| ENSG00000175093 | 1.677095803 | 5.63E-15 | 2.22E-12 | SPSB4 |
| ENSG00000175229 | 1.598042028 | 1.96E-07 | 9.77E-06 | GAL3ST3 |
| ENSG00000175315 | 1.937134108 | 3.90E-20 | 3.77E-17 | CST6 |
| ENSG00000175329 | 1.208366669 | 0.002914319 | 0.022139891 | ISX |
| ENSG00000175497 | 1.13561818 | 0.000657653 | 0.007294423 | DPP10 |
| ENSG00000175793 | 1.345001785 | 4.37E-10 | 5.01E-08 | SFN |
| ENSG00000175832 | 1.637194518 | 9.92E-19 | 7.67E-16 | ETV4 |
| ENSG00000175906 | 1.305701055 | 6.51E-13 | 1.54E-10 | ARL4D |
| ENSG00000175928 | 1.104173646 | 4.16E-07 | 1.86E-05 | LRRN1 |
| ENSG00000176040 | 2.552750564 | 1.66E-13 | 4.56E-11 | TMPRSS7 |
| ENSG00000176387 | 1.236774314 | 2.69E-09 | 2.41E-07 | HSD11B2 |
| ENSG00000176406 | 1.503425152 | 4.58E-08 | 2.78E-06 | RIMS2 |
| ENSG00000176490 | 1.950338909 | 3.39E-14 | 1.11E-11 | DIRAS1 |
| ENSG00000176532 | 1.484944134 | 2.83E-11 | 4.41E-09 | PRR15 |
| ENSG00000176753 | 1.26215891 | 2.23E-06 | 7.43E-05 | C15orf56 |
| ENSG00000176884 | 1.003687608 | 1.01E-06 | 3.81E-05 | GRIN1 |
| ENSG00000176945 | 1.126361552 | 6.78E-10 | 7.32E-08 | MUC20 |
| ENSG00000177133 | 1.86549731 | 7.48E-10 | 7.99E-08 | PRDM16-DT |
| ENSG00000177191 | 1.206672084 | 1.29E-17 | 8.72E-15 | B3GNT8 |
| ENSG00000177234 | 1.463426105 | 1.25E-06 | 4.54E-05 | LINC01561 |
| ENSG00000177354 | 1.673329862 | 0.000139862 | 0.002195134 | C10orf71 |
| ENSG00000177511 | 1.063052217 | 6.83E-05 | 0.001249982 | ST8SIA3 |
| ENSG00000177807 | 2.232830661 | 3.47E-17 | 2.16E-14 | KCNJ10 |
| ENSG00000177938 | 1.335337676 | 0.003571487 | 0.025713113 | CAPZA3 |
| ENSG00000178015 | 1.002184762 | 4.55E-09 | 3.81E-07 | GPR150 |
| ENSG00000178171 | 1.203302447 | 7.64E-05 | 0.001367422 | AMER3 |
| ENSG00000178395 | 1.280140057 | 0.001862729 | 0.015852322 | CCDC185 |
| ENSG00000178568 | 1.063063396 | 0.000194102 | 0.002852244 | ERBB4 |
| ENSG00000178591 | 1.72909515 | 0.005936756 | 0.037027894 | DEFB125 |
| ENSG00000178645 | 1.735104489 | 2.13E-05 | 0.000480976 | C10orf53 |
| ENSG00000178752 | 1.024251159 | 1.43E-08 | 1.05E-06 | ERFE |
| ENSG00000178919 | 1.345509222 | 5.76E-05 | 0.001089126 | FOXE1 |
| ENSG00000179008 | 1.225731367 | 1.58E-05 | 0.000376081 | C14orf39 |
| ENSG00000179073 | 1.030831483 | 0.00753426 | 0.043830694 | TAAR3P |
| ENSG00000179141 | 1.021763802 | 0.000266989 | 0.003670839 | NA |
| ENSG00000179213 | 1.288061207 | 6.97E-05 | 0.001272997 | SIGLECL1 |
| ENSG00000179270 | 2.362365968 | 1.92E-09 | 1.80E-07 | PCARE |
| ENSG00000179399 | 1.243538924 | 2.77E-06 | 8.87E-05 | GPC5 |
| ENSG00000179914 | 1.049813884 | 2.60E-06 | 8.43E-05 | ITLN1 |
| ENSG00000180016 | 1.487236916 | 0.001125668 | 0.010894977 | OR1E1 |
| ENSG00000180176 | 1.333761108 | 1.34E-05 | 0.000326898 | TH |
| ENSG00000180251 | 3.69875483 | 3.42E-20 | 3.39E-17 | SLC9A4 |
| ENSG00000180772 | 1.407090163 | 0.003290949 | 0.024207066 | AGTR2 |
| ENSG00000180777 | 1.558540953 | 1.58E-07 | 8.15E-06 | ANKRD30B |
| ENSG00000180869 | 1.131060948 | 0.000288556 | 0.003894687 | LINC01555 |
| ENSG00000181092 | 1.291366876 | 0.006216692 | 0.038249568 | ADIPOQ |
| ENSG00000181392 | 1.564509378 | 1.12E-13 | 3.27E-11 | SYNE4 |
| ENSG00000181408 | 1.536664124 | 1.42E-05 | 0.000342478 | UTS2R |
| ENSG00000181418 | 1.18546769 | 1.86E-07 | 9.31E-06 | DDN |
| ENSG00000182035 | 1.458267162 | 9.36E-06 | 0.000245461 | ADIG |
| ENSG00000182048 | -1.217420266 | 7.70E-07 | 3.07E-05 | TRPC2 |
| ENSG00000182107 | 1.327952484 | 1.30E-09 | 1.28E-07 | TMEM30B |
| ENSG00000182132 | 1.985983116 | 7.93E-15 | 2.95E-12 | KCNIP1 |
| ENSG00000182255 | 1.002103409 | 0.001159169 | 0.011132855 | KCNA4 |
| ENSG00000182352 | 1.374772776 | 0.000220828 | 0.003164408 | C17orf77 |
| ENSG00000182687 | 1.069391928 | 1.88E-08 | 1.32E-06 | GALR2 |
| ENSG00000182759 | 1.41745624 | 9.94E-09 | 7.58E-07 | MAFA |
| ENSG00000182795 | 1.212756691 | 1.30E-06 | 4.67E-05 | C1orf116 |
| ENSG00000183036 | 2.357234153 | 1.19E-11 | 2.07E-09 | PCP4 |
| ENSG00000183185 | 1.196155878 | 0.000438859 | 0.005384934 | GABRR3 |
| ENSG00000183196 | 1.290236992 | 7.05E-10 | 7.57E-08 | CHST6 |
| ENSG00000183248 | 1.125622944 | 3.75E-08 | 2.34E-06 | PRR36 |
| ENSG00000183269 | 1.346408748 | 0.004784463 | 0.031636842 | OR52E8 |
| ENSG00000183287 | 1.440418513 | 2.72E-08 | 1.82E-06 | CCBE1 |
| ENSG00000183304 | -1.442027021 | 3.23E-06 | 0.000101155 | FAM9A |
| ENSG00000183571 | 1.528615105 | 1.82E-11 | 3.03E-09 | PGPEP1L |
| ENSG00000183578 | 1.637379016 | 1.29E-20 | 1.34E-17 | TNFAIP8L3 |
| ENSG00000183654 | 2.324125041 | 4.09E-06 | 0.000123905 | MARCHF11 |
| ENSG00000183668 | 3.657914867 | 5.72E-17 | 3.35E-14 | PSG9 |
| ENSG00000183844 | 1.464271579 | 1.95E-08 | 1.36E-06 | FAM3B |
| ENSG00000183888 | 2.154720732 | 6.93E-11 | 9.82E-09 | SRARP |
| ENSG00000183971 | 1.425188715 | 2.01E-12 | 4.21E-10 | NPW |
| ENSG00000184012 | 1.596060873 | 6.46E-07 | 2.67E-05 | TMPRSS2 |
| ENSG00000184058 | 1.19277592 | 1.24E-08 | 9.24E-07 | TBX1 |
| ENSG00000184292 | 1.410713539 | 2.41E-08 | 1.65E-06 | TACSTD2 |
| ENSG00000184363 | 1.616653066 | 1.10E-09 | 1.10E-07 | PKP3 |
| ENSG00000184414 | 1.098423142 | 6.26E-06 | 0.000175575 | NA |
| ENSG00000184524 | 1.429765173 | 2.31E-13 | 6.08E-11 | CEND1 |
| ENSG00000184672 | 1.638109221 | 0.000137282 | 0.002161852 | RALYL |
| ENSG00000184908 | 2.722512641 | 3.05E-16 | 1.49E-13 | CLCNKB |
| ENSG00000185133 | 2.422837483 | 8.28E-26 | 1.78E-22 | INPP5J |
| ENSG00000185149 | 1.361403087 | 0.001485749 | 0.013448903 | NPY2R |
| ENSG00000185156 | 1.474997651 | 1.43E-08 | 1.05E-06 | MFSD6L |
| ENSG00000185269 | 1.64291216 | 4.78E-11 | 7.04E-09 | NOTUM |
| ENSG00000185274 | 1.043599718 | 0.000447982 | 0.005472573 | GALNT17 |
| ENSG00000185290 | 2.187417371 | 3.66E-07 | 1.66E-05 | NUPR2 |
| ENSG00000185352 | 1.723601746 | 2.24E-11 | 3.61E-09 | HS6ST3 |
| ENSG00000185479 | 1.720408446 | 0.00016242 | 0.002472009 | KRT6B |
| ENSG00000185518 | 1.352074516 | 1.69E-11 | 2.84E-09 | SV2B |
| ENSG00000185565 | 1.929997283 | 2.19E-12 | 4.53E-10 | LSAMP |
| ENSG00000185742 | 1.022285297 | 1.11E-05 | 0.000280221 | C11orf87 |
| ENSG00000185818 | 1.53445083 | 3.77E-08 | 2.35E-06 | NAT8L |
| ENSG00000185847 | 1.129265525 | 0.00654104 | 0.039568289 | LINC01405 |
| ENSG00000186009 | 1.179799278 | 8.42E-07 | 3.30E-05 | ATP4B |
| ENSG00000186081 | 1.007093783 | 0.000230852 | 0.003281299 | KRT5 |
| ENSG00000186090 | 1.140223327 | 0.002992764 | 0.022571789 | HTR3D |
| ENSG00000186458 | 2.937545897 | 5.97E-07 | 2.52E-05 | DEFB132 |
| ENSG00000186466 | 1.024061054 | 6.58E-06 | 0.000182087 | NA |
| ENSG00000186510 | 2.63587653 | 2.27E-19 | 1.91E-16 | CLCNKA |
| ENSG00000186684 | 1.172663528 | 6.79E-11 | 9.70E-09 | CYP27C1 |
| ENSG00000186766 | 1.598693987 | 7.53E-10 | 8.00E-08 | FOXI2 |
| ENSG00000186832 | 1.258473836 | 4.69E-05 | 0.000915926 | KRT16 |
| ENSG00000186838 | 1.113965563 | 0.000805576 | 0.00846183 | SELENOV |
| ENSG00000186847 | 1.300021122 | 7.63E-06 | 0.00020562 | KRT14 |
| ENSG00000187123 | 1.379202893 | 6.54E-11 | 9.40E-09 | LYPD6 |
| ENSG00000187486 | 1.445622615 | 3.52E-17 | 2.16E-14 | KCNJ11 |
| ENSG00000187715 | 2.057430874 | 8.30E-13 | 1.92E-10 | KBTBD12 |
| ENSG00000187772 | 2.271914065 | 5.24E-05 | 0.001004277 | LIN28B |
| ENSG00000187848 | 1.02440167 | 0.000880737 | 0.009057 | P2RX2 |
| ENSG00000187889 | 1.07293514 | 2.83E-06 | 9.04E-05 | FYB2 |
| ENSG00000187922 | 1.074573031 | 6.42E-06 | 0.000178803 | LCN10 |
| ENSG00000188064 | 1.503877697 | 2.04E-09 | 1.91E-07 | WNT7B |
| ENSG00000188133 | 1.308527536 | 0.002219698 | 0.018161424 | TMEM215 |
| ENSG00000188175 | 2.564964402 | 2.41E-10 | 3.03E-08 | HEPACAM2 |
| ENSG00000188293 | 1.128564895 | 0.00161789 | 0.014305788 | IGFL1 |
| ENSG00000188338 | 2.214600082 | 2.32E-18 | 1.73E-15 | SLC38A3 |
| ENSG00000188488 | 1.494084674 | 4.47E-07 | 1.97E-05 | SERPINA5 |
| ENSG00000188512 | 1.012880699 | 1.14E-05 | 0.000286798 | NA |
| ENSG00000188581 | 2.162228481 | 4.93E-07 | 2.14E-05 | KRTAP1-1 |
| ENSG00000188668 | 1.422622745 | 0.004889811 | 0.032123695 | NA |
| ENSG00000188883 | 1.371678086 | 1.21E-06 | 4.44E-05 | KLRG2 |
| ENSG00000188910 | 1.937615019 | 5.90E-15 | 2.28E-12 | GJB3 |
| ENSG00000188959 | 1.253180231 | 2.29E-11 | 3.66E-09 | C9orf152 |
| ENSG00000189056 | 1.30730292 | 1.81E-08 | 1.28E-06 | RELN |
| ENSG00000189127 | 1.381276305 | 1.67E-06 | 5.80E-05 | ANKRD34B |
| ENSG00000189334 | 1.05117134 | 1.17E-09 | 1.17E-07 | S100A14 |
| ENSG00000189366 | 1.090515657 | 2.55E-10 | 3.18E-08 | ALG1L |
| ENSG00000196091 | 1.041727987 | 2.01E-07 | 1.00E-05 | MYBPC1 |
| ENSG00000196136 | 1.587454731 | 4.51E-09 | 3.79E-07 | SERPINA3 |
| ENSG00000196242 | 1.226545666 | 2.88E-05 | 0.000617718 | OR2C3 |
| ENSG00000196260 | 2.18342308 | 2.79E-16 | 1.38E-13 | SFTA2 |
| ENSG00000196344 | 1.31522679 | 0.004335113 | 0.029487306 | ADH7 |
| ENSG00000196364 | 1.567983432 | 8.06E-05 | 0.00142363 | NA |
| ENSG00000196420 | 1.268599173 | 1.57E-13 | 4.40E-11 | S100A5 |
| ENSG00000196542 | 1.275358674 | 2.56E-08 | 1.73E-06 | SPTSSB |
| ENSG00000196711 | 1.277253598 | 5.40E-06 | 0.000155053 | ALKAL1 |
| ENSG00000196748 | 1.502867922 | 0.000874407 | 0.009010123 | CLPSL2 |
| ENSG00000196754 | 1.604786816 | 2.61E-14 | 8.84E-12 | S100A2 |
| ENSG00000196767 | 1.459202163 | 0.008792923 | 0.048775173 | POU3F4 |
| ENSG00000196917 | 1.672120139 | 1.22E-18 | 9.26E-16 | HCAR1 |
| ENSG00000197079 | 1.322333103 | 0.000248941 | 0.003477537 | KRT35 |
| ENSG00000197172 | 1.870669785 | 0.003045823 | 0.02285608 | MAGEA6 |
| ENSG00000197308 | 2.225920326 | 9.17E-09 | 7.09E-07 | GATA3-AS1 |
| ENSG00000197320 | 1.079034156 | 0.00510605 | 0.03315675 | NA |
| ENSG00000197430 | 1.601106558 | 0.000545755 | 0.006334172 | OPALIN |
| ENSG00000197444 | 1.111043279 | 5.67E-10 | 6.28E-08 | OGDHL |
| ENSG00000197549 | 1.063077213 | 0.000208068 | 0.003016212 | PRAMENP |
| ENSG00000197588 | 3.254101858 | 6.61E-14 | 2.05E-11 | KLKP1 |
| ENSG00000197721 | 1.450066671 | 7.67E-13 | 1.79E-10 | CR1L |
| ENSG00000198099 | 2.599600342 | 7.82E-16 | 3.60E-13 | ADH4 |
| ENSG00000198398 | 1.931494128 | 0.002864887 | 0.021854512 | TMEM207 |
| ENSG00000198681 | 1.701717816 | 0.006899976 | 0.041138198 | MAGEA1 |
| ENSG00000198691 | 1.556976776 | 8.33E-15 | 3.04E-12 | ABCA4 |
| ENSG00000198732 | 2.157264939 | 2.43E-18 | 1.77E-15 | SMOC1 |
| ENSG00000198765 | 1.13710348 | 0.00377703 | 0.026715513 | SYCP1 |
| ENSG00000198780 | 1.287665557 | 1.64E-08 | 1.17E-06 | FAM169A |
| ENSG00000198910 | 1.55381997 | 2.73E-08 | 1.82E-06 | L1CAM |
| ENSG00000199740 | -1.010781228 | 0.001548902 | 0.013871677 | NA |
| ENSG00000200070 | -1.030633853 | 0.001554556 | 0.013907937 | NA |
| ENSG00000200789 | -1.039231731 | 0.001439997 | 0.013138316 | NA |
| ENSG00000201586 | -1.083936577 | 7.00E-06 | 0.00019196 | NA |
| ENSG00000202514 | -1.003468386 | 0.001251355 | 0.011810007 | NA |
| ENSG00000203697 | 1.681064947 | 1.87E-13 | 5.03E-11 | CAPN8 |
| ENSG00000203805 | 1.644985008 | 1.04E-12 | 2.32E-10 | PLPP4 |
| ENSG00000203837 | 1.874688933 | 0.001349628 | 0.012520555 | PNLIPRP3 |
| ENSG00000204065 | 1.315446708 | 2.39E-05 | 0.00052881 | TCEAL5 |
| ENSG00000204323 | 1.647037038 | 2.65E-12 | 5.39E-10 | SMIM5 |
| ENSG00000204511 | 2.684592902 | 1.59E-16 | 8.42E-14 | MCCD1 |
| ENSG00000204544 | 1.168036158 | 0.000860063 | 0.008895688 | MUC21 |
| ENSG00000204603 | 1.416488986 | 4.25E-06 | 0.000127789 | LINC01257 |
| ENSG00000204612 | -1.154317038 | 0.001204291 | 0.011469377 | FOXB2 |
| ENSG00000204655 | 3.828196603 | 6.61E-26 | 1.50E-22 | MOG |
| ENSG00000204934 | 1.0469518 | 2.11E-14 | 7.27E-12 | ATP6V0E2-AS1 |
| ENSG00000204941 | 2.138572603 | 4.48E-08 | 2.73E-06 | PSG5 |
| ENSG00000204983 | 1.027732376 | 0.000734648 | 0.007940692 | PRSS1 |
| ENSG00000205363 | 1.910172929 | 2.43E-19 | 2.00E-16 | INSYN1 |
| ENSG00000205420 | 1.297245476 | 0.001433359 | 0.013090102 | KRT6A |
| ENSG00000205847 | 1.242368767 | 0.00134086 | 0.012460142 | OR7E91P |
| ENSG00000206073 | 1.202794126 | 0.006509614 | 0.039486486 | SERPINB4 |
| ENSG00000206075 | 1.584187962 | 8.03E-08 | 4.57E-06 | SERPINB5 |
| ENSG00000206159 | 2.287370739 | 4.31E-07 | 1.91E-05 | GYG2P1 |
| ENSG00000206192 | 1.365208727 | 0.000142287 | 0.00222257 | NA |
| ENSG00000206579 | 1.213981389 | 3.52E-07 | 1.60E-05 | XKR4 |
| ENSG00000207020 | -1.027445453 | 2.00E-05 | 0.000457258 | NA |
| ENSG00000207780 | -1.191146758 | 0.000477352 | 0.005749633 | MIR648 |
| ENSG00000207873 | -1.043532094 | 0.001172262 | 0.011236286 | MIR513A1 |
| ENSG00000211452 | 1.305863958 | 6.69E-08 | 3.90E-06 | DIO1 |
| ENSG00000211649 | -1.221657287 | 1.87E-06 | 6.41E-05 | NA |
| ENSG00000211656 | -1.185322482 | 0.000182177 | 0.002714127 | NA |
| ENSG00000211891 | 1.701141457 | 4.52E-08 | 2.75E-06 | NA |
| ENSG00000211930 | -1.30565921 | 0.001600654 | 0.014195577 | NA |
| ENSG00000211968 | -1.068756322 | 0.000182956 | 0.002722588 | NA |
| ENSG00000211976 | -1.236048473 | 2.24E-07 | 1.10E-05 | NA |
| ENSG00000212769 | -1.163479731 | 2.86E-05 | 0.000613666 | NA |
| ENSG00000213023 | 1.075105458 | 9.03E-08 | 5.04E-06 | SYT3 |
| ENSG00000213130 | 1.332841096 | 0.000333279 | 0.004366493 | NA |
| ENSG00000213148 | 2.194134573 | 2.07E-05 | 0.000469018 | NA |
| ENSG00000213332 | 1.353201955 | 5.35E-06 | 0.00015408 | NA |
| ENSG00000213673 | 1.003273539 | 1.31E-06 | 4.71E-05 | NA |
| ENSG00000214049 | 1.516944278 | 6.19E-07 | 2.58E-05 | UCA1 |
| ENSG00000214064 | -1.006906504 | 0.000748673 | 0.008038775 | NA |
| ENSG00000214128 | 2.371792908 | 4.20E-09 | 3.55E-07 | TMEM213 |
| ENSG00000214336 | 1.30658729 | 0.00027831 | 0.003784779 | FOXI3 |
| ENSG00000214429 | 1.993667759 | 5.75E-07 | 2.44E-05 | NA |
| ENSG00000214575 | 1.735510557 | 1.82E-13 | 4.93E-11 | CPEB1 |
| ENSG00000214814 | 1.005828021 | 3.38E-05 | 0.000696953 | FER1L6 |
| ENSG00000214978 | -2.085314001 | 6.56E-06 | 0.000181993 | GSG1L2 |
| ENSG00000215117 | 4.011265725 | 8.94E-10 | 9.29E-08 | NA |
| ENSG00000215262 | 1.670900567 | 0.000143992 | 0.002244672 | KCNU1 |
| ENSG00000215277 | 1.073042219 | 9.64E-08 | 5.35E-06 | RNF212B |
| ENSG00000215409 | 1.110424163 | 2.36E-05 | 0.000523107 | NA |
| ENSG00000215644 | 2.579089828 | 3.37E-10 | 4.03E-08 | GCGR |
| ENSG00000218357 | 1.208232643 | 6.89E-07 | 2.80E-05 | LINC01644 |
| ENSG00000219159 | 2.293491076 | 1.47E-08 | 1.07E-06 | NA |
| ENSG00000221826 | 1.369775937 | 0.000442655 | 0.005422909 | PSG3 |
| ENSG00000221867 | 2.354751514 | 0.000293931 | 0.003954116 | MAGEA3 |
| ENSG00000222920 | -1.122837358 | 0.005987948 | 0.037243401 | NA |
| ENSG00000223572 | 2.119954916 | 3.29E-11 | 5.01E-09 | CKMT1A |
| ENSG00000223726 | 1.548626455 | 0.000471163 | 0.005683935 | NA |
| ENSG00000223728 | 1.220703753 | 0.000169886 | 0.00256561 | NA |
| ENSG00000223783 | 1.731001908 | 0.001825933 | 0.015620393 | NA |
| ENSG00000223786 | -1.1568842 | 0.004668816 | 0.03112382 | LOC101928516 |
| ENSG00000224127 | -1.322774538 | 0.000118596 | 0.001932924 | NA |
| ENSG00000224149 | -1.062700324 | 0.002287224 | 0.018603241 | NA |
| ENSG00000224184 | 1.330042105 | 2.60E-09 | 2.35E-07 | MIR3681HG |
| ENSG00000224265 | 1.378375962 | 0.007263481 | 0.042654477 | NA |
| ENSG00000224269 | 1.470325808 | 0.007643784 | 0.04428265 | NA |
| ENSG00000224309 | 1.896281694 | 0.000335701 | 0.004394078 | NA |
| ENSG00000224310 | 1.165260633 | 2.16E-05 | 0.000486286 | LINC01567 |
| ENSG00000224318 | 2.095977304 | 2.56E-05 | 0.000556906 | CHL1-AS2 |
| ENSG00000224361 | 1.048473564 | 0.001124119 | 0.010882706 | NA |
| ENSG00000224396 | 1.31849955 | 0.006583188 | 0.039733364 | NA |
| ENSG00000224511 | 1.222743067 | 7.33E-08 | 4.25E-06 | LINC00365 |
| ENSG00000224611 | -1.02347517 | 1.90E-06 | 6.51E-05 | NA |
| ENSG00000224652 | 2.018386285 | 3.07E-10 | 3.75E-08 | LINC00885 |
| ENSG00000224731 | 1.564668678 | 0.002931361 | 0.022230064 | NA |
| ENSG00000224984 | 1.74749282 | 0.000940004 | 0.009504733 | NA |
| ENSG00000225005 | 1.33710248 | 2.90E-07 | 1.37E-05 | NA |
| ENSG00000225064 | 3.578527382 | 2.28E-09 | 2.09E-07 | NA |
| ENSG00000225117 | 1.214443637 | 0.002107752 | 0.017463455 | NA |
| ENSG00000225206 | 1.535745281 | 7.25E-05 | 0.001312661 | MIR137HG |
| ENSG00000225208 | 1.077731544 | 0.004672419 | 0.031130144 | NA |
| ENSG00000225362 | 2.046462657 | 6.13E-14 | 1.94E-11 | CT62 |
| ENSG00000225606 | 1.169898429 | 2.47E-06 | 8.08E-05 | NA |
| ENSG00000225637 | 1.134845506 | 5.30E-05 | 0.001014489 | NA |
| ENSG00000225930 | 1.033066896 | 0.005554242 | 0.035247787 | LINC02249 |
| ENSG00000225972 | 1.80159467 | 6.87E-11 | 9.77E-09 | NA |
| ENSG00000225982 | 1.128782115 | 0.001595503 | 0.014162882 | NA |
| ENSG00000226022 | 1.702044826 | 0.000634121 | 0.007104654 | NA |
| ENSG00000226032 | 1.311431017 | 6.16E-06 | 0.000173231 | NA |
| ENSG00000226040 | 1.098867235 | 5.40E-06 | 0.000155053 | NA |
| ENSG00000226057 | 1.096469731 | 0.003924419 | 0.027451767 | NA |
| ENSG00000226087 | 2.524215392 | 2.24E-10 | 2.85E-08 | NA |
| ENSG00000226242 | -1.837420102 | 7.87E-05 | 0.001400125 | NA |
| ENSG00000226281 | 1.375407248 | 8.73E-08 | 4.91E-06 | NA |
| ENSG00000226308 | 1.162162457 | 0.004891779 | 0.032123695 | NA |
| ENSG00000226397 | 2.002700792 | 4.14E-08 | 2.54E-06 | C12orf77 |
| ENSG00000226416 | 1.100835368 | 6.45E-06 | 0.000179419 | MRPL23-AS1 |
| ENSG00000226496 | 1.58602587 | 1.14E-12 | 2.48E-10 | LINC00323 |
| ENSG00000226510 | 1.008993531 | 0.000263261 | 0.003632491 | UPK1A-AS1 |
| ENSG00000226562 | 1.368386013 | 4.58E-05 | 0.000900315 | NA |
| ENSG00000226652 | 1.005042486 | 4.22E-05 | 0.000840469 | NA |
| ENSG00000226741 | 1.003123619 | 0.005983965 | 0.037229096 | LINC02554 |
| ENSG00000226816 | 1.068861848 | 2.67E-06 | 8.61E-05 | NA |
| ENSG00000226887 | 1.086059329 | 6.14E-05 | 0.00114621 | ERVMER34-1 |
| ENSG00000226953 | 1.252329094 | 8.12E-12 | 1.46E-09 | NCKAP5-AS2 |
| ENSG00000227131 | 1.361061958 | 5.60E-07 | 2.39E-05 | LOC105375050 |
| ENSG00000227243 | 1.783869432 | 0.000732717 | 0.007931305 | NA |
| ENSG00000227293 | 3.006367711 | 1.35E-05 | 0.00032895 | NA |
| ENSG00000227300 | 1.977813084 | 5.98E-05 | 0.001123709 | NA |
| ENSG00000227338 | 1.419024465 | 1.08E-05 | 0.00027303 | NA |
| ENSG00000227342 | 1.950306681 | 0.007846287 | 0.045037548 | LINC00307 |
| ENSG00000227375 | 1.201510196 | 9.11E-10 | 9.37E-08 | DLG1-AS1 |
| ENSG00000227418 | -1.524497718 | 2.69E-05 | 0.000579907 | PCGEM1 |
| ENSG00000227489 | -1.098335069 | 1.05E-05 | 0.00026827 | NA |
| ENSG00000227555 | 1.576637465 | 1.55E-05 | 0.000369736 | MIR4290HG |
| ENSG00000227653 | 1.04079576 | 0.00029724 | 0.003987527 | NA |
| ENSG00000227857 | 1.255768579 | 3.08E-10 | 3.75E-08 | LOC101929626 |
| ENSG00000227925 | -1.139649984 | 0.000632593 | 0.007097819 | NA |
| ENSG00000228044 | 1.282316869 | 1.22E-05 | 0.000301262 | LOC101927787 |
| ENSG00000228211 | 2.486668124 | 0.000286226 | 0.003869299 | NA |
| ENSG00000228278 | 1.406771899 | 3.06E-07 | 1.43E-05 | ORM2 |
| ENSG00000228349 | 2.185127786 | 4.15E-06 | 0.000125268 | NA |
| ENSG00000228420 | 1.854840107 | 2.23E-05 | 0.000498919 | NA |
| ENSG00000228476 | 1.370210897 | 0.000505734 | 0.005999932 | NA |
| ENSG00000228799 | 1.27599111 | 0.003400118 | 0.02476515 | NA |
| ENSG00000228973 | 1.45647204 | 0.001059241 | 0.010397628 | NA |
| ENSG00000229032 | 1.292615616 | 0.003495617 | 0.025256205 | NA |
| ENSG00000229314 | 1.261956551 | 0.000106267 | 0.001771537 | ORM1 |
| ENSG00000229373 | 1.052968115 | 1.26E-05 | 0.000310913 | LINC00452 |
| ENSG00000229418 | 2.925237205 | 3.05E-07 | 1.43E-05 | NA |
| ENSG00000229642 | 1.907940563 | 8.32E-05 | 0.001458771 | NA |
| ENSG00000229740 | -1.170119104 | 0.002068182 | 0.017224162 | NA |
| ENSG00000229941 | 1.620109454 | 7.31E-06 | 0.00019907 | LOC105373764 |
| ENSG00000229970 | 1.148910048 | 0.003206479 | 0.023761981 | NA |
| ENSG00000230027 | 1.967936852 | 6.45E-08 | 3.80E-06 | NA |
| ENSG00000230286 | 2.483897655 | 1.34E-05 | 0.000327908 | NA |
| ENSG00000230453 | 1.200787554 | 1.28E-05 | 0.000314744 | ANKRD18B |
| ENSG00000230461 | 1.088477746 | 0.000155636 | 0.002390537 | PROX1-AS1 |
| ENSG00000230500 | 1.293596811 | 0.000705562 | 0.007714933 | NA |
| ENSG00000230805 | 2.251944268 | 9.73E-05 | 0.001654207 | NA |
| ENSG00000230843 | 2.69344861 | 0.000716909 | 0.007797289 | NA |
| ENSG00000230916 | 1.359632818 | 8.46E-10 | 8.88E-08 | NA |
| ENSG00000230937 | 1.439621713 | 0.00012088 | 0.001961061 | MIR205HG |
| ENSG00000231062 | 1.21790292 | 2.01E-06 | 6.79E-05 | NA |
| ENSG00000231270 | -1.40997811 | 0.0010922 | 0.010629575 | NA |
| ENSG00000231317 | 1.411864057 | 0.007913214 | 0.045285696 | NA |
| ENSG00000231322 | 2.091214334 | 1.14E-05 | 0.000286431 | RPL13AP17 |
| ENSG00000231431 | 2.035447167 | 5.76E-08 | 3.44E-06 | LOC440910 |
| ENSG00000231458 | 1.196962331 | 0.001450551 | 0.013209684 | NA |
| ENSG00000231561 | 1.654018007 | 3.49E-08 | 2.21E-06 | NA |
| ENSG00000231720 | 1.13569221 | 0.006904238 | 0.04115726 | NA |
| ENSG00000231826 | 1.472217838 | 9.27E-12 | 1.65E-09 | LINC01819 |
| ENSG00000231870 | 2.017147049 | 1.15E-06 | 4.24E-05 | NA |
| ENSG00000231882 | 1.018506594 | 0.000261714 | 0.003615015 | NA |
| ENSG00000231982 | 1.199111964 | 6.31E-09 | 5.13E-07 | NA |
| ENSG00000232131 | 1.553192447 | 4.70E-06 | 0.000139255 | NCOA7-AS1 |
| ENSG00000232177 | 1.542858741 | 5.36E-12 | 9.91E-10 | NA |
| ENSG00000232188 | 1.434383817 | 0.00188023 | 0.01594382 | NA |
| ENSG00000232328 | 1.049523386 | 0.007817435 | 0.044937686 | NA |
| ENSG00000232352 | 1.882855753 | 2.55E-17 | 1.64E-14 | SEMA3B-AS1 |
| ENSG00000232835 | 1.673125843 | 0.006881312 | 0.041045899 | NA |
| ENSG00000233017 | 1.479520985 | 0.001759448 | 0.015196245 | NA |
| ENSG00000233101 | 1.751548055 | 5.11E-18 | 3.59E-15 | HOXB-AS3 |
| ENSG00000233125 | 3.283788092 | 5.84E-26 | 1.41E-22 | NA |
| ENSG00000233146 | 2.415664097 | 2.88E-05 | 0.000617671 | NA |
| ENSG00000233215 | 1.733315789 | 1.13E-05 | 0.000283752 | LINC01687 |
| ENSG00000233258 | -1.263010408 | 1.97E-05 | 0.000451585 | NA |
| ENSG00000233332 | 2.347446578 | 6.12E-08 | 3.63E-06 | NA |
| ENSG00000233526 | -1.117448662 | 0.006388753 | 0.038979558 | NA |
| ENSG00000233544 | 1.21378211 | 1.80E-05 | 0.00041788 | NA |
| ENSG00000233569 | 1.327236948 | 3.12E-05 | 0.000656261 | LOC101928797 |
| ENSG00000233574 | -1.530289103 | 0.001713819 | 0.014928754 | NA |
| ENSG00000233725 | 1.968673536 | 1.54E-14 | 5.45E-12 | NA |
| ENSG00000233850 | 1.358314541 | 1.43E-08 | 1.05E-06 | NA |
| ENSG00000233920 | 1.695449925 | 0.000265145 | 0.003655888 | NA |
| ENSG00000233970 | 1.417268796 | 0.000104133 | 0.001745759 | NA |
| ENSG00000234159 | -1.042473945 | 0.001298072 | 0.012144229 | NA |
| ENSG00000234190 | 1.100811529 | 8.55E-05 | 0.001488638 | NA |
| ENSG00000234192 | 1.115790174 | 6.18E-05 | 0.001152422 | NA |
| ENSG00000234233 | 2.05331793 | 1.80E-05 | 0.000417776 | NA |
| ENSG00000234602 | 1.160317912 | 0.000406132 | 0.005062054 | MCIDAS |
| ENSG00000234694 | 1.007574845 | 3.29E-14 | 1.09E-11 | NA |
| ENSG00000234711 | 1.335609153 | 2.32E-05 | 0.000517129 | NA |
| ENSG00000234965 | 1.088869207 | 1.40E-05 | 0.000339101 | SHISA8 |
| ENSG00000234979 | 2.873085742 | 3.82E-05 | 0.000773279 | NA |
| ENSG00000235026 | 1.458164382 | 0.004355963 | 0.029583262 | DPP10-AS1 |
| ENSG00000235034 | 1.117235155 | 1.30E-05 | 0.000319672 | C19orf81 |
| ENSG00000235263 | 1.804246229 | 8.59E-07 | 3.36E-05 | NA |
| ENSG00000235280 | 1.364290304 | 1.71E-11 | 2.86E-09 | NA |
| ENSG00000235366 | 1.533264073 | 3.24E-07 | 1.50E-05 | NA |
| ENSG00000235876 | -1.46741141 | 0.000612905 | 0.006925561 | NA |
| ENSG00000236053 | 1.129304302 | 0.00826058 | 0.0467632 | NA |
| ENSG00000236123 | 1.502909124 | 0.007809633 | 0.044913808 | NA |
| ENSG00000236208 | 2.396688443 | 1.89E-05 | 0.000435615 | NA |
| ENSG00000236279 | 1.053672349 | 8.41E-05 | 0.001471964 | CLEC2L |
| ENSG00000236283 | 1.339599685 | 1.10E-10 | 1.51E-08 | NA |
| ENSG00000236427 | 1.410970103 | 2.18E-08 | 1.50E-06 | NA |
| ENSG00000236507 | 2.2455302 | 0.001417438 | 0.012999958 | LOC107987032 |
| ENSG00000236516 | 1.108192445 | 0.002168178 | 0.017811478 | NA |
| ENSG00000236550 | -1.160838619 | 0.007371638 | 0.043086816 | NA |
| ENSG00000236595 | 2.484894569 | 0.0009271 | 0.009403757 | NA |
| ENSG00000236604 | -1.078311405 | 0.001418918 | 0.013010448 | NA |
| ENSG00000236886 | -1.036437993 | 0.001957514 | 0.016487111 | NA |
| ENSG00000236922 | 1.626270556 | 7.73E-05 | 0.001378336 | NA |
| ENSG00000236939 | 1.31014926 | 4.77E-12 | 9.00E-10 | BAALC-AS2 |
| ENSG00000237058 | 3.532110377 | 3.64E-08 | 2.29E-06 | NA |
| ENSG00000237136 | 1.020677182 | 1.76E-07 | 8.88E-06 | C4orf51 |
| ENSG00000237170 | 1.038061547 | 2.63E-06 | 8.49E-05 | NA |
| ENSG00000237289 | 2.393501444 | 4.00E-14 | 1.30E-11 | CKMT1B |
| ENSG00000237320 | -1.066386676 | 0.000434311 | 0.005350269 | LOC105374461 |
| ENSG00000237330 | 1.797610823 | 3.55E-10 | 4.21E-08 | RNF223 |
| ENSG00000237412 | 2.169938024 | 3.38E-08 | 2.16E-06 | PRSS56 |
| ENSG00000237463 | 2.415192016 | 2.18E-07 | 1.07E-05 | LRRC52-AS1 |
| ENSG00000237636 | 1.169271699 | 0.001788397 | 0.015370675 | NA |
| ENSG00000237667 | 1.390541253 | 0.001469905 | 0.013341949 | LINC01115 |
| ENSG00000237707 | 1.116275546 | 5.74E-05 | 0.001085596 | LOC101928596 |
| ENSG00000238021 | 1.517025785 | 3.32E-08 | 2.13E-06 | NA |
| ENSG00000238117 | -1.091187824 | 0.000652682 | 0.007260032 | NA |
| ENSG00000238271 | 1.004130173 | 0.000246318 | 0.003450412 | NA |
| ENSG00000238276 | 1.48727131 | 0.001610791 | 0.014253035 | LOC101927760 |
| ENSG00000240253 | 1.338517849 | 3.97E-07 | 1.78E-05 | NA |
| ENSG00000240268 | -1.349594699 | 0.000106139 | 0.001770173 | MOXD2P |
| ENSG00000240405 | 1.552773436 | 8.84E-08 | 4.95E-06 | SAMMSON |
| ENSG00000240427 | 1.860379912 | 0.000124872 | 0.002003132 | NA |
| ENSG00000240533 | -1.039299382 | 0.001944837 | 0.016398192 | NA |
| ENSG00000240747 | 1.152396594 | 3.06E-10 | 3.75E-08 | KRBOX1 |
| ENSG00000241168 | -1.551051851 | 0.000909901 | 0.009285313 | NA |
| ENSG00000241233 | 1.011008287 | 1.39E-05 | 0.00033765 | KRTAP5-8 |
| ENSG00000241439 | 2.457087359 | 1.19E-06 | 4.37E-05 | NA |
| ENSG00000241475 | 1.942316069 | 0.000470578 | 0.005680426 | NA |
| ENSG00000241598 | 1.408237068 | 0.000477972 | 0.00575531 | KRTAP5-4 |
| ENSG00000241697 | 1.271461087 | 6.09E-07 | 2.55E-05 | TMEFF1 |
| ENSG00000242078 | 1.64406819 | 4.36E-06 | 0.000130434 | NA |
| ENSG00000242136 | 1.853473857 | 0.000622071 | 0.007016389 | NA |
| ENSG00000242366 | -1.405292278 | 1.24E-06 | 4.51E-05 | UGT1A8 |
| ENSG00000242407 | 2.089680411 | 3.64E-05 | 0.000743393 | NA |
| ENSG00000243137 | 1.259502917 | 0.000177635 | 0.002661835 | PSG4 |
| ENSG00000243225 | 1.046611457 | 6.62E-07 | 2.73E-05 | NA |
| ENSG00000243550 | 2.506423889 | 2.15E-10 | 2.74E-08 | NA |
| ENSG00000243709 | 1.854433241 | 8.00E-15 | 2.95E-12 | LEFTY1 |
| ENSG00000243766 | 1.621609988 | 1.39E-07 | 7.35E-06 | NA |
| ENSG00000243988 | 1.81669703 | 6.77E-13 | 1.60E-10 | NA |
| ENSG00000244155 | 2.110655794 | 2.23E-05 | 0.000499086 | NA |
| ENSG00000244281 | -1.617125711 | 0.000886827 | 0.009107524 | NA |
| ENSG00000244361 | -1.206225638 | 7.08E-08 | 4.12E-06 | NA |
| ENSG00000244414 | 1.187470957 | 0.000122737 | 0.001981206 | CFHR1 |
| ENSG00000244998 | 1.307568842 | 4.79E-11 | 7.04E-09 | NA |
| ENSG00000246820 | 1.140695728 | 0.001217236 | 0.011569876 | NA |
| ENSG00000247311 | 1.408657559 | 5.73E-07 | 2.43E-05 | NA |
| ENSG00000248127 | 1.363966912 | 0.002999361 | 0.022594138 | NA |
| ENSG00000248144 | 2.535307553 | 5.48E-21 | 6.23E-18 | ADH1C |
| ENSG00000248216 | -1.027783461 | 0.003866566 | 0.027160062 | NA |
| ENSG00000248461 | 1.759821687 | 0.001692605 | 0.014798192 | LINC02119 |
| ENSG00000248596 | 1.274941223 | 2.45E-06 | 8.03E-05 | LOC643201 |
| ENSG00000248713 | 2.008171292 | 4.83E-06 | 0.000141988 | C4orf54 |
| ENSG00000248747 | 2.030052198 | 0.00489145 | 0.032123695 | NA |
| ENSG00000248827 | 1.01407002 | 0.000366727 | 0.004685677 | NA |
| ENSG00000248994 | 1.04035135 | 0.000944295 | 0.009534774 | LOC105374618 |
| ENSG00000249131 | -1.159323621 | 6.28E-05 | 0.001164354 | NA |
| ENSG00000249341 | 1.266951242 | 0.001025968 | 0.010148426 | LOC100506444 |
| ENSG00000249396 | -1.035701349 | 0.000198397 | 0.002897731 | LINC02212 |
| ENSG00000249518 | -1.494430349 | 0.000133917 | 0.002117486 | NA |
| ENSG00000249599 | 1.698744572 | 0.000633318 | 0.007101827 | BMPR1B-DT |
| ENSG00000249601 | 1.191744801 | 0.002478882 | 0.019695233 | NA |
| ENSG00000249699 | 1.000893136 | 0.00507788 | 0.033040389 | LINC02261 |
| ENSG00000249738 | 1.639254305 | 4.13E-11 | 6.19E-09 | LOC285626 |
| ENSG00000249937 | 2.999511283 | 5.01E-10 | 5.63E-08 | NA |
| ENSG00000250007 | 1.462539087 | 4.63E-05 | 0.000907369 | LOC101928174 |
| ENSG00000250230 | 1.496509474 | 3.72E-05 | 0.000757649 | LOC101927495 |
| ENSG00000250244 | 1.306486833 | 0.003014753 | 0.022671316 | NA |
| ENSG00000250266 | 1.938710329 | 0.000527981 | 0.006184194 | LINC01612 |
| ENSG00000250292 | -1.067371831 | 0.000431123 | 0.005318699 | NA |
| ENSG00000250358 | 1.49024438 | 4.34E-05 | 0.000858128 | NA |
| ENSG00000250658 | -1.093157626 | 0.000166246 | 0.002522446 | NA |
| ENSG00000250866 | 1.937883645 | 0.000250109 | 0.003489613 | NA |
| ENSG00000251061 | 2.283734436 | 3.88E-05 | 0.000781507 | NA |
| ENSG00000251093 | -1.115262597 | 2.45E-05 | 0.000540075 | NA |
| ENSG00000251142 | 1.913445796 | 0.003947281 | 0.027569173 | NA |
| ENSG00000251144 | -1.048701137 | 0.008589958 | 0.048031342 | NA |
| ENSG00000251258 | -1.357121977 | 9.18E-07 | 3.55E-05 | RFPL4B |
| ENSG00000251303 | 1.080171889 | 0.000166636 | 0.002527373 | NA |
| ENSG00000251423 | 1.287600101 | 0.004852632 | 0.031972812 | NA |
| ENSG00000251491 | 1.921446782 | 0.000196732 | 0.002880583 | NA |
| ENSG00000251504 | 1.119920614 | 0.000162474 | 0.002472009 | LINC01099 |
| ENSG00000251629 | 1.059838984 | 0.005946053 | 0.037060635 | LINC02241 |
| ENSG00000251664 | 1.154746327 | 1.83E-07 | 9.19E-06 | PCDHA12 |
| ENSG00000252560 | -1.059902974 | 0.005719128 | 0.036049453 | NA |
| ENSG00000253138 | 1.358389917 | 2.21E-05 | 0.000495354 | LINC00967 |
| ENSG00000253154 | 1.452312955 | 1.39E-06 | 4.94E-05 | NA |
| ENSG00000253209 | -1.212252112 | 0.002243659 | 0.018322584 | NA |
| ENSG00000253270 | 1.65786145 | 4.80E-05 | 0.00093324 | NA |
| ENSG00000253301 | 1.319875314 | 0.004068827 | 0.028211895 | LINC01606 |
| ENSG00000253368 | 1.149509627 | 1.03E-08 | 7.82E-07 | TRNP1 |
| ENSG00000253477 | 1.535631844 | 7.88E-05 | 0.001401615 | NA |
| ENSG00000253495 | -1.206316575 | 0.000148357 | 0.002303235 | NA |
| ENSG00000253504 | 1.023955314 | 0.005719295 | 0.036049453 | NA |
| ENSG00000253585 | 1.011695529 | 0.001750245 | 0.015136961 | NA |
| ENSG00000253802 | 1.444341336 | 0.005015144 | 0.032720262 | SIRLNT |
| ENSG00000253821 | 1.309223385 | 0.000236349 | 0.003339806 | NA |
| ENSG00000253901 | 1.569811381 | 0.000370129 | 0.004722211 | NA |
| ENSG00000253973 | 1.127166529 | 0.000244853 | 0.003436119 | NA |
| ENSG00000254024 | 1.922620021 | 0.000244077 | 0.003427715 | NA |
| ENSG00000254127 | -1.03852385 | 0.003874073 | 0.027197975 | NA |
| ENSG00000254153 | 1.122068563 | 0.000789702 | 0.008356473 | NA |
| ENSG00000254228 | -1.036136876 | 0.001959007 | 0.016496096 | NA |
| ENSG00000254321 | 1.357892194 | 0.000139394 | 0.002190463 | NA |
| ENSG00000254489 | 1.948168256 | 2.15E-08 | 1.49E-06 | MPPED2-AS1 |
| ENSG00000254528 | 1.270567201 | 7.57E-10 | 8.02E-08 | NA |
| ENSG00000254542 | 1.083738288 | 0.002741056 | 0.021166715 | NA |
| ENSG00000255007 | 1.668945801 | 1.41E-05 | 0.000341075 | NA |
| ENSG00000255202 | 1.331507925 | 5.48E-09 | 4.52E-07 | NA |
| ENSG00000255394 | 1.487261779 | 0.004095781 | 0.028337821 | C8orf49 |
| ENSG00000255480 | 1.981449135 | 1.97E-06 | 6.67E-05 | NA |
| ENSG00000255790 | 2.757559627 | 4.51E-11 | 6.70E-09 | NA |
| ENSG00000255847 | -1.024022074 | 3.23E-05 | 0.0006753 | NA |
| ENSG00000255946 | 1.459179693 | 8.33E-07 | 3.28E-05 | NA |
| ENSG00000255947 | 1.14428406 | 0.002211835 | 0.018104751 | NA |
| ENSG00000255951 | -1.162348243 | 0.001133392 | 0.010950557 | NA |
| ENSG00000256004 | -1.042335313 | 0.005580713 | 0.035377752 | NA |
| ENSG00000256193 | 1.805759913 | 0.000519417 | 0.006111646 | LINC00507 |
| ENSG00000256249 | 1.619806078 | 3.66E-05 | 0.000745644 | NA |
| ENSG00000256463 | 2.877619346 | 3.40E-10 | 4.06E-08 | SALL3 |
| ENSG00000257048 | 1.007024937 | 0.000827099 | 0.008620012 | NA |
| ENSG00000257084 | 2.541797849 | 4.20E-07 | 1.87E-05 | MIR200CHG |
| ENSG00000257119 | 1.110723197 | 0.000755049 | 0.008094826 | NA |
| ENSG00000257137 | 1.128198359 | 0.000674131 | 0.007451067 | C12orf80 |
| ENSG00000257588 | 1.831255102 | 0.000402974 | 0.005032427 | LOC101927318 |
| ENSG00000257636 | 1.418005095 | 0.0011558 | 0.011103256 | G2E3-AS1 |
| ENSG00000257671 | 1.054826854 | 3.87E-06 | 0.000118136 | KRT7-AS |
| ENSG00000258038 | 1.309180551 | 0.000401414 | 0.005019272 | NA |
| ENSG00000258053 | 1.779051862 | 7.74E-05 | 0.001380089 | NA |
| ENSG00000258144 | 1.393222445 | 0.002273218 | 0.018520959 | LINC02406 |
| ENSG00000258413 | 1.013683797 | 4.68E-08 | 2.83E-06 | NA |
| ENSG00000258483 | 1.520796364 | 0.000782199 | 0.008301954 | LINC02251 |
| ENSG00000258548 | 1.271158225 | 0.000311931 | 0.004151493 | LINC00645 |
| ENSG00000258602 | 1.450353404 | 3.53E-07 | 1.60E-05 | LINC01629 |
| ENSG00000258604 | 1.218106998 | 3.80E-06 | 0.000116275 | NA |
| ENSG00000258616 | 1.376563516 | 0.000659388 | 0.007311489 | LINC02303 |
| ENSG00000258636 | 1.329816744 | 8.78E-08 | 4.93E-06 | NA |
| ENSG00000258670 | 1.407583166 | 0.000326019 | 0.004300517 | NA |
| ENSG00000258675 | 1.317514499 | 4.80E-05 | 0.000933517 | NA |
| ENSG00000258691 | 1.504827032 | 1.94E-08 | 1.35E-06 | NA |
| ENSG00000258740 | 2.283497061 | 4.22E-06 | 0.000127068 | NA |
| ENSG00000258807 | 1.041687749 | 0.000904194 | 0.009236825 | NA |
| ENSG00000258829 | 3.165255423 | 2.90E-06 | 9.22E-05 | NA |
| ENSG00000258927 | 1.854880807 | 1.24E-05 | 0.000306511 | NA |
| ENSG00000258947 | 1.095534732 | 1.08E-08 | 8.15E-07 | TUBB3 |
| ENSG00000258998 | 1.366810408 | 0.000105711 | 0.001767611 | NA |
| ENSG00000259033 | 1.230096252 | 0.000454709 | 0.005538997 | NA |
| ENSG00000259038 | 1.891020794 | 9.74E-12 | 1.73E-09 | NA |
| ENSG00000259054 | 1.257831594 | 3.29E-07 | 1.52E-05 | LINC02332 |
| ENSG00000259222 | 1.213013998 | 0.002603437 | 0.020362086 | NA |
| ENSG00000259230 | 1.277271347 | 3.00E-08 | 1.95E-06 | LINC02323 |
| ENSG00000259241 | 2.20086112 | 0.000184333 | 0.002739911 | NA |
| ENSG00000259268 | 2.493153634 | 1.04E-07 | 5.74E-06 | NA |
| ENSG00000259285 | 1.831736912 | 9.98E-10 | 1.01E-07 | NA |
| ENSG00000259296 | 1.122215004 | 0.003668565 | 0.026173295 | NA |
| ENSG00000259374 | 1.460517737 | 2.85E-08 | 1.89E-06 | NA |
| ENSG00000259462 | 1.125361793 | 4.62E-05 | 0.000906336 | CPEB1-AS1 |
| ENSG00000259669 | 1.600889475 | 0.000541178 | 0.006302495 | NA |
| ENSG00000259685 | 1.667199072 | 1.70E-05 | 0.00039845 | NA |
| ENSG00000259721 | 1.55778095 | 2.53E-05 | 0.000553785 | LOC100131315 |
| ENSG00000259803 | 1.161912878 | 4.83E-06 | 0.000141988 | SLC22A31 |
| ENSG00000259925 | 2.249155493 | 1.65E-05 | 0.00039101 | NA |
| ENSG00000259937 | 1.738301302 | 0.000107763 | 0.001793383 | NA |
| ENSG00000259974 | 2.010688703 | 2.68E-10 | 3.34E-08 | LINC00261 |
| ENSG00000260057 | 1.627949436 | 0.000178127 | 0.002667144 | NA |
| ENSG00000260152 | -1.014742768 | 0.004255672 | 0.029121088 | NA |
| ENSG00000260211 | 2.058927283 | 6.20E-06 | 0.000174066 | NA |
| ENSG00000260220 | 1.231978079 | 0.000125241 | 0.002008223 | CCDC187 |
| ENSG00000260230 | 1.013275106 | 3.56E-05 | 0.000730113 | FRRS1L |
| ENSG00000260266 | 1.270019362 | 6.08E-09 | 4.96E-07 | PPIAP46 |
| ENSG00000260268 | 2.374840671 | 0.000396722 | 0.004975231 | LINC00919 |
| ENSG00000260284 | 3.096051874 | 3.15E-15 | 1.30E-12 | NA |
| ENSG00000260469 | 2.890237912 | 2.34E-13 | 6.11E-11 | NA |
| ENSG00000260592 | 1.06089482 | 4.64E-05 | 0.000909209 | NA |
| ENSG00000260658 | 1.336321428 | 1.68E-08 | 1.20E-06 | NA |
| ENSG00000260710 | 2.170174136 | 1.65E-12 | 3.52E-10 | NA |
| ENSG00000260802 | 2.243993789 | 9.01E-14 | 2.72E-11 | SERTM2 |
| ENSG00000260807 | 1.725035379 | 4.69E-20 | 4.42E-17 | LMF1 |
| ENSG00000260850 | -1.522121883 | 1.74E-08 | 1.24E-06 | NA |
| ENSG00000260973 | -1.488712258 | 0.000310138 | 0.004137605 | NA |
| ENSG00000260975 | 1.82009699 | 0.003719672 | 0.026415081 | NA |
| ENSG00000261105 | 1.012196783 | 1.91E-07 | 9.54E-06 | LMO7-AS1 |
| ENSG00000261116 | 1.568643995 | 2.06E-05 | 0.000467975 | NA |
| ENSG00000261190 | 1.347925919 | 0.005025822 | 0.032767819 | C16orf97 |
| ENSG00000261319 | -1.199637278 | 0.0004284 | 0.005288471 | NA |
| ENSG00000261399 | 1.689725698 | 7.19E-12 | 1.31E-09 | NA |
| ENSG00000261434 | 1.109015898 | 2.53E-05 | 0.000554559 | NA |
| ENSG00000261437 | 1.639271997 | 7.25E-07 | 2.92E-05 | LOC100288748 |
| ENSG00000261502 | 1.263973659 | 2.76E-07 | 1.31E-05 | NA |
| ENSG00000261514 | 1.492499833 | 0.001674071 | 0.014678388 | LINC01976 |
| ENSG00000261701 | 1.710106618 | 2.93E-07 | 1.38E-05 | HPR |
| ENSG00000261713 | 1.450490624 | 9.59E-06 | 0.000248973 | SSTR5-AS1 |
| ENSG00000261786 | 1.054565455 | 0.000191513 | 0.002822021 | NA |
| ENSG00000261804 | 1.776473828 | 1.11E-09 | 1.11E-07 | NA |
| ENSG00000261821 | 1.25049209 | 2.95E-05 | 0.000627858 | NA |
| ENSG00000262714 | 1.515954383 | 9.07E-09 | 7.03E-07 | NA |
| ENSG00000262768 | 1.624941814 | 0.000220673 | 0.003163357 | NA |
| ENSG00000262902 | 1.346449456 | 1.55E-11 | 2.64E-09 | NA |
| ENSG00000262920 | 2.127122992 | 7.86E-18 | 5.43E-15 | NA |
| ENSG00000263146 | 2.897924348 | 5.84E-07 | 2.47E-05 | NA |
| ENSG00000263155 | 1.425815767 | 4.58E-15 | 1.83E-12 | MYZAP |
| ENSG00000263312 | 2.073291433 | 3.55E-09 | 3.05E-07 | NA |
| ENSG00000263325 | 1.622622612 | 0.000127313 | 0.002034696 | NA |
| ENSG00000263429 | 1.47367674 | 1.03E-06 | 3.89E-05 | TMEM238L |
| ENSG00000263821 | 1.487527731 | 0.003264583 | 0.024081829 | NA |
| ENSG00000263862 | 2.46433801 | 4.72E-12 | 8.96E-10 | NA |
| ENSG00000263952 | 1.004400599 | 0.005411233 | 0.03457539 | NA |
| ENSG00000264566 | -1.062107884 | 0.00024332 | 0.003419571 | MIR23C |
| ENSG00000265060 | 1.578500932 | 0.006147168 | 0.037991029 | PPY2P |
| ENSG00000265417 | 1.396127245 | 2.05E-05 | 0.000465914 | NA |
| ENSG00000265460 | 2.523005229 | 1.58E-10 | 2.09E-08 | NA |
| ENSG00000265763 | 1.31930068 | 1.01E-11 | 1.78E-09 | ZNF488 |
| ENSG00000265933 | 1.188477802 | 3.28E-05 | 0.000681474 | LINC00668 |
| ENSG00000265944 | 1.736110749 | 0.001142251 | 0.010997684 | LINC01387 |
| ENSG00000266120 | 1.241578571 | 7.33E-05 | 0.001323985 | NA |
| ENSG00000266554 | 1.342673328 | 8.00E-05 | 0.001417337 | LINC01443 |
| ENSG00000266830 | 1.503687225 | 0.000840008 | 0.008733371 | NA |
| ENSG00000266968 | 2.156843875 | 6.39E-17 | 3.69E-14 | NA |
| ENSG00000267013 | -1.186076191 | 3.83E-10 | 4.48E-08 | LINC01929 |
| ENSG00000267014 | 1.307484346 | 4.92E-12 | 9.24E-10 | NA |
| ENSG00000267134 | 1.469231407 | 0.004743611 | 0.031420429 | LINC01924 |
| ENSG00000267328 | 1.073185969 | 0.000215564 | 0.003101616 | NA |
| ENSG00000267423 | 1.494708855 | 0.008320083 | 0.046990004 | NA |
| ENSG00000267424 | 1.730709271 | 0.000234926 | 0.003325766 | NA |
| ENSG00000267670 | 1.360561899 | 7.87E-07 | 3.13E-05 | NA |
| ENSG00000267750 | 2.101174908 | 8.89E-24 | 1.56E-20 | NA |
| ENSG00000267774 | 3.020940705 | 9.05E-10 | 9.33E-08 | NA |
| ENSG00000267790 | 1.356175729 | 0.000416189 | 0.005167426 | LINC01987 |
| ENSG00000267795 | 1.260988446 | 1.28E-06 | 4.64E-05 | SMIM22 |
| ENSG00000267868 | 1.190737135 | 8.40E-07 | 3.30E-05 | NA |
| ENSG00000267968 | 4.071320563 | 2.71E-10 | 3.36E-08 | LOC105372441 |
| ENSG00000268223 | 1.267379309 | 0.000822046 | 0.008581229 | ARL14EPL |
| ENSG00000268320 | 1.438130436 | 0.001196939 | 0.011421865 | SCGB1C2 |
| ENSG00000268416 | 1.302883825 | 0.000696331 | 0.007644426 | NA |
| ENSG00000268433 | 1.268960357 | 0.003181781 | 0.023624222 | NA |
| ENSG00000268686 | 1.210828507 | 2.95E-10 | 3.62E-08 | LOC101928295 |
| ENSG00000268942 | 1.037096554 | 0.004278705 | 0.029221825 | NA |
| ENSG00000268964 | 1.343788946 | 0.000305371 | 0.00408245 | ERVV-2 |
| ENSG00000268981 | 1.16533224 | 0.000148448 | 0.002303235 | NA |
| ENSG00000269235 | 1.05222839 | 1.09E-09 | 1.10E-07 | ZNF350-AS1 |
| ENSG00000269289 | 2.0151948 | 6.77E-10 | 7.32E-08 | LOC100505851 |
| ENSG00000269416 | 1.148695011 | 5.62E-07 | 2.39E-05 | LINC01224 |
| ENSG00000270038 | 1.445873783 | 0.002756484 | 0.021242598 | NA |
| ENSG00000270112 | 1.592517293 | 1.10E-07 | 6.03E-06 | NA |
| ENSG00000270182 | 1.036803599 | 2.44E-06 | 8.01E-05 | NA |
| ENSG00000270388 | 1.381757631 | 7.56E-11 | 1.06E-08 | NA |
| ENSG00000270620 | -1.059627548 | 0.001076794 | 0.010527325 | NA |
| ENSG00000270799 | -1.298103173 | 0.000274781 | 0.003752909 | NA |
| ENSG00000271714 | 2.681926999 | 6.07E-10 | 6.69E-08 | NA |
| ENSG00000271830 | 1.686119375 | 8.75E-05 | 0.001518148 | NA |
| ENSG00000271850 | 1.616361108 | 0.002650035 | 0.020622281 | LINC02343 |
| ENSG00000271901 | 1.618540646 | 0.002090439 | 0.017360919 | NA |
| ENSG00000272121 | 1.685670603 | 1.53E-05 | 0.000367214 | NA |
| ENSG00000272202 | 1.110752538 | 3.74E-05 | 0.000760695 | NA |
| ENSG00000272411 | 1.345054546 | 1.21E-06 | 4.42E-05 | NA |
| ENSG00000273259 | 1.58256684 | 3.40E-08 | 2.16E-06 | NA |
| ENSG00000273335 | 1.942946625 | 3.01E-14 | 1.01E-11 | NA |
| ENSG00000273394 | 1.64573737 | 1.22E-10 | 1.66E-08 | NA |
| ENSG00000273664 | 2.791757648 | 0.00012293 | 0.001983225 | NA |
| ENSG00000273706 | 1.517171034 | 4.23E-05 | 0.000841171 | LHX1 |
| ENSG00000273742 | -1.10044615 | 3.91E-05 | 0.000784689 | MIR6075 |
| ENSG00000273769 | 1.173015193 | 0.000800263 | 0.008428154 | LCA10 |
| ENSG00000273853 | 2.584964631 | 1.49E-07 | 7.78E-06 | NA |
| ENSG00000274002 | 2.384954762 | 6.64E-08 | 3.89E-06 | NA |
| ENSG00000274023 | -1.046893289 | 0.000526713 | 0.006178697 | NA |
| ENSG00000274173 | 2.00045918 | 2.66E-08 | 1.79E-06 | NA |
| ENSG00000274209 | 1.579452235 | 7.48E-06 | 0.000202003 | ANTXRL |
| ENSG00000274244 | 1.287248583 | 0.000168823 | 0.002552541 | NA |
| ENSG00000274248 | 1.440243509 | 1.73E-13 | 4.72E-11 | NA |
| ENSG00000274330 | 1.541646605 | 5.42E-06 | 0.000155267 | NA |
| ENSG00000274719 | 1.380615687 | 1.72E-07 | 8.76E-06 | NA |
| ENSG00000274827 | 1.288016713 | 0.00578321 | 0.036363427 | NA |
| ENSG00000274895 | 1.50412678 | 0.0003552 | 0.004583823 | NA |
| ENSG00000275427 | 1.634846043 | 0.00028461 | 0.003851501 | LOC286083 |
| ENSG00000275919 | -1.093729016 | 0.001509446 | 0.013617762 | NA |
| ENSG00000276012 | 1.017682232 | 3.18E-05 | 0.000666212 | NA |
| ENSG00000276223 | 1.529865447 | 2.86E-08 | 1.89E-06 | NA |
| ENSG00000276289 | 1.460087899 | 2.55E-06 | 8.28E-05 | KCNE1B |
| ENSG00000277011 | 2.000865632 | 6.70E-09 | 5.40E-07 | NA |
| ENSG00000277268 | 1.515036966 | 0.00012383 | 0.001993532 | LHX1-DT |
| ENSG00000277882 | -1.046187756 | 2.55E-05 | 0.000556036 | NA |
| ENSG00000278012 | 1.390864365 | 0.00317249 | 0.023577865 | NA |
| ENSG00000278054 | 1.117627492 | 0.000409662 | 0.00510112 | NA |
| ENSG00000278196 | -1.083401119 | 1.08E-05 | 0.000273969 | NA |
| ENSG00000278552 | 1.384007483 | 0.001386184 | 0.01278304 | NA |
| ENSG00000278898 | 1.384081577 | 9.79E-05 | 0.001662729 | NA |
| ENSG00000278910 | 1.048126718 | 2.10E-05 | 0.000474919 | BANCR |
| ENSG00000279151 | 1.098236405 | 0.007309186 | 0.042851308 | NA |
| ENSG00000279444 | 1.074452322 | 4.54E-08 | 2.75E-06 | NA |
| ENSG00000279460 | 1.533918816 | 0.005264973 | 0.033909331 | NA |
| ENSG00000279725 | 1.057763716 | 5.08E-05 | 0.000978148 | NA |
| ENSG00000279741 | 1.289322035 | 5.31E-05 | 0.001015549 | NA |
| ENSG00000279839 | 1.192903245 | 0.00176222 | 0.015213167 | NA |
| ENSG00000279847 | -1.254575006 | 0.001768601 | 0.015257072 | C8orf87 |
| ENSG00000279924 | 3.203697542 | 5.86E-06 | 0.000166517 | NA |
| ENSG00000280027 | 1.250444278 | 1.34E-06 | 4.79E-05 | NA |
| ENSG00000280061 | 2.056757441 | 1.10E-07 | 6.02E-06 | NA |
| ENSG00000280217 | 1.304753238 | 1.23E-08 | 9.15E-07 | NA |
| ENSG00000280268 | 1.136482544 | 0.000808104 | 0.008479674 | NA |
| ENSG00000280309 | 1.253436403 | 0.002765045 | 0.021286 | NA |
| ENSG00000280323 | 1.032314632 | 0.001578562 | 0.014073873 | NA |
| ENSG00000280362 | 1.208714155 | 5.66E-05 | 0.001073484 | NA |
| ENSG00000280382 | 1.109063552 | 1.18E-05 | 0.000293363 | NA |
| ENSG00000280623 | 1.186231947 | 0.000110926 | 0.001833754 | NA |
| ENSG00000281162 | -1.033687304 | 7.12E-05 | 0.001293759 | NA |
| ENSG00000281186 | 2.645202962 | 4.65E-17 | 2.77E-14 | LINC00706 |
| ENSG00000281406 | 1.125490305 | 4.03E-08 | 2.50E-06 | NA |
| ENSG00000281613 | -1.324048372 | 4.38E-05 | 0.000864956 | NA |
| ENSG00000281692 | 1.281778329 | 5.21E-08 | 3.12E-06 | PACRG-AS1 |
| ENSG00000281769 | 2.541054994 | 1.71E-07 | 8.74E-06 | NA |

Table.S3 immune-related DEGs

| Gene_id | Adj.P.Val | Gene |
| --- | --- | --- |
| ENSG00000006016 | 2.74E-05 | CRLF1 |
| ENSG00000006128 | 6.90E-06 | TAC1 |
| ENSG00000006606 | 0.025793545 | CCL26 |
| ENSG00000012171 | 0.006558156 | SEMA3B |
| ENSG00000016402 | 1.08E-11 | IL20RA |
| ENSG00000039068 | 0.000173154 | CDH1 |
| ENSG00000043591 | 1.79E-06 | ADRB1 |
| ENSG00000055118 | 2.66E-05 | KCNH2 |
| ENSG00000069482 | 0.004872011 | GAL |
| ENSG00000075223 | 5.26E-05 | SEMA3C |
| ENSG00000078898 | 0.013142548 | BPIFB2 |
| ENSG00000089199 | 8.43E-06 | CHGB |
| ENSG00000089250 | 0.000361407 | NOS1 |
| ENSG00000089685 | 0.001723278 | BIRC5 |
| ENSG00000095752 | 2.15E-05 | IL11 |
| ENSG00000096088 | 2.11E-09 | PGC |
| ENSG00000099937 | 6.98E-14 | SERPIND1 |
| ENSG00000100600 | 2.51E-08 | LGMN |
| ENSG00000100604 | 2.11E-06 | CHGA |
| ENSG00000101144 | 2.18E-08 | BMP7 |
| ENSG00000101349 | 0.002129536 | PAK5 |
| ENSG00000101443 | 3.61E-08 | WFDC2 |
| ENSG00000102466 | 3.91E-07 | FGF14 |
| ENSG00000102678 | 6.35E-05 | FGF9 |
| ENSG00000104938 | 0.002907239 | CLEC4M |
| ENSG00000106018 | 0.006883258 | VIPR2 |
| ENSG00000106128 | 0.033934149 | GHRHR |
| ENSG00000106809 | 0.000729768 | OGN |
| ENSG00000109072 | 0.002218251 | VTN |
| ENSG00000109471 | 0.045141514 | IL2 |
| ENSG00000110680 | 2.32E-35 | CALCA |
| ENSG00000112182 | 0.000190824 | BACH2 |
| ENSG00000112486 | 0.002676008 | CCR6 |
| ENSG00000113494 | 0.01091658 | PRLR |
| ENSG00000113578 | 6.95E-06 | FGF1 |
| ENSG00000113889 | 2.41E-11 | KNG1 |
| ENSG00000113905 | 1.65E-16 | HRG |
| ENSG00000115263 | 0.026357794 | GCG |
| ENSG00000115602 | 0.044698051 | IL1RL1 |
| ENSG00000115884 | 0.001139826 | SDC1 |
| ENSG00000116194 | 8.88E-08 | ANGPTL1 |
| ENSG00000117322 | 1.42E-05 | CR2 |
| ENSG00000117407 | 0.008563842 | ARTN |
| ENSG00000119715 | 0.000192534 | ESRRB |
| ENSG00000120211 | 0.026474311 | INSL4 |
| ENSG00000123999 | 8.89E-13 | INHA |
| ENSG00000124102 | 5.52E-05 | PI3 |
| ENSG00000124107 | 1.17E-12 | SLPI |
| ENSG00000124479 | 0.019591487 | NDP |
| ENSG00000125851 | 0.01699301 | PCSK2 |
| ENSG00000125965 | 1.59E-07 | GDF5 |
| ENSG00000126803 | 0.00034689 | HSPA2 |
| ENSG00000126895 | 0.000173207 | AVPR2 |
| ENSG00000127129 | 0.002173881 | EDN2 |
| ENSG00000129988 | 0.000498422 | LBP |
| ENSG00000131050 | 0.010542403 | BPIFA2 |
| ENSG00000131096 | 2.23E-10 | PYY |
| ENSG00000131910 | 4.62E-08 | NR0B2 |
| ENSG00000132693 | 0.000223668 | CRP |
| ENSG00000132855 | 0.008895688 | ANGPTL3 |
| ENSG00000134640 | 0.046070771 | MTNR1B |
| ENSG00000135346 | 7.26E-10 | CGA |
| ENSG00000137077 | 7.99E-06 | CCL21 |
| ENSG00000137843 | 0.003564983 | PAK6 |
| ENSG00000138039 | 4.25E-06 | LHCGR |
| ENSG00000138696 | 0.000100776 | BMPR1B |
| ENSG00000138798 | 2.19E-05 | EGF |
| ENSG00000140279 | 8.74E-06 | DUOX2 |
| ENSG00000142224 | 0.037042113 | IL19 |
| ENSG00000142273 | 0.00015745 | CBLC |
| ENSG00000143320 | 2.01E-09 | CRABP2 |
| ENSG00000143556 | 0.003018887 | S100A7 |
| ENSG00000143768 | 5.21E-05 | LEFTY2 |
| ENSG00000144648 | 0.000949376 | ACKR2 |
| ENSG00000145147 | 2.11E-06 | SLIT2 |
| ENSG00000145826 | 0.001132105 | LECT2 |
| ENSG00000147571 | 6.35E-09 | CRH |
| ENSG00000148346 | 1.85E-18 | LCN2 |
| ENSG00000149305 | 0.029656752 | HTR3B |
| ENSG00000155918 | 2.80E-07 | RAET1L |
| ENSG00000157005 | 0.044377069 | SST |
| ENSG00000158270 | 0.000652156 | COLEC12 |
| ENSG00000160801 | 0.003500504 | PTH1R |
| ENSG00000160951 | 7.18E-08 | PTGER1 |
| ENSG00000162009 | 0.000481553 | SSTR5 |
| ENSG00000162344 | 0.001012913 | FGF19 |
| ENSG00000162891 | 0.000149628 | IL20 |
| ENSG00000163377 | 0.027533508 | TAFA4 |
| ENSG00000163631 | 2.12E-10 | ALB |
| ENSG00000163993 | 3.81E-08 | S100P |
| ENSG00000164400 | 0.021321937 | CSF2 |
| ENSG00000164825 | 1.81E-11 | DEFB1 |
| ENSG00000166148 | 0.002784317 | AVPR1A |
| ENSG00000166736 | 1.25E-05 | HTR3A |
| ENSG00000166869 | 0.002919379 | CHP2 |
| ENSG00000166923 | 1.20E-05 | GREM1 |
| ENSG00000167244 | 5.76E-12 | IGF2 |
| ENSG00000168329 | 0.005939235 | CX3CR1 |
| ENSG00000168509 | 1.32E-07 | HJV |
| ENSG00000168621 | 2.90E-05 | GDNF |
| ENSG00000169297 | 0.000995945 | NR0B1 |
| ENSG00000169750 | 1.95E-06 | RAC3 |
| ENSG00000169752 | 1.90E-06 | NRG4 |
| ENSG00000171136 | 0.00350883 | RLN3 |
| ENSG00000171819 | 2.62E-05 | ANGPTL7 |
| ENSG00000172156 | 0.044695797 | CCL11 |
| ENSG00000174697 | 0.006590563 | LEP |
| ENSG00000176919 | 0.001439074 | C8G |
| ENSG00000177398 | 0.001608013 | UMODL1 |
| ENSG00000177984 | 0.047431902 | LCN15 |
| ENSG00000178591 | 0.037027894 | DEFB125 |
| ENSG00000180772 | 0.024207066 | AGTR2 |
| ENSG00000180875 | 0.007144299 | GREM2 |
| ENSG00000181092 | 0.038249568 | ADIPOQ |
| ENSG00000181374 | 0.042688328 | CCL13 |
| ENSG00000182687 | 1.32E-06 | GALR2 |
| ENSG00000183844 | 1.36E-06 | FAM3B |
| ENSG00000184599 | 0.007620678 | TAFA3 |
| ENSG00000184925 | 1.21E-05 | LCN12 |
| ENSG00000186090 | 0.022571789 | HTR3D |
| ENSG00000186458 | 2.52E-05 | DEFB132 |
| ENSG00000187094 | 0.008894963 | CCK |
| ENSG00000187922 | 0.000178803 | LCN10 |
| ENSG00000189058 | 0.002550643 | APOD |
| ENSG00000189334 | 1.17E-07 | S100A14 |
| ENSG00000189377 | 0.010129626 | CXCL17 |
| ENSG00000196136 | 3.79E-07 | SERPINA3 |
| ENSG00000196420 | 4.40E-11 | S100A5 |
| ENSG00000196468 | 0.04962463 | FGF16 |
| ENSG00000196754 | 8.84E-12 | S100A2 |
| ENSG00000197943 | 7.25E-07 | PLCG2 |
| ENSG00000197956 | 1.00E-05 | S100A6 |
| ENSG00000198049 | 0.000699479 | AVPR1B |
| ENSG00000215182 | 0.010489844 | MUC5AC |
| ENSG00000215644 | 4.03E-08 | GCGR |
| ENSG00000225950 | 0.026278749 | NTF4 |
| ENSG00000228278 | 1.43E-05 | ORM2 |
| ENSG00000229314 | 0.001771537 | ORM1 |
| ENSG00000237541 | 0.027642439 | HLA-DQA1 |
| ENSG00000243709 | 2.95E-12 | LEFTY1 |
| ENSG00000258947 | 8.15E-07 | TUBB3 |

Table S2 The validation cohort patients' clinical information

| ID | gender | age | clinical staging | status | rs | risk | months |
| --- | --- | --- | --- | --- | --- | --- | --- |
| 43832 | MALE | 69.1 | Stage IV | 1 | -0.05663 | high-risk | 4.076712 |
| 43833 | FEMALE | 68.6 | Stage I | 0 | -0.28199 | high-risk | 5.983562 |
| 43834 | MALE | 67.3 | Stage II | 1 | -0.31934 | high-risk | 6.115068 |
| 43835 | MALE | 66.6 | Stage I | 0 | -0.00016 | high-risk | 6.772603 |
| 43836 | FEMALE | 77.5 | Stage III | 0 | -0.26577 | high-risk | 7.232877 |
| 43837 | MALE | 58 | Stage II | 0 | -0.04557 | high-risk | 8.745205 |
| 43839 | MALE | 59.1 | Stage I | 1 | -0.40756 | high-risk | 9.008219 |
| 43840 | MALE | 57.3 | Stage II | 1 | -0.18726 | high-risk | 9.139726 |
| 43841 | FEMALE | 68 | Stage II | 0 | -0.00918 | high-risk | 9.271233 |
| 43842 | MALE | 70.4 | Stage I | 0 | -0.16256 | high-risk | 10.65205 |
| 43843 | FEMALE | 52.4 | Stage III | 0 | -0.52044 | high-risk | 10.91507 |
| 43844 | MALE | 51.4 | Stage II | 1 | -0.12678 | high-risk | 10.91507 |
| 43845 | MALE | 53.3 | Stage I | 0 | -0.4453 | high-risk | 11.04658 |
| 43846 | MALE | 51.6 | Stage I | 0 | -0.41679 | high-risk | 11.63836 |
| 43847 | FEMALE | 52.4 | Stage I | 0 | -0.3594 | high-risk | 13.54521 |
| 43848 | FEMALE | 47.4 | Stage I | 0 | -0.28749 | high-risk | 13.67671 |
| 43849 | FEMALE | 79.9 | Stage I | 1 | -0.28603 | high-risk | 13.87397 |
| 43850 | MALE | 75.8 | Stage I | 1 | -0.19051 | high-risk | 10.94795 |
| 43852 | MALE | 86.6 | Stage IV | 0 | -0.2928 | high-risk | 11.53973 |
| 43854 | MALE | 41.8 | Stage IV | 1 | -0.35402 | high-risk | 12.08219 |
| 43855 | MALE | 75.4 | Stage IV | 0 | -0.1961 | high-risk | 12.23014 |
| 43856 | FEMALE | 79.3 | Stage III | 0 | -0.36511 | high-risk | 15.0411 |
| 43857 | MALE | 68.8 | Stage III | 1 | -0.05202 | high-risk | 15.33699 |
| 43859 | MALE | 76.6 | Stage III | 0 | 0.029846 | high-risk | 15.73151 |
| 43861 | FEMALE | 34.1 | Stage IV | 1 | 0.089061 | high-risk | 16.27397 |
| 1-32 | MALE | 42.6 | Stage IV | 1 | -0.11201 | high-risk | 16.42192 |
| 1-33 | MALE | NA | Stage IV | 1 | -0.37288 | high-risk | 17.85205 |
| 1-34 | MALE | 62.5 | Stage IV | 0 | -0.40948 | high-risk | 18 |
| 1-35 | MALE | 57.8 | Stage IV | 0 | 0.464831 | high-risk | 18.19726 |
| 1-37 | FEMALE | 82.9 | Stage IV | 0 | -0.50414 | high-risk | 21.35342 |
| 1-38 | MALE | 60.2 | Stage III | 1 | -0.18559 | high-risk | 21.94521 |
| 1-40 | FEMALE | 50.9 | Stage III | 1 | -0.11229 | high-risk | 22.38904 |
| 1-41 | FEMALE | 46.3 | Stage III | 1 | -0.20674 | high-risk | 23.91781 |
| 1-43 | MALE | 72.6 | Stage IV | 0 | -0.22276 | high-risk | 25.2 |
| 1-44 | MALE | 48.3 | Stage III | 0 | -0.47058 | high-risk | 27.1726 |
| 1-45 | MALE | 64.5 | Stage IV | 0 | -0.15993 | high-risk | 27.61644 |
| 1-46 | FEMALE | 54.7 | Stage III | 1 | -0.01827 | high-risk | 27.66575 |
| 1-48 | FEMALE | 51.8 | Stage III | 0 | -0.29466 | high-risk | 27.76438 |
| 1-50 | FEMALE | 51.9 | Stage III | 1 | -0.421 | high-risk | 28.1589 |
| 1-51 | MALE | 60.5 | Stage III | 1 | -0.48136 | high-risk | 28.94795 |
| 1-52 | MALE | 54.3 | Stage IV | 0 | -0.12104 | high-risk | 29.73699 |
| 1-54 | FEMALE | 69.1 | Stage III | 1 | -0.34842 | high-risk | 31.4137 |
| 1-55 | MALE | 52.9 | Stage II | 0 | -0.18327 | high-risk | 32.64658 |
| 43862 | FEMALE | 46.9 | Stage III | 0 | -0.18156 | high-risk | 33.3863 |
| 43864 | FEMALE | 49.1 | Stage II | 0 | -0.35506 | high-risk | 35.21096 |
| 43865 | MALE | 47.1 | Stage IV | 1 | -0.18751 | high-risk | 30.85808 |
| 43867 | MALE | 57.3 | Stage III | 0 | -0.23369 | high-risk | 31.02904 |
| 43868 | MALE | 49.2 | Stage II | 1 | -0.46676 | high-risk | 31.07178 |
| 43869 | FEMALE | 78.6 | Stage I | 1 | -0.48595 | high-risk | 31.4137 |
| 43870 | MALE | 59.1 | Stage I | 0 | -0.18529 | high-risk | 32.01205 |
| 43871 | MALE | 75 | Stage III | 1 | -0.32694 | high-risk | 32.82411 |
| 43873 | FEMALE | 75.4 | Stage IV | 1 | -0.12163 | high-risk | 33.42247 |
| 43874 | MALE | 37.1 | Stage I | 1 | -0.16729 | high-risk | 35.13205 |
| 43875 | MALE | 65.7 | Stage I | 0 | -0.26055 | high-risk | 35.90137 |
| 43876 | FEMALE | 68.3 | Stage IV | 1 | -0.49962 | high-risk | 37.82466 |
| 43877 | MALE | 37.7 | Stage I | 0 | -0.46701 | high-risk | 38.89315 |
| 43878 | FEMALE | 65.9 | Stage I | 1 | -0.27071 | high-risk | 39.61973 |
| 43880 | MALE | 62.1 | Stage I | 1 | -0.05908 | high-risk | 40.43178 |
| 43881 | MALE | 54.4 | Stage IV | 0 | -0.18169 | high-risk | 40.64548 |
| 43883 | MALE | 62 | Stage III | 0 | -0.23715 | high-risk | 40.90192 |
| 43884 | MALE | 62.6 | Stage III | 0 | -0.24555 | high-risk | 43.33808 |
| 43885 | MALE | 48.9 | Stage IV | 0 | -0.31331 | high-risk | 45.04767 |
| 43887 | MALE | 72.6 | Stage IV | 1 | -0.37182 | high-risk | 46.67178 |
| 43888 | MALE | 40.7 | Stage IV | 1 | -0.43792 | high-risk | 46.88548 |
| 43889 | MALE | 58.5 | Stage IV | 0 | -0.25133 | high-risk | 47.09918 |
| 2-30 | FEMALE | 45.4 | Stage III | 0 | -0.48744 | high-risk | 48.72329 |
| 2-32 | MALE | 80.8 | Stage III | 1 | -0.23573 | high-risk | 50.90301 |
| 2-33 | MALE | 69.8 | Stage II | 0 | -0.18727 | high-risk | 53.72384 |
| 2-34 | FEMALE | 84 | Stage I | 0 | 0.071767 | high-risk | 56.16 |
| 2-35 | MALE | 85.9 | Stage III | 1 | -0.5044 | high-risk | 56.28822 |
| 2-38 | MALE | 48.9 | Stage I | 0 | -0.40317 | high-risk | 58.59616 |
| 2-39 | MALE | 58.3 | Stage IV | 0 | -0.41467 | high-risk | 60.90411 |
| 2-41 | FEMALE | 84.6 | Stage III | 1 | -0.08186 | high-risk | 62.52822 |
| 2-42 | MALE | 71.8 | Stage I | 0 | -0.43892 | high-risk | 63.16932 |
| 2-44 | MALE | 48.8 | Stage IV | 0 | -0.46646 | high-risk | 63.55397 |
| 2-45 | FEMALE | 48.1 | Stage III | 0 | 0.005136 | high-risk | 63.89589 |
| 2-46 | MALE | 58.3 | Stage I | 0 | -0.2129 | high-risk | 63.93863 |
| 2-47 | FEMALE | 72.5 | Stage IV | 0 | -0.45504 | high-risk | 64.96438 |
| 2-48 | MALE | 71.9 | Stage IV | 1 | -0.41294 | high-risk | 67.69973 |
| 2-49 | FEMALE | 63.1 | Stage III | 0 | -0.31362 | high-risk | 68.34082 |
| 2-50 | MALE | 35.4 | Stage III | 1 | -0.22322 | high-risk | 69.45205 |
| 2-51 | MALE | 46 | Stage III | 1 | -0.44895 | high-risk | 69.49479 |
| 2-53 | FEMALE | 65.7 | Stage I | 0 | -0.3901 | high-risk | 72.27288 |
| 2-54 | MALE | 55.3 | Stage III | 1 | -0.14481 | high-risk | 73.68329 |
| 2-56 | MALE | 73 | Stage III | 0 | -0.26465 | high-risk | 81.46192 |
| 2-57 | MALE | 72.8 | Stage I | 1 | -0.12279 | high-risk | 81.71836 |
| 2-59 | MALE | 58.4 | Stage I | 0 | -0.24586 | high-risk | 83.04329 |
| 2-60 | MALE | 46.1 | Stage I | 1 | -0.32391 | high-risk | 84.62466 |
| 2-62 | MALE | 75.1 | Stage IV | 1 | -0.23487 | high-risk | 98.25863 |
| 2-63 | FEMALE | 74.6 | Stage III | 0 | -0.20545 | high-risk | 108.9008 |
| 2-64 | MALE | 60.4 | Stage III | 1 | -0.39894 | high-risk | 170.1109 |
| 43831 | FEMALE | 66.4 | Stage I | 0 | -0.70466 | low-risk | 5.198203 |
| 43838 | MALE | 51.8 | Stage III | 0 | -0.58226 | low-risk | 11.50422 |
| 43851 | MALE | 61.2 | Stage IV | 1 | -0.60546 | low-risk | 14.31636 |
| 43853 | MALE | 63.9 | Stage III | 1 | -0.77617 | low-risk | 15.46678 |
| 43858 | FEMALE | 75.1 | Stage I | 0 | -0.70308 | low-risk | 20.13238 |
| 43860 | MALE | 77 | Stage I | 1 | -0.53896 | low-risk | 21.02716 |
| 1-36 | FEMALE | 76.4 | Stage I | 0 | -0.54101 | low-risk | 23.64756 |
| 1-39 | MALE | 81.8 | Stage I | 0 | -0.60722 | low-risk | 29.0162 |
| 1-42 | FEMALE | 57.3 | Stage I | 0 | -0.59796 | low-risk | 32.02008 |
| 1-47 | FEMALE | 47.7 | Stage III | 1 | -0.54514 | low-risk | 35.98264 |
| 1-49 | MALE | 48.7 | Stage IV | 0 | -0.63268 | low-risk | 36.23829 |
| 1-53 | MALE | 68.5 | Stage I | 0 | -0.54721 | low-risk | 40.26477 |
| 43863 | FEMALE | 58.6 | Stage I | 1 | -0.54814 | low-risk | 45.31384 |
| 43866 | FEMALE | 65.4 | Stage III | 0 | -0.59591 | low-risk | 40.04747 |
| 43872 | MALE | 49.7 | Stage I | 0 | -0.67825 | low-risk | 42.98317 |
| 43879 | MALE | 81.8 | Stage III | 0 | -0.57368 | low-risk | 51.62412 |
| 43882 | MALE | 68.1 | Stage I | 1 | -0.66274 | low-risk | 52.73193 |
| 43886 | MALE | 60.9 | Stage III | 0 | -0.56984 | low-risk | 59.32342 |
| 43890 | MALE | 68.5 | Stage I | 0 | -0.78189 | low-risk | 62.59147 |
| 2-31 | MALE | 78.8 | Stage I | 0 | -0.59195 | low-risk | 64.14241 |
| 2-36 | MALE | 88.5 | Stage I | 0 | -0.77178 | low-risk | 73.61422 |
| 2-37 | MALE | 49.7 | Stage I | 0 | -0.68829 | low-risk | 75.05438 |
| 2-40 | MALE | 78 | Stage III | 0 | -0.70585 | low-risk | 80.31649 |
| 2-43 | MALE | 79.3 | Stage I | 0 | -0.60539 | low-risk | 82.36595 |
| 2-52 | MALE | 82.7 | Stage I | 0 | -0.6394 | low-risk | 92.50244 |
| 2-55 | MALE | 49.9 | Stage I | 0 | -0.90451 | low-risk | 97.93073 |
| 2-58 | MALE | 61.6 | Stage II | 1 | -0.75285 | low-risk | 105.9624 |
| 2-61 | MALE | 63.4 | Stage I | 0 | -0.66948 | low-risk | 120.3086 |
