# Supplementary figures and images for: Development and validation of a VHL-associated immune prognostic signature for clear cell renal cell carcinoma
Source: Cancer Cell Int. 2020 Dec 7;20:584. doi: 10.1186/s12935-020-01670-5 (PMC7720505; doi:10.1186/s12935-020-01670-5)

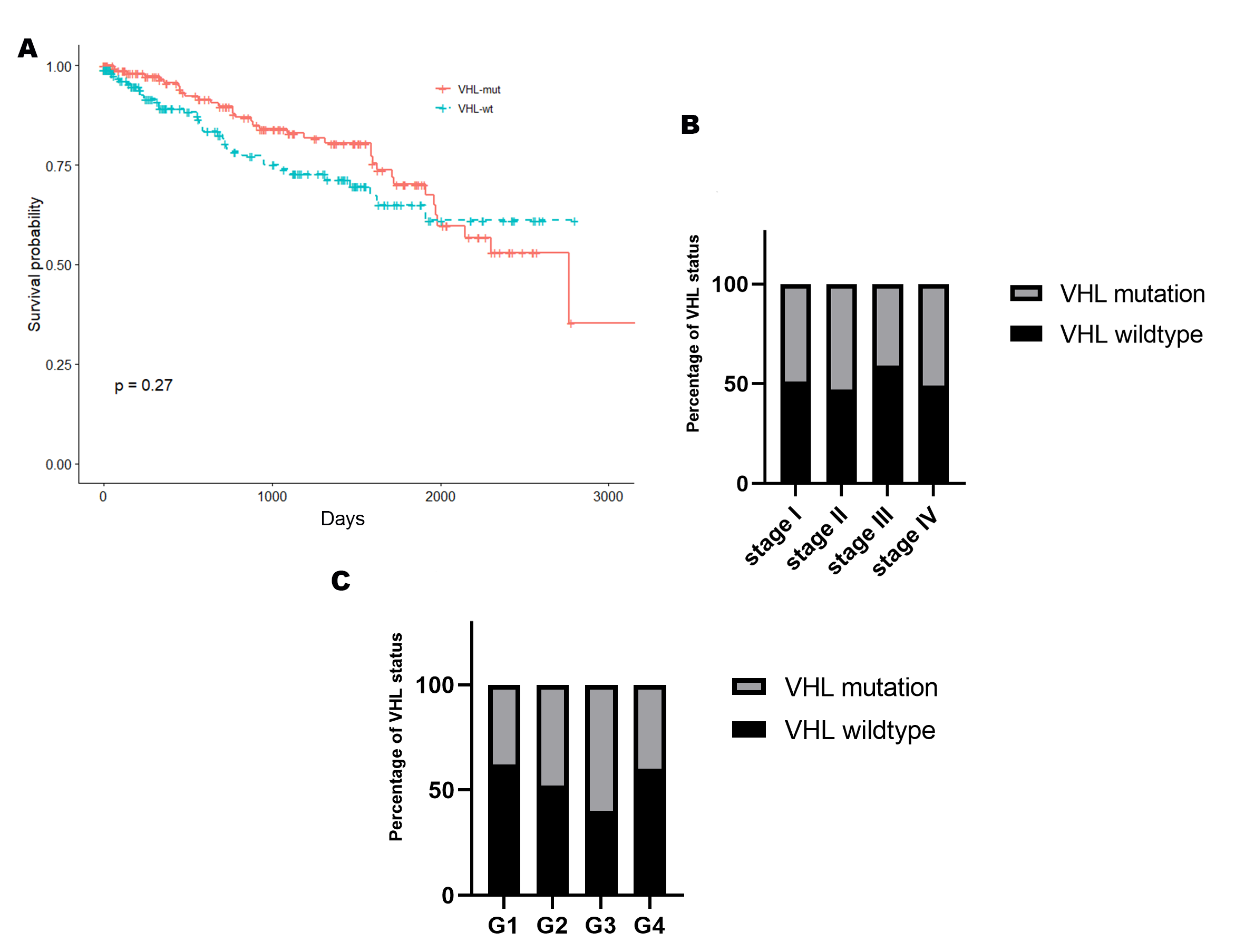

Supplement: Supplementary file 2 — Additional file 2: Figure S1. a The Kaplan-Meier survival analysis in the TCGA-KIRC dataset between VHL mutation and wildtype patients. b The percentage of VHL status in KIRC patients with different pathological staging and c Fuhrman grading. [file 12935_2020_1670_MOESM2_ESM.tif]

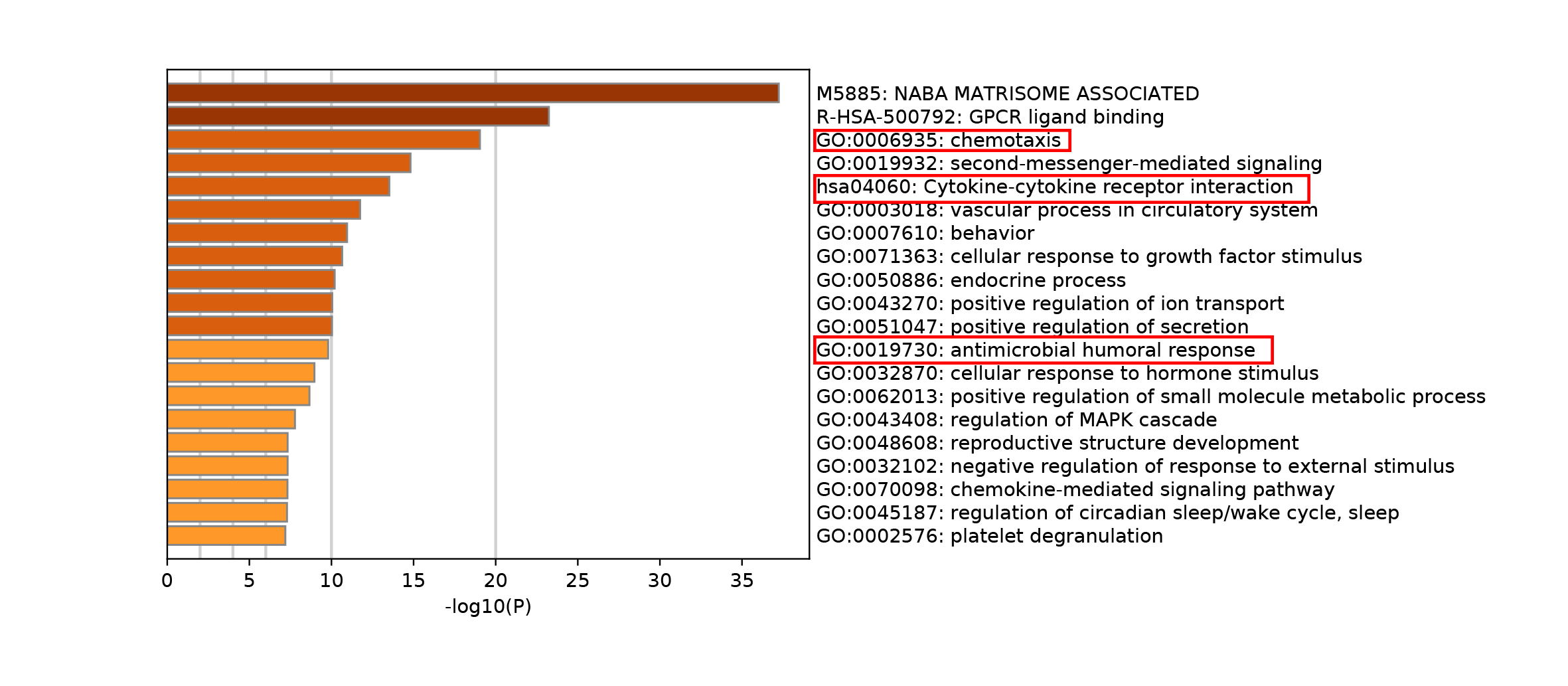

Supplement: Supplementary file 3 — Additional file 3: Figure S2. The pathway enrichment of immune-related DGEs. The red rectangles indicate immune-related pathways. [file 12935_2020_1670_MOESM3_ESM.tif]

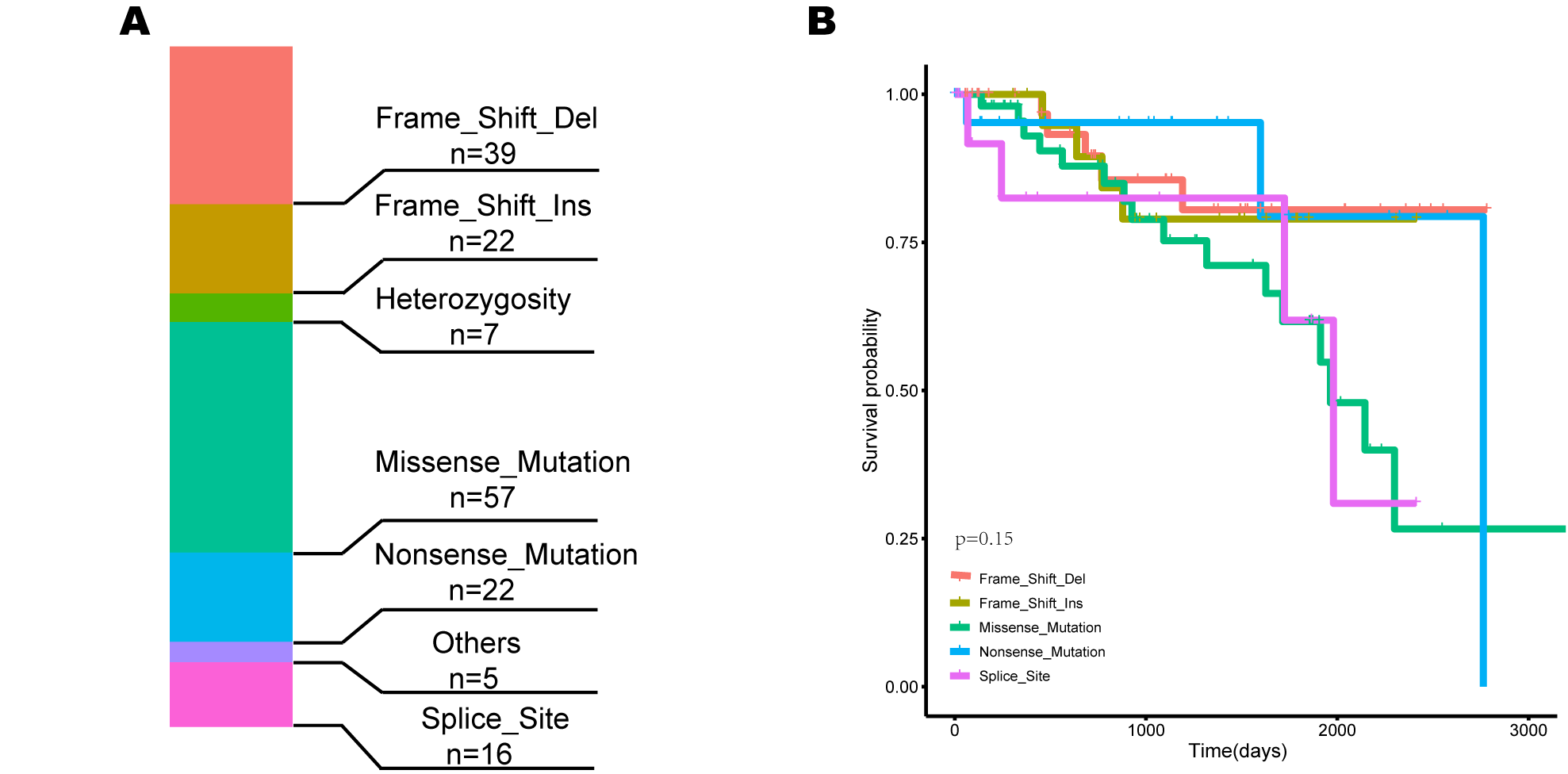

Supplement: Supplementary file 4 — Additional file 4: Figure S3. Tthe hierarchical analysis of VHL mutation. [file 12935_2020_1670_MOESM4_ESM.tif]

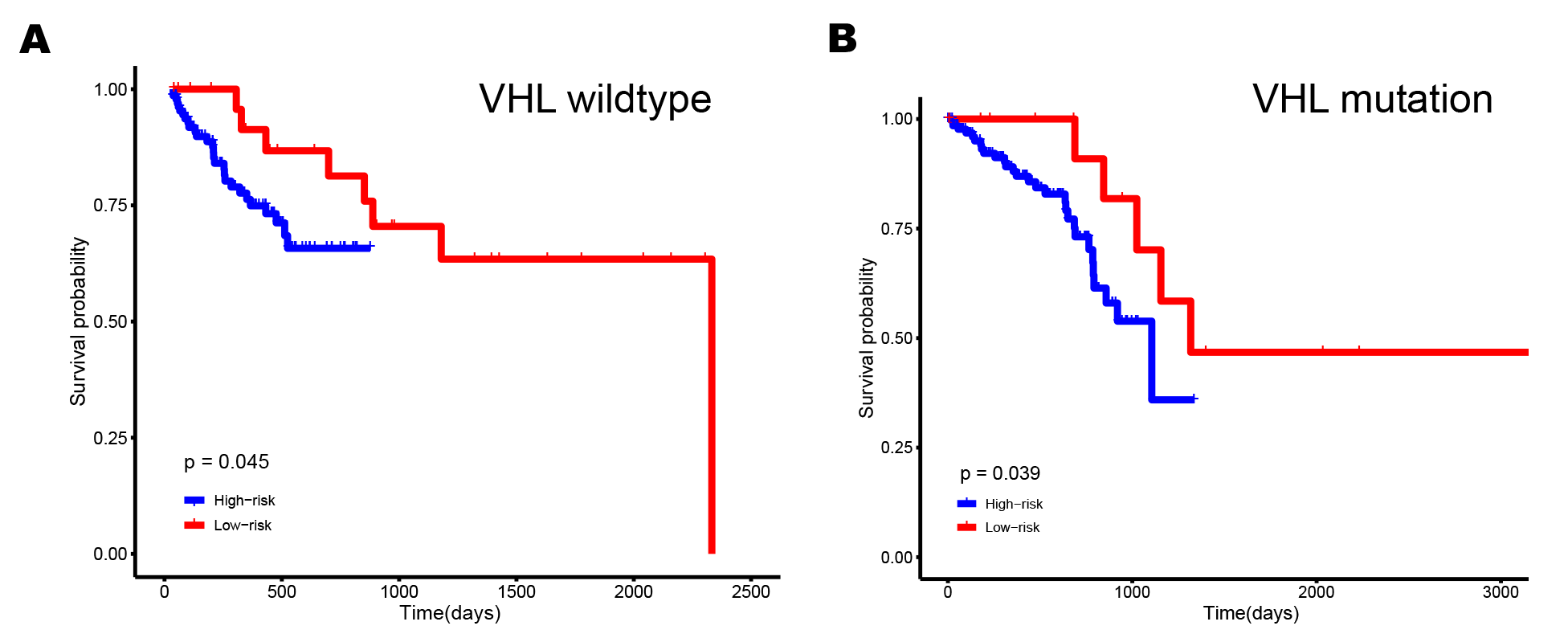

Supplement: Supplementary file 5 — Additional file 5: Figure S4. Survival analysis of immune related signature in VHL wt and WHL mutation subgroups. [file 12935_2020_1670_MOESM5_ESM.tif]
